# Endosome maturation links PI3Kα signaling to lysosome repopulation during basal autophagy

**DOI:** 10.15252/embj.2021110398

**Published:** 2022-08-15

**Authors:** Samuel J Rodgers, Emily I Jones, Senthil Arumugam, Sabryn A Hamila, Jill Danne, Rajendra Gurung, Matthew J Eramo, Randini Nanayakkara, Georg Ramm, Meagan J McGrath, Christina A Mitchell

**Affiliations:** ^1^ Department of Biochemistry and Molecular Biology, Biomedicine Discovery Institute Monash University Clayton VIC Australia; ^2^ Department of Anatomy and Developmental Biology, Biomedicine Discovery Institute Monash University Clayton VIC Australia; ^3^ European Molecular Biological Laboratory Australia Monash University Clayton VIC Australia; ^4^ Monash Ramaciotti Centre for Cryo Electron Microscopy, A Node of Microscopy Australia Monash University Clayton VIC Australia

**Keywords:** autophagy, INPP4B, lysosome, PI3Kα, PIKfyve, Autophagy & Cell Death

## Abstract

Autophagy depends on the repopulation of lysosomes to degrade intracellular components and recycle nutrients. How cells co‐ordinate lysosome repopulation during basal autophagy, which occurs constitutively under nutrient‐rich conditions, is unknown. Here, we identify an endosome‐dependent phosphoinositide pathway that links PI3Kα signaling to lysosome repopulation during basal autophagy. We show that PI3Kα‐derived PI(3)P generated by INPP4B on late endosomes was required for basal but not starvation‐induced autophagic degradation. PI(3)P signals were maintained as late endosomes matured into endolysosomes, and served as the substrate for the 5‐kinase, PIKfyve, to generate PI(3,5)P_2_. The SNX‐BAR protein, SNX2, was recruited to endolysosomes by PI(3,5)P_2_ and promoted lysosome reformation. Inhibition of INPP4B/PIKfyve‐dependent lysosome reformation reduced autophagic clearance of protein aggregates during proteotoxic stress leading to increased cytotoxicity. Therefore under nutrient‐rich conditions, PI3Kα, INPP4B, and PIKfyve sequentially contribute to basal autophagic degradation and protection from proteotoxic stress via PI(3,5)P_2_‐dependent lysosome reformation from endolysosomes. These findings reveal that endosome maturation couples PI3Kα signaling to lysosome reformation during basal autophagy.

## Graphical abstract

Sequential phosphoinositide conversion by PI3Kα, INPP4B and PIKfyve is necessary for autophagic degradation and protein aggregate clearance under nutrient‐rich conditions.

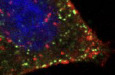

## Introduction

Autophagy is a highly conserved pathway that degrades intracellular components and recycles nutrients. Autophagy occurs constitutively in almost all eukaryotic cells to ensure organelle quality control, and is upregulated in response to nutrient deficiency or stress to mobilize amino acids and promote cytoprotection (Klionsky *et al*, 2021). Autophagic cargo is encapsulated and sequestered within autophagosomes that subsequently fuse with lysosomes to form autolysosomes, in which cargo is broken down into macromolecules that are reutilized by the cell (Dikic & Elazar, 2018). A dynamic equilibrium of autophagosomes and lysosomes must be maintained for sustained autophagy and to allow adaptive autophagy responses. If lysosomes are not sufficiently repopulated during autophagy, autophagosomes accumulate and autophagic function is reduced (McGrath *et al*, 2021). There has been significant progress in understanding how lysosomes are repopulated during starvation‐induced autophagy via transcription factor EB (TFEB)/transcription factor E3 (TFE3)‐dependent lysosome biogenesis (Settembre *et al*, 2011, 2012) and autophagic lysosome reformation (ALR) pathways (Yu *et al*, 2010). However, these pathways do not operate during basal autophagy, which occurs under nutrient‐rich conditions to maintain cellular homeostasis and is associated with longevity and protection against neurodegeneration (Hara *et al*, 2006; Komatsu *et al*, 2006; Nakamura & Yoshimori, 2018). Therefore, it remains unclear how cells co‐ordinate lysosome repopulation during basal autophagy.

Phosphoinositide 3‐kinases (PI3Ks) generate phosphoinositides on intracellular membranes that regulate many aspects of autophagy. In the canonical pathway that operates during starvation, Unc‐51 like autophagy activating kinase (ULK1) activates the class III PI3K complex I (Vps34, p150, Beclin‐1, and ATG14) generating phosphatidylinositol 3‐phosphate (PI(3)P) on omegasomes, autophagosome precursors derived from endoplasmic reticulum membranes (Axe *et al*, 2008; Russell *et al*, 2013). PI(3)P recruits effector proteins that drive elongation and closure of emerging autophagosome membranes, leading to the sequestration of autophagic cargo within autophagosomes (Axe *et al*, 2008; Dooley *et al*, 2014). PI(3)P also interacts with tectonin beta‐propeller repeat containing protein 1 (TECPR1) to facilitate autophagosome–lysosome fusion (Chen *et al*, 2012), and contributes to ALR to facilitate lysosome repopulation during starvation‐induced autophagy (Munson *et al*, 2015). The class II PI3K, PI3KC2α, is also required for shear stress‐induced autophagy via PI(3)P generation at primary cilia (Boukhalfa *et al*, 2020), whereas PI3KC2β contributes to autophagic degradation during starvation via phosphatidyinositol 3,4‐bisphosphate (PI(3,4)P_2_) generation on lysosomes (Marat *et al*, 2017).

Alternatively, inactivation of class I PI3K signaling during starvation contributes to mTOR‐dependent autophagy activation (Manning & Toker, 2017). In response to growth factor stimulation, class I PI3K generates phosphatidylinositol 3,4,5‐trisphosphate (PI(3,4,5)P_3_) on the inner leaflet of the plasma membrane, which is hydrolyzed by inositol polyphosphate 5‐phosphatases to PI(3,4)P_2_ (Rodgers *et al*, 2017). PI(3,4,5)P_3_ and PI(3,4)P_2_ together recruit AKT (James *et al*, 1996; Ma *et al*, 2008) to the inner wall of the plasma membrane, where it is activated by phosphorylation and subsequently activates the autophagy repressor mTOR (Inoki *et al*, 2002). Paradoxically, the class I PI3K p110β catalytic subunit promotes autophagosome formation by stimulating class III PI3K complex activity via Rab5 binding (Dou *et al*, 2010, 2013), suggesting that the contribution of class I PI3K signaling to autophagy regulation is complex.

Interestingly up to 30% of cellular PI(3)P is generated downstream of growth factor‐stimulated class I PI3K signaling via hydrolysis of PI(3,4)P_2_ by inositol polyphosphate 4‐phosphatase type I (INPP4A) and type II (INPP4B) (Shin *et al*, 2005; Ikonomov *et al*, 2015). It is not known whether class I PI3K‐derived PI(3)P generation has a functional role in autophagy. INPP4A and INPP4B convert PI(3,4)P_2_ to PI(3)P on distinct endosomal compartments promoting endocytosis and late endosome trafficking, respectively (Ivetac *et al*, 2005; Shin *et al*, 2005; Li Chew *et al*, 2015; Daste *et al*, 2017; Liu *et al*, 2018, 2020; Rodgers *et al*, 2021). INPP4B is recruited to late endosomes via Rab7 binding, where it generates PI(3)P downstream of PI3Kα to enhance Hrs‐dependent late endosome formation (Rodgers *et al*, 2021). Thereby INPP4B promotes lysosomal degradation of endocytic cargoes including EGFR and GSK3β leading to AKT/MEK signaling suppression or Wnt/β‐catenin signaling activation, respectively (Liu *et al*, 2020; Rodgers *et al*, 2021).

Here, we investigated the role class I PI3K‐dependent PI(3)P generation plays in autophagy. We demonstrate that PI3Kα‐derived PI(3)P synthesis by INPP4B on endosomes is required for lysosome reformation from endolysosomes, basal autophagic degradation, and protection from proteotoxic stress, which is functionally distinct from the canonical Vps34‐generated pool of PI(3)P. Mechanistically, INPP4B‐generated PI(3)P signals are retained as endosomes mature into endolysosomes, and act as a substrate for PI(3,5)P_2_ synthesis by PIKfyve. PI(3,5)P_2_ recruits the SNX‐BAR protein, SNX2, to promote endolysosome membrane tubulation, and thereby lysosome reformation. Therefore, our investigation reveals an endosome‐dependent phosphoinositide conversion pathway that couples PI3Kα signaling to lysosome repopulation during basal autophagy.

## Results

### INPP4B promotes PI3Kα‐dependent basal autophagic degradation independent of Vps34

**Figure 1:**
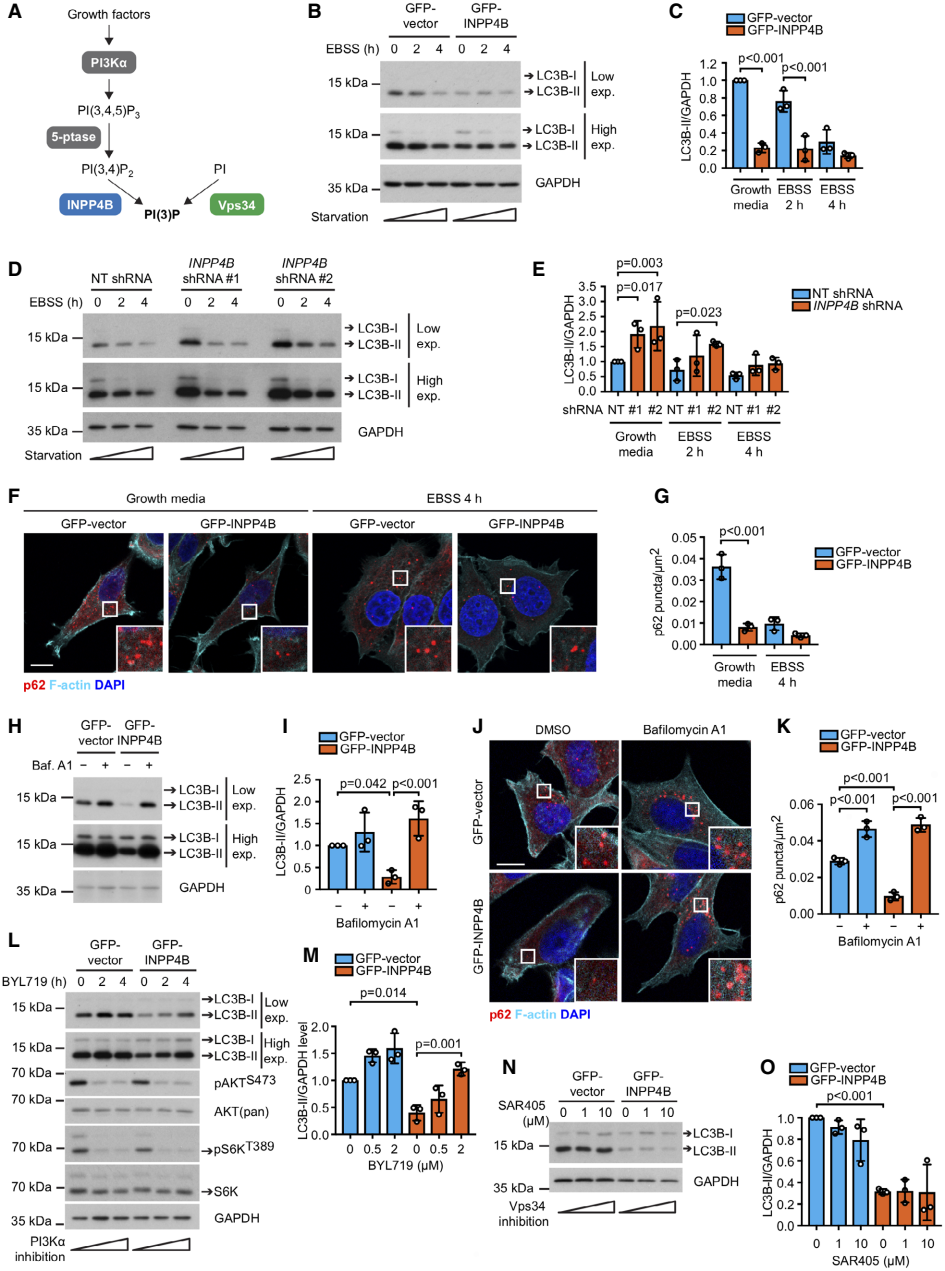
INPP4B promotes PI3Kα‐dependent basal autophagic degradation independent of Vps34 A
PI(3)P is synthesized directly by the class III PI3K Vps34, or via the sequential actions of PI3Kα, inositol polyphosphate 5‐phosphatses, and INPP4B in response to growth factor stimulation.B, C
MCF‐7 cells expressing GFP‐INPP4B or GFP‐vector were cultured in growth media or EBSS for the indicated times, then lysed and immunoblotted with LC3B antibodies and GAPDH antibodies as a loading control (B). Data represent the relative LC3B‐II levels normalized to GAPDH, and expressed relative to growth media‐treated GFP‐vector cells which were assigned an arbitrary value of 1 (*n* = 3 experiments) (C).D, E
MCF‐7 cells expressing nontargeted (NT), *INPP4B* #1, or *INPP4B* #2 shRNA were cultured in growth media or EBSS for the indicated times, then lysed and immunoblotted with LC3B antibodies and GAPDH antibodies as a loading control (D). Data represent the relative LC3B‐II levels normalized to GAPDH, and expressed relative to growth media‐treated NT shRNA cells which were assigned an arbitrary value of 1 (*n* = 3 experiments) (E).F, G
MCF‐7 cells expressing GFP‐INPP4B or GFP‐vector were cultured in growth media or EBSS for 4 h, then fixed and immunostained with p62 antibodies, and co‐stained with DAPI and phalloidin (F). Data represent the number of p62^+^ puncta relative to cell area (μm^2^) (*n* = 3 experiments, > 50 cells/experiment) (G).H, I
MCF‐7 cells expressing GFP‐INPP4B or GFP‐vector were treated with 100 nM bafilomycin A1 or DMSO as a vehicle control for 4 h, then lysed and immunoblotted with LC3B antibodies and GAPDH antibodies as a loading control (H). Data represent the relative LC3B‐II levels normalized to GAPDH, and expressed relative to DMSO‐treated GFP‐vector cells which were assigned an arbitrary value of 1 (I) (*n* = 3 experiments).J, K
MCF‐7 cells expressing GFP‐INPP4B or GFP‐vector were treated with 100 nM of bafilomycin A1 or DMSO as a vehicle control for 4 h, then fixed and immunostained with p62 antibodies, and co‐stained with DAPI and phalloidin (J). Data represent the number of p62^+^ puncta relative to cell area (μm^2^) (*n* = 3 experiments, > 50 cells/experiment) (K).L, M
MCF‐7 cells expressing GFP‐INPP4B or GFP‐vector were treated with 2 μM BYL719 (PI3Kα inhibitor) or DMSO as a vehicle control for the indicated times. Cells were lysed and immunoblotted with LC3B, pAKT^S473^, AKT(pan), pS6K^T389^, or S6K antibodies and GAPDH antibodies as a loading control (L). Data represent the relative LC3B‐II levels normalized to GAPDH, and expressed relative to DMSO‐treated GFP‐vector cells which were assigned an arbitrary value of 1 (*n* = 3 experiments) (M).N, O
MCF‐7 cells expressing GFP‐INPP4B or GFP‐vector were treated with 1 or 10 μM of SAR405 (Vps34 inhibitor) or DMSO as a vehicle control for 4 h. Cells were lysed and immunoblotted with LC3B antibodies and GAPDH antibodies as a loading control (N). Data represent the relative LC3B‐II levels normalized to GAPDH, and expressed relative to DMSO‐treated GFP‐vector cells which were assigned an arbitrary value of 1 (*n* = 3 experiments) (O). Data information: Data are presented as mean ± SD. The insets at the lower right of each image are higher power regions of the boxed areas. Scale bar is 10 μm in (F, J). *P* values determined by one‐way ANOVA with Tukey *post hoc* test in (C, G, I, K, M, O), or by one‐way ANOVA in (E). Source data are available online for this figure.

The majority of cellular PI(3)P is generated via phosphorylation of phosphatidylinositol (PI) by Vps34, which promotes autophagosome formation and the recruitment of autophagic cargo in response to nutrient deprivation (Dooley *et al*, 2014). However, PI(3)P is also generated downstream of PI3Kα via hydrolysis of PI(3,4)P_2_ by the 4‐phosphatase INPP4B under nutrient‐rich conditions (Shin *et al*, 2005; Gewinner *et al*, 2009; Ikonomov *et al*, 2015; Rodgers *et al*, 2021; Fig 1A). It is unknown whether this alternate PI(3)P pool also contributes to autophagy regulation. To decipher the role INPP4B‐generated PI(3)P plays, we undertook a systematic evaluation of the autophagy pathway in MCF‐7 cells, which express endogenous INPP4B but not INPP4A (Fedele *et al*, 2010) and harbor a hyperactivating PIK3CA^E545K^ mutation. Lipidated LC3B‐II, which associates with the autophagosome membrane and corresponds to the relative number of autophagosomes (Mizushima & Yoshimori, 2007), was assessed under growth media conditions (basal autophagy) or following nutrient‐free EBSS treatment which activates starvation‐induced autophagy. Interestingly, ectopic GFP‐INPP4B expression (Appendix Fig S1A) decreased LC3B‐II protein levels under growth conditions, however, there was no sustained difference in LC3B‐II between GFP‐INPP4B and GFP‐vector cells during prolonged starvation‐induced autophagy (Fig 1B and C). In contrast, *INPP4B* shRNA depletion (Appendix Fig S1B and C) increased LC3B‐II under growth conditions, but not following prolonged starvation‐induced autophagy (Fig 1D and E). *INPP4B* siRNA depletion also increased LC3B‐II levels under growth conditions in HeLa and HEK293T cells (Appendix Fig S1D–F), indicating that INPP4B regulation of basal autophagy is conserved across multiple independent cell lines. GFP‐INPP4B also significantly decreased the number of Sequesterome 1 (SQSTM1, better known as p62)‐positive autophagosomes under growth conditions, but had little effect on autophagosome numbers following EBSS treatment (Fig 1F and G). Collectively, this data suggests that INPP4B selectively reduces the number of autophagosomes during basal autophagy but not following activation of starvation‐enhanced autophagy. Furthermore, the number of p62‐positive autophagosomes was also reduced following HA‐INPP4B^WT^ but not PI(3,4)P_2_ phosphatase‐dead HA‐INPP4B^C842A^ expression (Rijal *et al*, 2015; Appendix Fig S1G and H), indicating that INPP4B decreases autophagosome numbers via its conversion of PI(3,4)P_2_ to PI(3)P.

The INPP4B‐mediated decrease in autophagosomes observed during basal autophagy may be a consequence of either reduced autophagosome biogenesis, or enhanced autophagosome turnover via their fusion with lysosomes to form autolysosomes (Mizushima & Yoshimori, 2007). To distinguish between these two possibilities, cells were treated with the V‐ATPase inhibitor bafilomycin A1, which prevents autophagosome–lysosome fusion (Mauvezin *et al*, 2015). Bafilomycin A1 treatment restored both the decreased LC3B‐II levels and p62‐positive autophagosomes in GFP‐INPP4B expressing cells to a level similar to GFP‐vector controls (Fig 1H–K). This data suggests that INPP4B enhances autophagosome turnover and hence autophagic degradation but does not affect autophagosome formation. To evaluate autophagosome fusion with lysosomes, we utilized the pH‐sensitive GFP‐mCherry‐LC3 biosensor, which is detected as GFP‐positive/mCherry‐positive when at autophagosomes and GFP‐negative/mCherry‐positive at autolysosomes. This analysis revealed that *INPP4B* shRNA depletion increased LC3 biosensor association at autophagosomes, coupled with a decrease at autolysosomes, in growth media but not EBSS‐treated cells (Appendix Fig S1I–K). Taken together this data are consistent with an interpretation that INPP4B is required for basal autophagic degradation.

Growth factor stimulation activates PI3Kα signaling, which generates PI(3,4,5)P_3_ and subsequently PI(3,4)P_2_ that is hydrolyzed by INPP4B to PI(3)P (Rodgers *et al*, 2021). As INPP4B promotes autophagosome turnover during nutrient‐rich but not starvation conditions, we predicted that INPP4B regulation of basal autophagy requires PI3Kα signaling. Treatment with the PI3Kα inhibitor BYL719, which suppressed AKT/mTOR signaling, had little effect on LC3B levels in GFP‐vector cells but significantly increased the reduced LC3B‐II in GFP‐INPP4B cells (Fig 1L and M), suggesting that INPP4B‐generated PI(3)P promotes autophagosome turnover downstream of PI3Kα. PI(3)P is also generated on omegasomes by Vps34, stimulating autophagosome formation during starvation‐induced autophagy by recruiting the PI(3)P effector WIPI2 (Axe *et al*, 2008; Polson *et al*, 2010; Russell *et al*, 2013; Dooley *et al*, 2014). However, our data indicates INPP4B‐generated PI(3)P did not affect autophagosome formation, and had no effect on the number of WIPI2‐positive omegasomes under growth or starvation conditions (Appendix Fig S1L and M). Additionally, treatment with the Vps34 inhibitor SAR405, which suppresses autophagosome formation during starvation‐induced autophagy (Ronan *et al*, 2014), had no further effect on the reduced LC3B‐II levels in GFP‐INPP4B cells under basal autophagy conditions (Fig 1N and O). Altogether, our findings are consistent with a model whereby INPP4B‐generated PI(3)P promotes PI3Kα‐dependent basal autophagic degradation independent of Vps34‐generated PI(3)P.

### INPP4B‐generated PI(3)P on late endosomes is retained on endolysosomes

**Figure 2:**
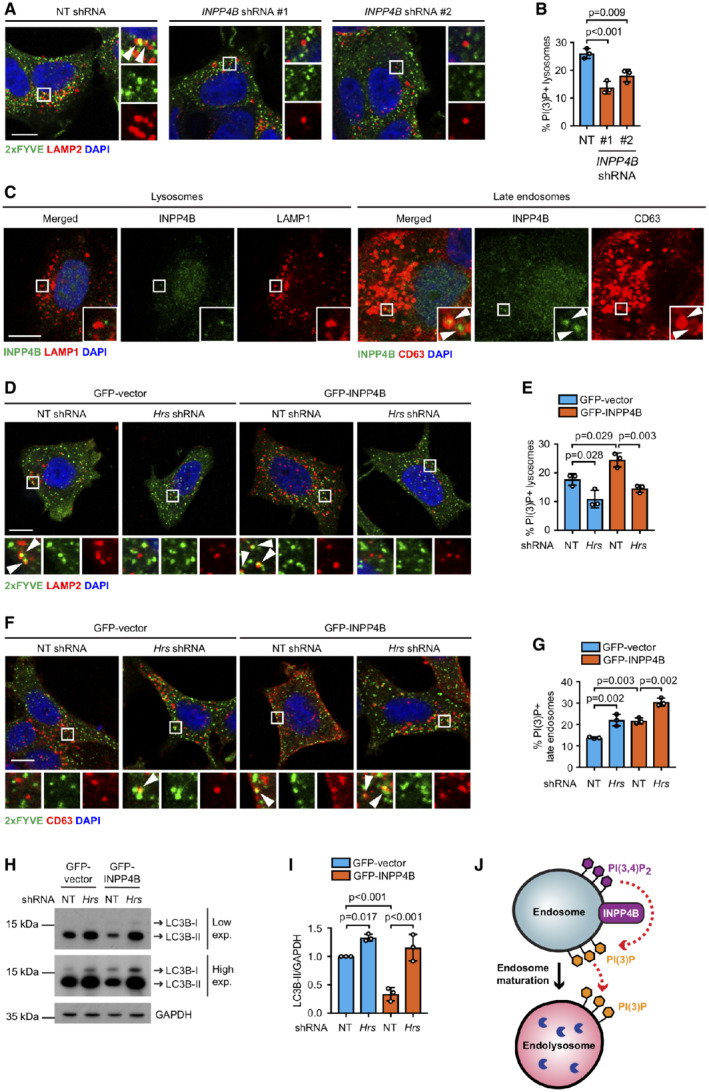
INPP4B‐generated PI(3)P on late endosomes is retained on endolysosomes A, B
MCF‐7 cells expressing nontargeted (NT), *INPP4B* #1, or *INPP4B* #2 shRNA were fixed and immunostained with recombinant GST‐2xFYVE^Hrs^ (2xFYVE) (PI(3)P probe) and LAMP2 antibodies, and co‐stained with DAPI (A). Data represent the proportion of PI(3)P^+^ lysosomes (*n* = 3 experiments, > 40 cells/experiment) (B). Arrows indicate co‐localization between 2xFYVE and LAMP2.C
MCF‐7 cells were treated with 0.02% (w/v) saponin for 30 s, then fixed and immunostained with INPP4B and either LAMP1 or CD63 antibodies, and co‐stained with DAPI. Arrows indicate co‐localization between INPP4B and CD63.D, E
MCF‐7 cells co‐expressing GFP‐INPP4B or GFP‐vector, and *Hrs* or NT shRNA, were fixed and immunostained with recombinant 2xFYVE (PI(3)P probe) and LAMP2 antibodies, and co‐stained with DAPI (D). Data represent the proportion of PI(3)P^+^ lysosomes (*n* = 3 experiments, > 30 cells/experiment) (E). Arrows indicate co‐localization between 2xFYVE and LAMP2.F, G
MCF‐7 cells co‐expressing GFP‐INPP4B or GFP‐vector, and *Hrs* or NT shRNA, were fixed and immunostained with recombinant 2xFYVE (PI(3)P probe) and CD63 antibodies, and co‐stained with DAPI (F). Data represent the proportion of PI(3)P^+^ late endosomes (*n* = 3 experiments, > 30 cells/experiment) (G). Arrows indicate co‐localization between 2xFYVE and LAMP2.H, I
MCF‐7 cells co‐expressing GFP‐INPP4B or GFP‐vector, and *Hrs* or NT shRNA, were lysed and immunoblotted with LC3B antibodies, and GAPDH antibodies as a loading control (H). Data represent the relative LC3B‐II levels normalized to GAPDH, and expressed relative to GFP‐vector; NT shRNA cells which were assigned an arbitrary value of 1 (*n* = 3 experiments) (I).J
INPP4B generates PI(3)P on late endosomes, which is retained on endolysosomes following late endosome maturation. Data information: Data are presented as mean ± SD. The insets at the lower right or bottom of each image are higher power regions of the boxed areas. Scale bar is 10 μm in (A, C, D, F). *P* values determined by one‐way ANOVA with Tukey *post hoc* test in (B, E, G), or by one‐way ANOVA in (I). Source data are available online for this figure.

**Figure EV1:**
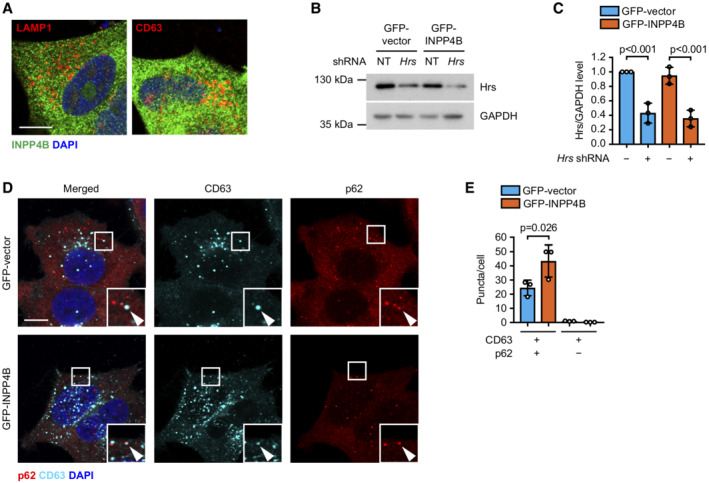
INPP4B does not affect amphisome formation A
MCF‐7 cells were fixed and immunostained with INPP4B and either LAMP1 or CD63 antibodies, and co‐stained with DAPI.B, C
MCF‐7 cells expressing GFP‐INPP4B or GFP‐vector were transduced with lentiviral particles encoding nontargeted (NT) or *Hrs* shRNA. Cells were lysed and immunoblotted with Hrs antibodies, and GAPDH antibodies as a loading control (B). Data represent the relative Hrs levels normalized to GAPDH expression, and expressed relative to GFP‐vector;NT shRNA cells which were assigned an arbitrary value of 1 (*n* = 3 experiments) (C).D, E
MCF‐7 cells expressing GFP‐INPP4B or GFP‐vector were fixed and stained with CD63 and p62 antibodies, and co‐stained with DAPI (D). Data represent the number of late endosomes (CD63^+^/p62^−^ puncta) and amphisomes (CD63^+^/p62^+^ puncta) per cell (*n* = 3 experiments, > 50 cells/experiment) (E). Arrows indicate amphisomes. Data information: Data are presented as mean ± SD. The insets at the lower right of each image are higher power regions of the boxed areas. Scale bar is 10 μm in (A, D). *P* values determined by one‐way ANOVA with Tukey *post hoc* test in (C, E). Source data are available online for this figure.

To determine how INPP4B promotes basal autophagy, the intracellular site of INPP4B‐mediated PI(3)P generation was analyzed. INPP4B exhibits a diffusely cytosolic distribution, but has also been identified on early and late endosomes where it converts PI(3,4)P_2_ to PI(3)P downstream of class I PI3K signaling (Li Chew *et al*, 2015; Liu *et al*, 2018; Rodgers *et al*, 2021). Interestingly, both PI(3,4)P_2_ and PI(3)P have been identified on lysosomes (Munson *et al*, 2015; Marat *et al*, 2017). Autophagosomes accumulate if lysosome function is impaired (McGrath *et al*, 2021). Therefore, we predicted that the deficit observed in autophagosome turnover with INPP4B depletion may be a consequence of an inability to regulate PI(3,4)P_2_ to PI(3)P conversion on lysosomes and thereby sustain lysosome homeostasis. To examine this possibility, PI(3)P was assessed under growth conditions using the 2xFYVE recombinant PI(3)P biosensor (Gillooly *et al*, 2000). *INPP4B* shRNA depletion significantly reduced the proportion of PI(3)P‐positive lysosomes (Fig 2A and B), suggesting that INPP4B contributes to PI(3)P generation on this compartment. To assess whether INPP4B itself localizes to lysosomes, cells were pretreated with saponin in order to remove cytoplasmic and retain intracellular membrane‐associated proteins (Figs 2C and EV1A). Extensive INPP4B co‐localization with CD63‐positive late endosomes was observed under these conditions, as previously reported (Rodgers *et al*, 2021), but surprisingly no co‐localization between INPP4B and LAMP1‐positive lysosomes was apparent. These findings indicate INPP4B contributes to PI(3)P generation on lysosomes, but does not itself localize to this compartment.

We recently reported that INPP4B promotes late endosome/lysosome formation via the PI(3)P‐binding endosomal sorting complex required for transport (ESCRT) protein, Hrs (Rodgers *et al*, 2021). ESCRT regulates the formation of intraluminal vesicles (ILVs) to promote maturation of late endosomes, which is in turn required for the subsequent fusion of late endosomes with lysosomes to form endolysosomes that degrade endocytic cargo (Bache *et al*, 2006; Malerød *et al*, 2007; Urwin *et al*, 2010). Interestingly, ESCRT inactivation also leads to autophagosome accumulation by unknown mechanisms (Filimonenko *et al*, 2007; Rusten *et al*, 2007), reminiscent of the effects we observed with *INPP4B* depletion. We hypothesized that PI(3)P generated by INPP4B on late endosomes may be retained upon endosome maturation to endolysosomes contributing to autophagic degradation. Suppression of late endosome maturation via *Hrs* shRNA depletion (Fig EV1B and C) significantly reduced PI(3)P‐positive lysosomes, and increased PI(3)P‐positive late endosomes, in both GFP‐INPP4B and vector control cells (Fig 2D–G). This data suggests that PI(3)P generated by INPP4B on late endosomes persists on endolysosomes following late endosome maturation. Furthermore, *Hrs* shRNA depletion modestly increased LC3B‐II in GFP‐vector cells, and restored the reduced LC3B‐II observed in GFP‐INPP4B expressing cells under growth conditions (Fig 2H and I). Therefore, we propose that INPP4B‐derived PI(3)P on lysosomes is required for autophagic degradation, events blocked by preventing endosome maturation to endolysosomes (Fig 2J). Late endosomes can also fuse with autophagosomes in some cells to form amphisomes, a hybrid compartment that facilitates degradation of endocytic and autophagic cargo (Zhao *et al*, 2021). However, we observed very few CD63‐positive/p62‐positive amphisomes in control or GFP‐INPP4B expressing cells (Fig EV1D and E), suggesting no influence on autophagic flux via amphisome formation.

### INPP4B promotes PI3Kα‐dependent lysosome formation

**Figure 3:**
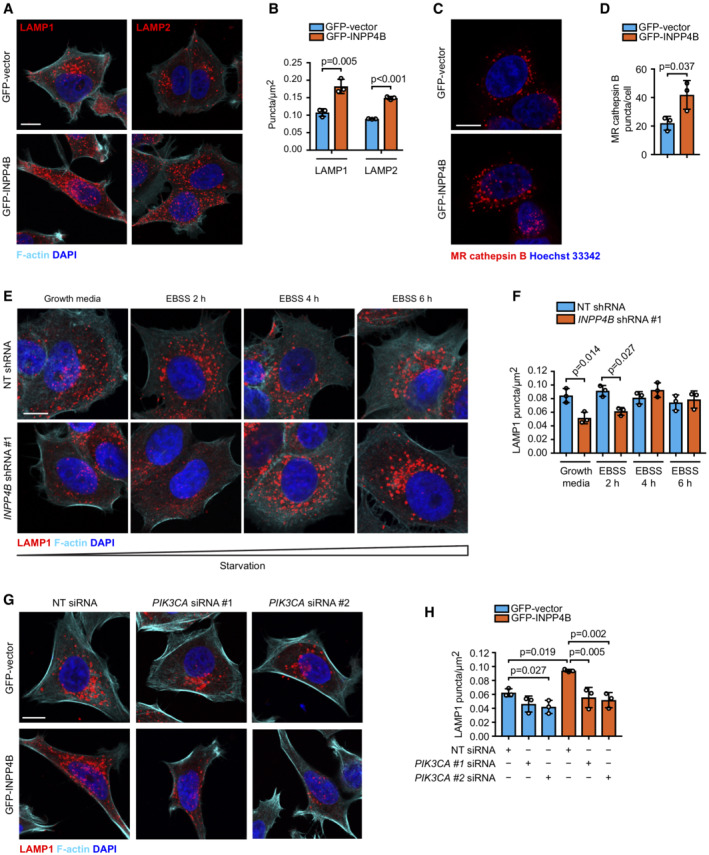
INPP4B promotes PI3Kα‐dependent lysosome formation A, B
MCF‐7 cells expressing GFP‐INPP4B or GFP‐vector were fixed and immunostained with LAMP1 or LAMP2 antibodies, and co‐stained with DAPI and phalloidin (A). Data represent the number of LAMP1^+^ or LAMP2^+^ puncta relative to cell area (μm^2^) (*n* = 3 experiments, > 50 cells/experiment) (B).C, D
Snapshots of Magic Red™ (MR) cathepsin B substrate and Hoechst 33342 captured in live MCF‐7 cells expressing GFP‐INPP4B or GFP‐vector (C). Data represent the number of MR cathepsin B^+^ puncta per cell (*n* = 3 experiments, > 50 cells/experiment) (D).E, F
MCF‐7 cells expressing nontargeted (NT) or *INPP4B* #1 shRNA were cultured in growth media or EBSS for the indicated times, then fixed and immunostained with LAMP1 antibodies and co‐stained with DAPI and phalloidin (E). Data represent the number of LAMP1^+^ puncta relative to cell area (μm^2^) (*n* = 3 experiments, > 30 cells per experiment) (F).G, H
MCF‐7 cells expressing GFP‐INPP4B or GFP‐vector were transfected with NT, *PIK3CA* #1, or *PIK3CA* #2 siRNA. After 24 h, cells were fixed and immunostained with LAMP1 antibodies, and co‐stained with DAPI and phalloidin (G). Data represent the number of LAMP1^+^ puncta relative to cell area (μm^2^) (*n* = 3 experiments, > 40 cells per experiment) (H). Data information: Data are presented as mean ± SD. Scale bar is 10 μm in (A, C, E, G). *P* values determined by two‐tailed unpaired *t* test in (B, D), by one‐way ANOVA with Tukey *post hoc* test in (F), or by one‐way ANOVA in (H). Source data are available online for this figure.

**Figure EV2:**
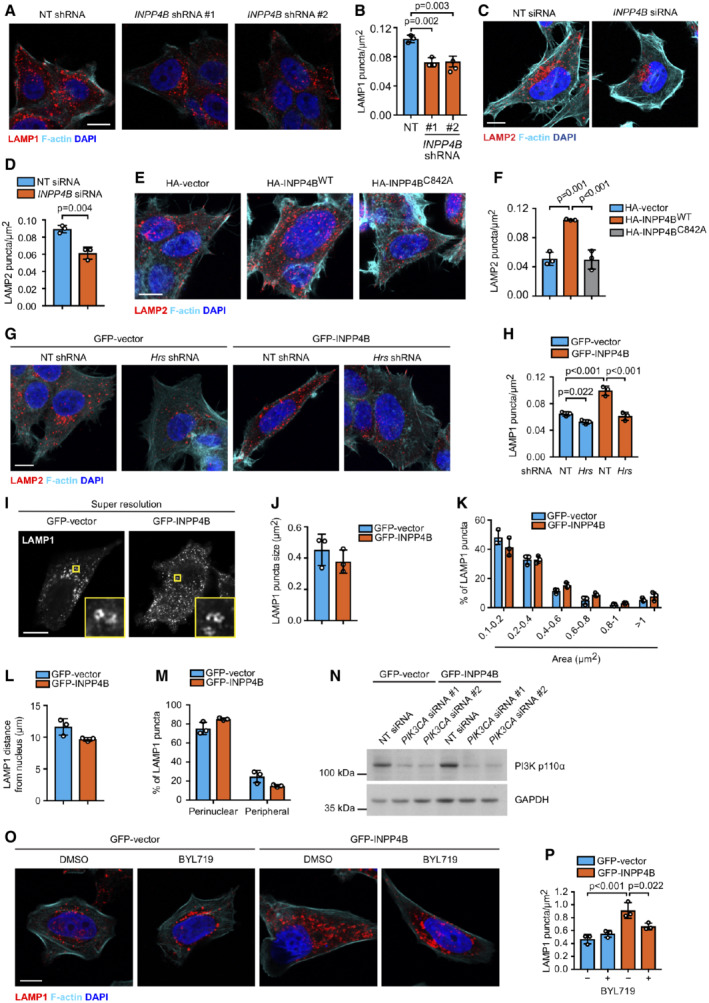
INPP4B does not affect lysosome size or dispersion A, B
MCF‐7 cells expressing nontargeted (NT), *INPP4B* #1, or *INPP4B* #2 shRNA were fixed and immunostained with LAMP1 antibodies, and co‐stained with DAPI and phalloidin (A). Data represent the number of LAMP1^+^ puncta relative to cell area (μm^2^) (*n* = 3 experiments, > 50 cells/experiment) (B).C, D
HeLa cells were transfected with NT or *INPP4B* siRNA. After 24 h, cells were fixed and immunostained with LAMP2 antibodies, and co‐stained with DAPI and phalloidin (C). Data represent the number of LAMP2^+^ puncta relative to cell area (μm^2^) (*n* = 3 experiments, > 50 cells/experiment) (D).E, F
MCF‐7 cells were transfected with HA‐vector, HA‐INPP4B,^WT^ or HA‐INPP4B^C842A^. Forty‐eight hours later, cells were fixed and immunostained with LAMP2 antibodies, and co‐stained with DAPI and phalloidin (E). Data represent the number of LAMP2^+^ puncta relative to cell area (μm^2^) (*n* = 3 experiments, > 20 cells/experiment) (F).G, H
MCF‐7 cells co‐expressing GFP‐INPP4B or GFP‐vector, and *Hrs* or NT shRNA, were fixed and immunostained with LAMP1 antibodies, and co‐stained with DAPI and phalloidin (G). Data represent the number of LAMP1^+^ puncta relative to cell area (μm^2^) (*n* = 3 experiments, > 40 cells per experiment) (H).I–K
MCF‐7 cells expressing GFP‐INPP4B or GFP‐vector were fixed and immunostained with LAMP1 antibodies, and imaged using super resolution microscopy (I). Data represent the LAMP1^+^ puncta size (J) and LAMP1^+^ puncta size distribution (K) (*n* = 3 experiments, > 20 cells/experiment).L, M
Data represent the distance of LAMP1^+^ puncta from the center of the nucleus (L), and the proportion of perinuclear LAMP1^+^ puncta (< 15 μm from center of nucleus) and peripheral LAMP1^+^ puncta (> 15 μm from center of nucleus) (M) (*n* = 3 experiments, > 20 cells/experiment).N
MCF‐7 cells expressing GFP‐INPP4B or GFP‐vector were transfected with NT, *PIK3CA* #1, or *PIK3CA* #2 siRNA. After 24 h, cells were lysed and immunoblotted with PI3K p110α antibodies, and GAPDH antibodies as a loading control.O, P
MCF‐7 cells expressing GFP‐INPP4B or GFP‐vector were treated with 2 μM BYL719 (PI3Kα inhibitor) or DMSO as a vehicle control for 24 h, then fixed and immunostained with LAMP1 antibodies, and co‐stained with DAPI and phalloidin (O). Data represent the number of LAMP1^+^ puncta relative to cell area (μm^2^) (*n* = 3 experiments, > 50 cells per experiment) (P). Data information: Data are presented as mean ± SD. The insets at the bottom of each image are higher power regions of the boxed areas. Scale bar is 10 μm in (A, C, E, G, I, O). *P* values determined by one‐way ANOVA with Tukey *post hoc* test in (B, F, P), by two‐tailed unpaired *t* test in (D), or by one‐way ANOVA in (H). Source data are available online for this figure.

As autophagosomes accumulate with INPP4B depletion, together with a reduction of autophagosome–lysosome fusion, we questioned whether PI(3)P generated by INPP4B under basal autophagy conditions is required for lysosome homeostasis. Strikingly, GFP‐INPP4B significantly increased, whereas *INPP4B* shRNA or siRNA depleted, the number of LAMP1 and LAMP2‐positive lysosomes in MCF‐7 and HeLa cells (Figs 3A and B, and EV2A–D), suggesting that INPP4B regulates lysosome numbers in multiple cell types. Expression of HA‐INPP4B^WT^, but not PI(3,4)P_2_ phosphatase‐dead HA‐INPP4B^C842A^, also increased the number of LAMP2‐positive lysosomes in MCF‐7 cells (Fig EV2E and F), consistent with a requirement for INPP4B‐mediated PI(3,4)P_2_ to PI(3)P conversion. Critically, inhibition of late endosome maturation via *Hrs* shRNA depletion decreased INPP4B‐mediated enhanced lysosome numbers (Fig EV2G and H). INPP4B also enhanced lysosomal activity as assessed by Magic Red™ cathepsin B staining (Fig 3C and D). However, we did not observe any overt changes to lysosome size or positioning in cells with INPP4B overexpression or shRNA depletion, which we confirmed by super resolution microscopy imaging and analysis (Fig EV2I–M). Collectively, our results reveal that INPP4B‐generated PI(3)P enhances lysosome formation, a function reliant on the maturation of endosomes to endolysosomes.

As INPP4B enhanced autophagic degradation under growth conditions but not following nutrient deprivation, we examined whether INPP4B regulation of lysosome formation was also restricted to growth conditions. Interestingly, although *INPP4B* shRNA depletion reduced lysosome numbers under growth conditions, this had little effect on lysosome numbers following prolonged nutrient deprivation (Fig 3E and F), revealing INPP4B regulation of lysosome formation and autophagic flux requires high nutrient availability. PI3Kα is a heterodimer consisting of a catalytic p110α subunit and a p85 regulatory subunit that is activated by growth factor stimulation. To investigate dependence on PI3Kα, we performed siRNA depletion of *PIK3CA*, which encodes the p110α catalytic subunit of PI3Kα, or treated cells with the PI3Kα inhibitor BYL719 (Fig EV2N). *PIK3CA* depletion or BYL719 treatment significantly reduced the increased number of lysosomes in GFP‐INPP4B cells under nutrient‐rich conditions (Figs 3G and H, and EV2O and P). Therefore, we propose that INPP4B‐generated PI(3)P promotes lysosome formation downstream of PI3Kα signaling under nutrient‐rich conditions, leading to enhanced basal autophagic flux.

### INPP4B enhances lysosome reformation from endolysosomes

**Figure EV3:**
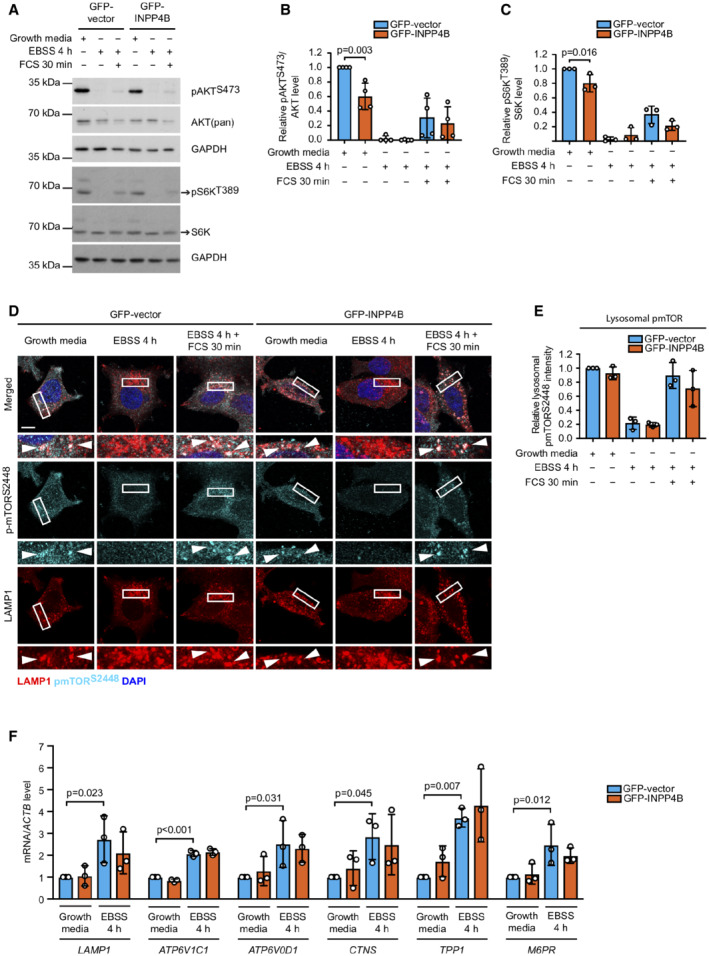
INPP4B does not regulate mTOR‐dependent lysosome biogenesis A–C
MCF‐7 cells expressing GFP‐INPP4B or GFP‐vector were cultured in growth media, EBSS (4 h), or EBSS (4 h) followed by 10% FCS (30 min). Cells were lysed and immunblotted with pAKT^S473^, AKT(pan), pS6K^T389,^ and S6K antibodies, and GAPDH antibodies as a loading control (A). Data represent the relative pAKT^S473^ levels normalized to AKT(pan) (B) or pS6K^T389^ levels normalized to S6K (C), and expressed relative to growth media‐treated GFP‐vector cells which were assigned an arbitrary value of 1 (*n* = 3 experiments).D, E
MCF‐7 cells expressing GFP‐INPP4B or GFP‐vector were cultured in growth media, EBSS (4 h) or EBSS (4 h) followed by 10% FCS (30 min). Cells were fixed and immunostained with pmTOR^S2448^ and LAMP1 antibodies, and co‐stained with DAPI (D). Data represent mean pmTOR^S2448^ fluorescence intensity overlapping with LAMP1^+^ puncta expressed relative to growth media‐treated GFP‐vector cells which were assigned an arbitrary value of 1 (*n* = 3 experiments) (E).F
MCF‐7 cells expressing GFP‐INPP4B or GFP‐vector were cultured in growth media or EBSS for 4 h. RNA was extracted and two‐step quantitative RT‐PCR was performed using primers for *LAMP1*, *ATP6V1C1*, *ATP6V0D1*, *CTNS*, *TPP1*, or *M6PR*, and expression was normalized to *ACTB*. Expression was determined using the ΔΔCt method and expressed relative to growth media‐treated GFP‐vector cells, which were assigned an arbitrary value of 1 (*n* = 3 experiments). Data information: Data are presented as mean ± SD. The insets at the bottom of each image are higher power regions of the boxed areas. Scale bar is 10 μm in (D). *P* values determined by two‐way ANOVA in (B, C, F). Source data are available online for this figure.

To determine how INPP4B‐generated PI(3)P promotes lysosome formation, we investigated whether INPP4B regulates known lysosome biogenesis pathways. Inactivation of lysosome‐associated mTOR by nutrient depletion promotes TFEB/TFE3 nuclear translocation and lysosomal gene transcription, leading to *de novo* lysosome biogenesis (Settembre *et al*, 2011, 2012). PI(3,4)P_2_ is required for activation of AKT/mTOR signaling (Gewinner *et al*, 2009; Fedele *et al*, 2010), whereas PI(3)P is required for localized mTOR activation on lysosomes (Nobukuni *et al*, 2005; Hong *et al*, 2017). We therefore examined whether INPP4B regulates lysosome biogenesis via mTOR regulation, which was assessed basally under growth media conditions or with EBSS ± serum stimulation to suppress and reactivate mTOR, respectively. INPP4B modestly suppressed phosphorylation‐dependent activation of AKT^S473^ and the mTOR substrate S6K^T389^ under growth conditions (Fig EV3A–C) as previously reported (Gewinner *et al*, 2009; Fedele *et al*, 2010), but had no effect on localized mTOR^S2448^ phosphorylation on lysosomes (Fig EV3D and E). Furthermore, INPP4B did not alter the expression of TFEB/TFE3 target genes, including *LAMP1*, *ATP6V1C1*, *ATP6VOD1*, *CTNS*, *TPP1*, or *M6PR*, under growth or starvation conditions (Fig EV3F). Taken together, these findings indicate that although INPP4B modestly suppresses AKT and mTOR activation, this is insufficient to stimulate *de novo* lysosome biogenesis.

**Figure 4:**
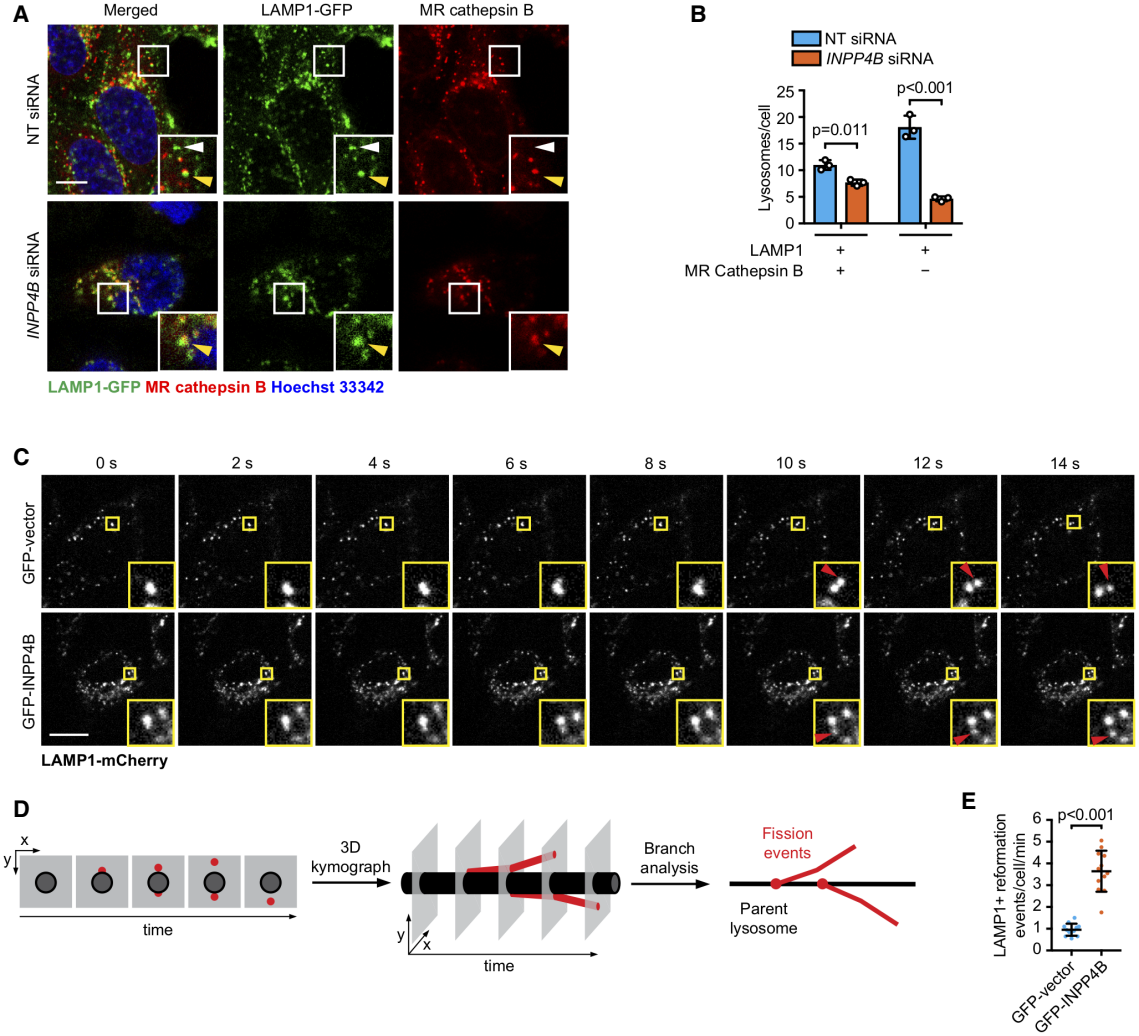
INPP4B promotes lysosome reformation A, B
Snapshots of LAMP1‐GFP, Magic Red™ (MR) cathepsin B substrate, and Hoechst 33342 captured in live HeLa cells transfected with *INPP4B* or nontargeted siRNA (A). Data represent the number of LAMP1^+^/MR cathepsin B^+^ and LAMP1^+^/MR cathepsin B^−^ lysosomes (terminal storage lysosomes) per cell (*n* = 3 experiments, > 50 cells/experiment) (B). Yellow arrows indicate LAMP1^+^/MR cathepsin B^+^ lysosomes, and white arrows indicate terminal storage lysosomes.C
Timelapse snapshots of LAMP1‐mCherry signals from MCF‐7 cells expressing GFP‐INPP4B or GFP‐vector captured by spinning disk microscopy. Maximum intensity projections of three z‐planes taken 0.27 μm apart are shown. Arrows indicate lysosome reformation.D
Overview of analysis workflow used to quantify lysosome reformation from spinning disk microscopy. Maximum intensity projections of lysosomes were converted to three‐dimensional kymographs using *x*, *y*, and time dimensions, then segmented and skeletonized to track tubulation and fission events (depicted in red) that branch off the parent lysosome (depicted in black).E
Data represent the number of LAMP1^+^ reformation events per cell per minute (*n* = 12 GFP‐vector cells, *n* = 15 GFP‐INPP4B cells). Data information: Data are presented as mean ± SD. The insets at the lower right of each image are higher power regions of the boxed areas. Scale bar is 10 μm in (A, C). *P* values determined by two‐way ANOVA with Holm–Sidak *post‐hoc* test in (B), or by two‐tailed unpaired *t* test in (E). Source data are available online for this figure.

An alternate mechanism for lysosome generation is via lysosome reformation pathways that regenerate new lysosomes from existing autolysosomal or endolysosomal membranes under specific cellular conditions. ALR mediates lysosome repopulation during prolonged starvation‐induced autophagy but this process is inactive under growth conditions (Yu *et al*, 2010). Instead, lysosomes are generated by tubulation and scission of endolysosome membranes via a process known as lysosome reformation from endolysosomes (Pryor *et al*, 2000; Bright *et al*, 2005). However, it has not been reported whether lysosome reformation from endolysosomes affects autophagy. Lysosomes that are newly formed via reformation from endolysosomes are called terminal storage lysosomes, which do not contain active hydrolases and can be distinguished from endolysosomes, which contain active hydrolases (Bright *et al*, 2016). Interestingly, *INPP4B* siRNA depletion significantly reduced the number of terminal storage lysosomes (LAMP1‐positive, Magic Red™ cathepsin B‐negative; Fig 4A and B). We therefore questioned whether INPP4B‐mediated PI(3,4)P_2_ to PI(3)P conversion contributes to lysosome reformation from endolysosomes. Quantitative analysis of lysosome reformation events has been a significant challenge as the timing of endolysosome tubule budding and separation occurs within several seconds (Bissig *et al*, 2017). To address this, we developed a rapid imaging and analysis workflow to identify and quantify lysosome reformation events in live cells. This analysis used spinning disk microscopy of live MCF‐7 cells expressing GFP‐INPP4B or GFP‐vector to capture reformation of LAMP1‐mCherry‐positive lysosomes under growth conditions (Fig 4C, Movies EV1 and EV2). Two distinct lysosome populations were observed; larger, slower moving lysosomes that underwent reformation, and smaller, faster moving lysosomes that did not appear to undergo reformation, although these lysosomes were difficult to individually track across the entire time‐lapse as they frequently moved outside the focal plane. To quantify the relative rate of lysosome reformation, kymographs of the larger, slow‐moving lysosomes were constructed and converted to “skeletons” to identify branch points corresponding to lysosomal tubulation and fission events (Fig 4D and Appendix Fig S2A–E). Strikingly, this analysis revealed an increased number of lysosome reformation events per minute in GFP‐INPP4B compared to vector control cells (Fig 4E), suggesting that INPP4B enhances lysosome numbers by increasing the rate of lysosome reformation.

### INPP4B‐generated PI(3)P is phosphorylated to PI(3,5)P_2_ by PIKfyve to promote lysosome reformation

**Figure 5:**
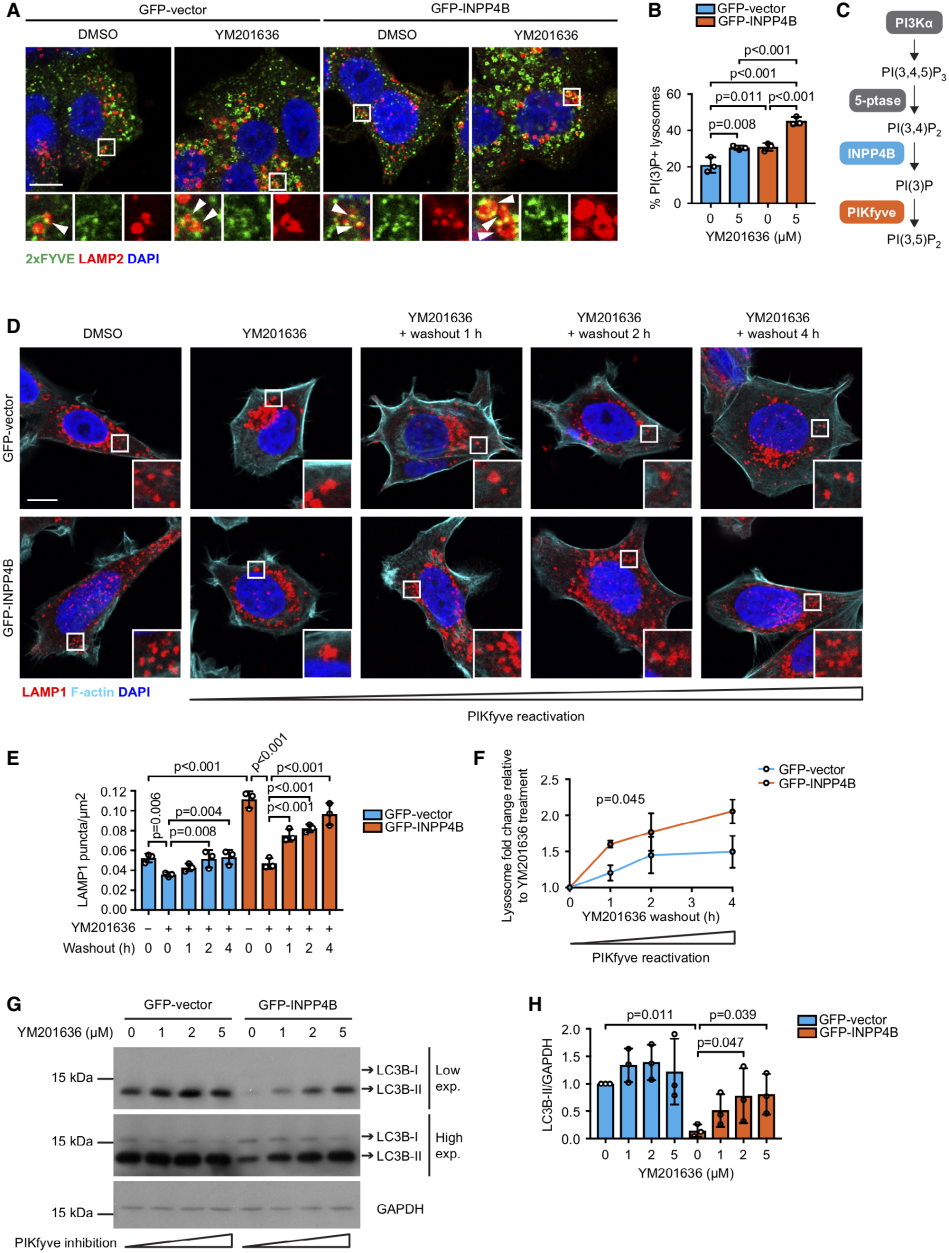
INPP4B promotes PIKfyve‐dependent lysosome reformation and autophagic flux A, B
MCF‐7 cells expressing GFP‐INPP4B or GFP‐vector were treated with 5 μM YM201636 (PIKfyve inhibitor) or DMSO as a vehicle control for 2 h. Cells were fixed and immunostained with recombinant GST‐2xFYVE^Hrs^ (2xFYVE) (PI(3)P probe) and LAMP2 antibodies, and co‐stained with DAPI (A). Data represent the proportion of PI(3)P^+^ lysosomes (*n* = 3 experiments, > 30 cells/experiment) (B). Arrows show co‐localization between 2xFYVE and LAMP2.C
INPP4B generates PI(3)P downstream of PI3Kα signaling, which is predicted to act as a substrate for phosphorylation to PI(3,5)P_2_ by PIKfyve.D–F
MCF‐7 cells expressing GFP‐INPP4B or GFP‐vector were treated with 5 μM YM201636 (PIKfyve inhibitor) or DMSO as a vehicle control for 2 h, then YM201636 was washed out for 1, 2, or 4 h. Cells were fixed and immunostained with LAMP1 antibodies, and co‐stained with DAPI and phalloidin (D). Data represent the number of LAMP1^+^ puncta relative to cell area (μm^2^) (E), and the lysosome fold change relative to the YM201636‐treated condition for each cell line (F) (*n* = 3 experiments, > 30 cells per experiment).G, H
MCF‐7 cells expressing GFP‐INPP4B or GFP‐vector were treated with 1, 2, or 5 μM YM201636 (PIKfyve inhibitor) or DMSO as a vehicle control for 4 h. Cells were lysed and immunoblotted with LC3B antibodies, and GAPDH antibodies as a loading control (G). Data represent the relative LC3B‐II levels normalized to GAPDH, and expressed relative to DMSO‐treated GFP‐vector cells which were assigned an arbitrary value of 1 (*n* = 3 experiments) (H). Data information: Data are presented as mean ± SD. The insets at the lower right or bottom of each image are higher power regions of the boxed areas. Scale bar is 10 μm in (A, D). *P* values determined by one‐way ANOVA in (B, E, H), or by two‐tailed unpaired *t* test of the area under the curve in (F). Source data are available online for this figure.

The molecular pathway that directs lysosome reformation from endolysosomes is not well understood, however, several reports show a requirement for localized PI(3,5)P_2_ generation on endolysosomes (Bissig *et al*, 2017; Choy *et al*, 2018). All cellular PI(3,5)P_2_ is synthesized from PI(3)P by the PI(3)P 5‐kinase PIKfyve, in complex with the scaffold protein Vac14 and the lipid/protein phosphatase Fig4 (Zolov *et al*, 2012; Lees *et al*, 2020). PI(3,5)P_2_ depletion via PIKfyve inactivation suppresses lysosome reformation from endolysosomes leading to fewer and swollen lysosomes (Bissig *et al*, 2017; Choy *et al*, 2018). We hypothesized that INPP4B conversion of PI(3,4)P_2_ to PI(3)P, the latter being retained on endolysosomes, may provide a substrate for PIKfyve to generate PI(3,5)P_2_ that drives lysosome reformation. Currently available PI(3,5)P_2_ probes display poor selectivity (Hammond *et al*, 2015). Therefore, we imaged PI(3)P in the presence of the PIKfyve inhibitor, YM201636, as an indirect measure of PI(3)P conversion to PI(3,5)P_2_. YM201636 treatment increased the proportion of lysosomes with PI(3)P‐positive staining, revealing its conversion to PI(3,5)P_2_ was blocked. PI(3)P was further increased by concurrent GFP‐INPP4B expression (Fig 5A and B), indicating that INPP4B‐generated PI(3)P acts as a substrate for PIKfyve to form PI(3,5)P_2_ (Fig 5C).

**Figure EV4:**
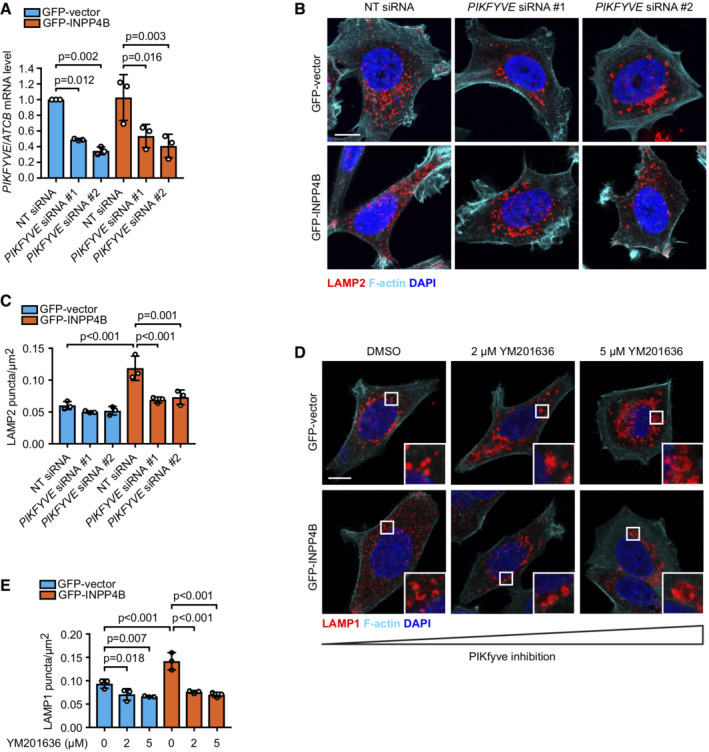
INPP4B promotes PIKfyve‐dependent lysosome reformation A
MCF‐7 cells expressing GFP‐INPP4B or GFP‐vector were transfected with nontargeted (NT), *PIKFYVE* #1, or *PIKFYVE* #2 siRNA. After 24 h, RNA was extracted and two‐step quantitative RT‐PCR was performed using primers for *PIKFYVE*, and expression was normalized to *ACTB*. Expression was determined using the ΔΔCt method and expressed relative to GFP‐vector;NT siRNA cells which were assigned an arbitrary value of 1 (*n* = 3 experiments).B, C
MCF‐7 cells expressing GFP‐INPP4B or GFP‐vector were transfected with NT, *PIKFYVE* #1, or *PIKFYVE* #2 siRNA. After 24 h, cells were fixed and immunostained with LAMP2 antibodies, and co‐stained with DAPI and phalloidin (B). Data represent the number of LAMP2^+^ puncta relative to cell area (μm^2^) (*n* = 3 experiments, > 40 cells per experiment) (C).D, E
MCF‐7 cells expressing GFP‐INPP4B or GFP‐vector were treated with 2 or 5 μM YM201636 (PIKfyve inhibitor) or DMSO as a vehicle control for 4 h. Cells were fixed and immunostained with LAMP1 antibodies, and co‐stained with DAPI and phalloidin (D). Data represent the number of LAMP1^+^ puncta relative to cell area (μm^2^) (*n* = 3 experiments, > 30 cells/experiment) (E). Data information: Data are presented as mean ± SD. The insets at the lower right of each image are higher power regions of the boxed areas. Scale bar is 10 μm in (B, D). *P* values determined by one‐way ANOVA with Tukey *post hoc* test in (A, C), or by one‐way ANOVA in (E). Source data are available online for this figure.

To determine whether INPP4B‐mediated lysosome reformation is dependent on PIKfyve conversion of PI(3)P to PI(3,5)P_2_, lysosome numbers were examined under conditions of PIKfyve inactivation. *PIKFYVE* siRNA depletion significantly reduced lysosome numbers in GFP‐INPP4B expressing cells, but had minimal effect on GFP‐vector controls, possibly as only a partial reduction in *PIKFYVE* mRNA expression was achieved (Fig EV4A–C). However, more robust effects were observed with YM201636 treatment, which resulted in significantly fewer and swollen lysosomes in both GFP‐INPP4B and GFP‐vector cells consistent with inhibition of lysosome reformation (Fig EV4D and E). As YM201636 inhibitor effects are reversible (Jefferies *et al*, 2008; Bissig *et al*, 2017), we also performed YM201636 washout experiments to assess if INPP4B enhances the rate of lysosome reformation under conditions of PIKfyve reactivation. Lysosome numbers were reduced in both GFP‐INPP4B and GFP‐vector cells with YM201636 treatment, and recovered after 4 h of YM201636 washout (Fig 5D and E). Notably, lysosome regeneration following PIKfyve reactivation occurred at a significantly faster rate in GFP‐INPP4B cells compared to vector controls (Fig 5F), consistent with the contention that INPP4B enhances lysosome reformation by supplying PI(3)P as a substrate for PIKfyve‐mediated PI(3,5)P_2_ generation. Furthermore, YM201636 treatment rescued the decreased LC3B‐II observed in GFP‐INPP4B cells (Fig 5G and H), suggesting that INPP4B promotes basal autophagic flux via enhanced PIKfyve‐dependent lysosome reformation.

**Figure 6:**
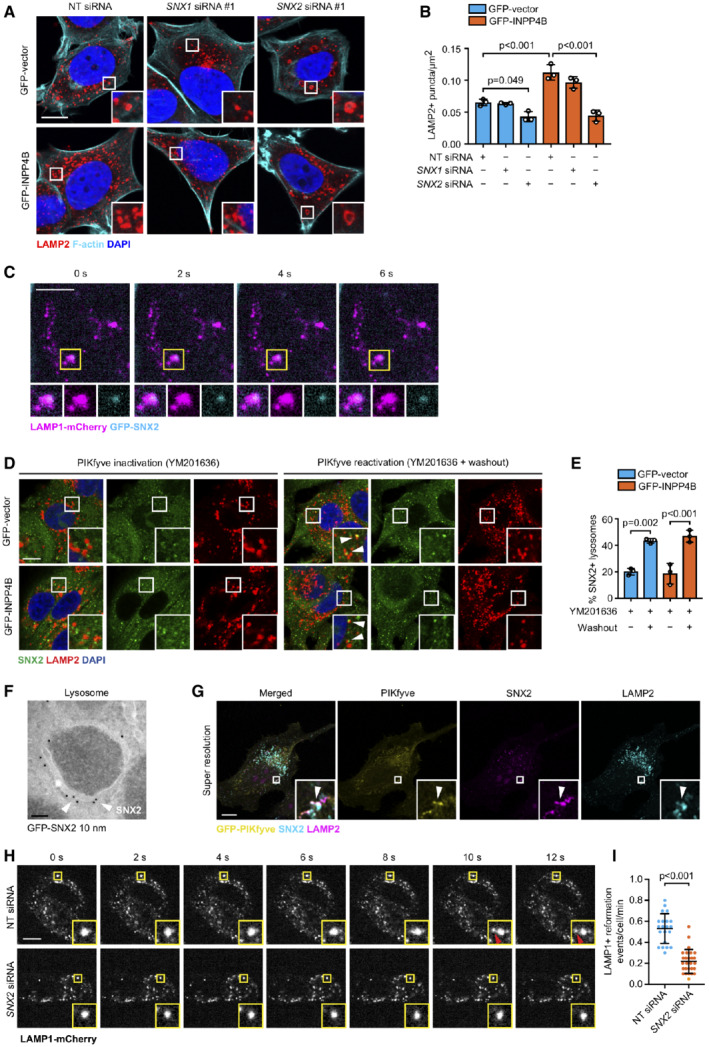
SNX2 is a putative PI(3,5)P_2_ effector that promotes lysosome reformation A, B
MCF‐7 cells expressing GFP‐INPP4B or GFP‐vector were transfected with nontargeted (NT), *SNX1*, or *SNX2* siRNA. After 24 h, cells were fixed and immunostained with LAMP2 antibodies, and co‐stained with DAPI and phalloidin (A). Data represent the number of LAMP2^+^ puncta relative to cell area (μm^2^; *n* = 3 experiments, > 40 cells per experiment) (B).C
Timelapse snapshots of LAMP1‐mCherry and GFP‐SNX2 signals from MCF‐7 cells captured by spinning disk microscopy. Maximum intensity projections of two z‐planes taken 0.27 μm apart are shown.D, E
MCF‐7 cells expressing GFP‐INPP4B or GFP‐vector were treated with 5 μM YM201636 (PIKfyve inhibitor) for 2 h, then YM201636 was washed out for 1 h. Cells were fixed and immunostained with SNX2 and LAMP1 antibodies, and co‐stained with DAPI (D). Data represent the proportion of SNX2^+^ LAMP2 puncta (*n* = 3 experiments, > 40 cells/experiment) (E). Arrows show co‐localization between SNX2 and LAMP2.F
HeLa cells were transfected with GFP‐SNX2. After 24 h, cells were treated with 5 μM YM201636 (PIKfyve inhibitor) for 2 h, then YM201636 was washed out for 1 h. Cells were fixed and subjected to immuno‐electron microscopy analysis using GFP (10 nm) antibodies. Arrows show GFP‐SNX2 localization on a lysosome.G
HeLa cells were transfected with GFP‐PIKfyve. After 24 h, cells were treated with 5 μM YM201636 (PIKfyve inhibitor) for 2 h, then YM201636 was washed out for 1 h. Cells were fixed and immunostained with SNX2, GFP, and LAMP2 antibodies, and imaged using super resolution microscopy. Arrows show co‐localization between GFP‐PIKfyve, SNX2, and LAMP2.H
MCF‐7 cells expressing LAMP1‐mCherry were transfected with NT or *SNX2* siRNA. After 24 h, cells were imaged by spinning disk microscopy for 20 min. Representative time‐lapse snapshots of LAMP1‐mCherry from a maximum projection of three z‐planes taken 0.27 μm apart are shown. Arrow indicates lysosome reformation.I
Data represent the number of LAMP1^+^ reformation events per cell per minute (*n* = 21 NT siRNA cells, *n* = 27 *SNX2* siRNA cells). Data information: Data are presented as mean ± SD. The insets at the lower right or bottom of each image are higher power regions of the boxed areas. Scale bar is 10 μm in (A, C, D, G, H), and 100 nm in (F). *P* values determined by one‐way ANOVA in with Tukey *post hoc* test in (B, E), or by two‐tailed unpaired *t* test in (I). Source data are available online for this figure.

### SNX2 is a PI(3,5)P_2_ effector that promotes lysosome reformation

The membrane‐associated molecular machinery that facilitates lysosome reformation from endolysosomes remains largely unknown. The PI(3,5)P_2_‐binding calcium channel protein, TRPML1, is the only described effector of lysosome reformation from endolysosomes, which is required for scission of endolysosome tubules (Miller *et al*, 2015). However, there are likely to be unidentified factors that co‐ordinate the initial budding and extension of endolysosome tubules. Interestingly, sorting nexin (SNX) proteins SNX1 and SNX2 contain a PI(3,5)P_2_‐binding phox homology (PX) domain, and a functional bin‐amphiphysin‐rvs (BAR) domain that detects and promotes membrane curvature (Cozier *et al*, 2002; Carlton *et al*, 2005). SNX1 and SNX2 form part of the retromer complex that regulates endosome‐to‐Golgi transport, but also possess *in vitro* membrane tubulation activity (van Weering *et al*, 2012), suggesting the potential to intrinsically regulate membrane deformation events. SNX1 and SNX2 were also identified in PI(3,5)P_2_ interactomes as well as Fig4 and Vac14 proximity interactomes (Catimel *et al*, 2008; Qiu *et al*, 2021), consistent with an association with the PIKfyve complex, but their function in this context has not been reported. We therefore investigated whether SNX1 or SNX2 function as PI(3,5)P_2_ effectors of lysosome reformation from endolysosomes. Lysosome numbers were assessed under growth conditions in GFP‐INPP4B or GFP‐vector cells with concurrent *SNX1* or *SNX2* siRNA depletion (Fig 6A and B, and Appendix Fig S3A and B). *SNX1* depletion had little impact on lysosomes. In contrast, *SNX2* depletion significantly reduced lysosome numbers in GFP‐INPP4B and GFP‐vector cells and lysosomes also appeared swollen, reminiscent of PIKfyve inhibition, suggesting that SNX2 is a potential effector of lysosome reformation. To exclude off‐target effects of *SNX2* siRNA, we also found that siRNAs targeting the 3'‐UTR region of SNX2 reduced lysosome numbers, and this effect was reversed by co‐expression of recombinant GFP‐SNX2 (Appendix Fig S3C–G).

SNX2 is predominantly recruited to early endosomes by PI(3)P where it regulates retromer‐dependent trafficking, but interestingly, SNX2 binds with similar affinity to PI(3,5)P_2_ and a minor pool of SNX2 localizes to lysosomes under growth conditions (Carlton *et al*, 2005; Mellado *et al*, 2014). Spinning disk microscopy revealed that GFP‐SNX2 co‐localized with LAMP1‐mCherry‐positive lysosomes undergoing reformation in live cells (Fig 6C). We determined whether SNX2 is recruited to lysosomes in response to PIKfyve‐generated PI(3,5)P_2_ using SNX2‐specific antibodies (Appendix Fig S3H and I). Co‐localization of endogenous SNX2 with lysosomes was minimal following PIKfyve inhibition in GFP‐INPP4B and GFP‐vector control cells, however, SNX2 lysosome co‐localization significantly increased following PIKfyve reactivation (Fig 6D and E), suggesting that SNX2 is recruited to lysosomes in a PI(3,5)P_2_‐dependent manner. This was confirmed by immuno‐electron microscopy, which demonstrated that a pool of GFP‐SNX2 localized to lysosome membranes during PIKfyve reactivation (Fig 6F). As current PI(3,5)P_2_ probes display poor selectivity (Hammond *et al*, 2015), we assessed SNX2 co‐localization with PIKfyve, the only enzyme that synthesizes PI(3,5)P_2_ (Zolov *et al*, 2012). Super resolution microscopy revealed that SNX2 co‐localized with GFP‐PIKfyve on lysosomes during PIKfyve reactivation (Fig 6G). Together, these findings suggest that SNX2 is recruited to lysosomes by PIKfyve generation of PI(3,5)P_2_. In control studies, we found that PIKfyve inhibition did not affect SNX2 localization to early endosomes (Appendix Fig S3J and K). To determine whether SNX2 is required for lysosome reformation, we utilized our analysis pipeline to identify and quantify lysosome reformation events in nontargeted (NT) and *SNX2* siRNA live MCF‐7 cells by spinning disk microscopy (Fig 6H, Movies EV3 and EV4). *SNX2* siRNA cells exhibited a striking decrease in the number of lysosome reformation events per minute compared to NT siRNA cells (Fig 6I). Notably, lysosome tubules were rarely detected in the absence of SNX2, suggesting that SNX2 may be required for the formation of endolysosome tubules during lysosome reformation. Taken together, these findings suggest that SNX2 is recruited to lysosomes by PIKfyve‐generated PI(3,5)P_2_ and contributes to lysosome reformation.

### INPP4B/PIKfyve‐dependent lysosome reformation protects against proteotoxic stress

**Figure 7:**
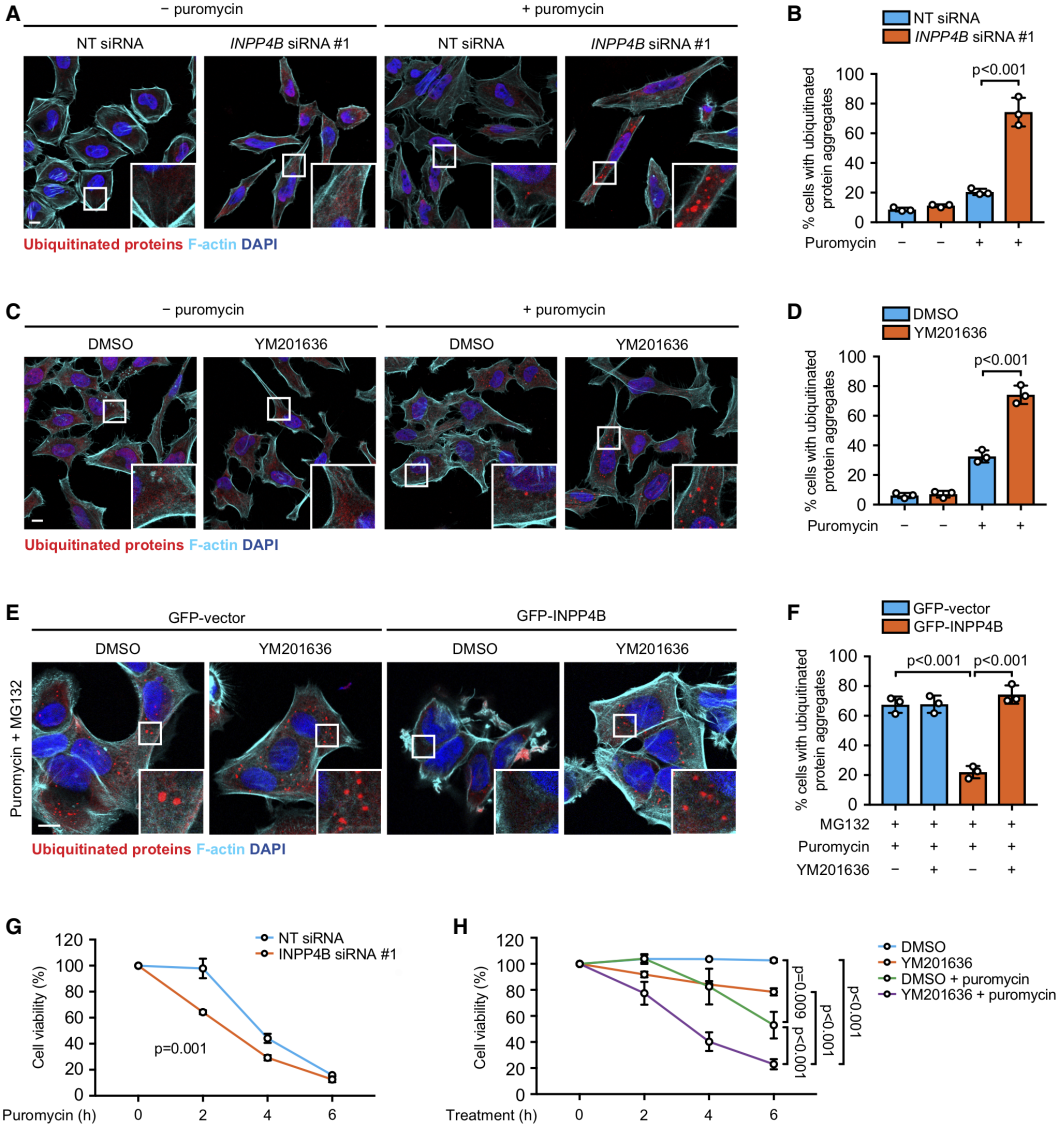
INPP4B and PIKfyve are required for proteotoxic stress response A, B
HeLa cells transfected with *INPP4B* or nontargeted (NT) siRNA were treated for 1 h with 5 μg/ml puromycin. Cells were fixed and immunostained with ubiquitin antibodies, and co‐stained with DAPI and phalloidin (A). Data represent the percentage of cells with ubiquitinated protein aggregates (*n* = 3 experiments, > 200 cells/experiment) (B).C, D
HeLa cells were treated with 5 μM YM201636 (PIKfyve inhibitor) or DMSO as a vehicle control for 2 h, then 5 μg/ml puromycin for 1 h. Cells were fixed and immunostained with ubiquitin antibodies, and co‐stained with DAPI and phalloidin (C). Data represent the percentage of cells with ubiquitinated protein aggregates (*n* = 3 experiments, > 200 cells/experiment) (D).E, F
MCF‐7 cells expressing GFP‐INPP4B or GFP‐vector were treated for 4 h with 10 μg/ml puromycin, 5 μm MG132, and either 5 μM YM201636 (PIKfyve inhibitor) or DMSO as a vehicle control. Cells were fixed and immunostained with ubiquitin antibodies, and co‐stained with DAPI and phalloidin (E). Data represent the percentage of cells with ubiquitinated protein aggregates (*n* = 3 experiments, > 200 cells/experiment) (F).G
HeLa cells transfected with *INPP4B* or NT siRNA were treated with 10 μg/ml puromycin for 2–6 h, then cell viability was assessed using CellTiter‐Glo® assays. Data represent the relative cell viability normalized to untreated cells (*n* = 3 experiments).H
HeLa cells were treated 10 μg/ml puromycin ±5 μM YM201636 (PIKfyve inhibitor) or DMSO as a vehicle control for 2–6 h, then cell viability was assessed using CellTiter‐Glo® assays. Data represent the relative cell viability normalized to untreated cells (*n* = 3 experiments). Data information: Data are presented as mean ± SD. The insets at the lower right or bottom of each image are higher power regions of the boxed areas. Scale bar is 10 μm in (A, C, E). *P* values determined by one‐way ANOVA in with Tukey *post hoc* test in (B, D, F), by two‐tailed unpaired *t* test of the area under the curve in (G), or by one‐way ANOVA with Tukey *post hoc* test of the area under the curve in (H). Source data are available online for this figure.

**Figure EV5:**
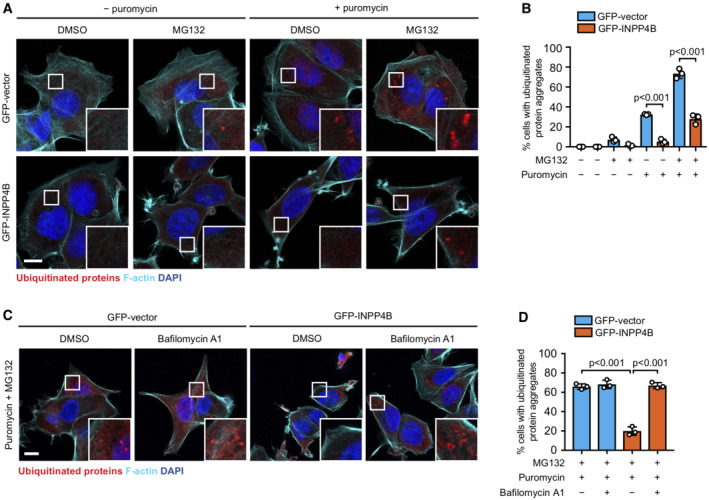
INPP4B promotes autophagic degradation of protein aggregates during proteotoxic stress A, B
MCF‐7 cells expressing GFP‐INPP4B or GFP‐vector were treated for 4 h with 10 μg/ml puromycin ±5 μm MG132 or DMSO as a vehicle control. Cells were fixed and immunostained with ubiquitin antibodies, and co‐stained with DAPI and phalloidin (A). Data represent the percentage of cells with ubiquitinated protein aggregates (*n* = 3 experiments, > 200 cells/experiment) (B).C, D
MCF‐7 cells expressing GFP‐INPP4B or GFP‐vector were treated for 4 h with 10 μg/ml of puromycin, 5 μm of MG132, and either 100 nM of bafilomycin A1 or DMSO as a vehicle control. Cells were fixed and immunostained with ubiquitin antibodies, and co‐stained with DAPI and phalloidin (C). Data represent the percentage of cells with ubiquitinated protein aggregates (n = 3 experiments, > 200 cells/experiment) (D). Data information: Data are presented as mean ± SD. The insets at the lower right or bottom of each image are higher power regions of the boxed areas. Scale bar is 10 μm in (A, C). *P* values determined by one‐way ANOVA in with Tukey *post hoc* test in (B, D). Source data are available online for this figure.

Basal autophagy is critically important for maintaining cellular homeostasis, and is required for a number of cellular processes including the protein quality control pathway. During proteotoxic stress, misfolded or aggregated proteins are tagged with ubiquitin chains and are subsequently degraded either by the ubiquitin proteasome system, or larger aggregates are sequestered by autophagosomes and degraded via autophagy. Prolonged proteotoxic stress activates apoptosis and cell death, and defective proteotoxic stress responses cause cytotoxicity leading to a range of pathological conditions (Dubnikov *et al*, 2017). Inhibition of basal autophagy *in vivo* following *Atg5* or *Atg7* deletion results in the accumulation of ubiquitinated protein aggregates leading to cytotoxicity and neurodegenerative disease (Hara *et al*, 2006; Komatsu *et al*, 2006), highlighting the critical cytoprotective role of basal autophagy. However, whether lysosome reformation from endolysosomes contributes to protein quality control remains unclear. We therefore investigated whether INPP4B/PIKfyve regulation of lysosome reformation and basal autophagy are required for protein aggregate clearance during proteotoxic stress. Puromycin is widely used experimentally to induce proteotoxic stress by prematurely terminating protein translation leading to misfolded proteins that accumulate as ubiquitin‐tagged aggregates (Fan *et al*, 2010; Park *et al*, 2017). *INPP4B* siRNA depletion or YM201636 treatment significantly increased the accumulation of protein aggregates in puromycin‐treated cells (Fig 7A–D). In contrast, GFP‐INPP4B reduced protein aggregation under similar experimental conditions (Fig EV5A and B). To exclude whether this difference was due to protein aggregate clearance by the ubiquitin proteasome system, cells were also co‐treated with the proteasome inhibitor MG132. GFP‐INPP4B expression also reduced protein aggregation under these conditions (Fig EV5A and B), which was reversed by concomitant YM201636 or bafilomycin A1 treatment (Figs 7E and F, and EV5C and D). Collectively, this data indicates that INPP4B/PIKfyve regulation of lysosome reformation is required for autophagic degradation of protein aggregates. Finally, we assessed whether INPP4B/PIKfyve‐dependent lysosome reformation protects against cytotoxicity during prolonged proteotoxic stress. Critically, we found that *INPP4B* siRNA depletion or YM201636 treatment significantly reduced cell viability in response to puromycin treatment (Fig 7G and H). These findings suggest that INPP4B/PIKfyve‐dependent lysosome reformation is required for protein quality control, and inactivation of this pathway leads to cell death. Altogether, our findings identify a phosphoinositide conversion pathway via endosomes that is required for lysosome reformation from endolysosomes, basal autophagic flux and protection against proteotoxic stress.

**Figure 8:**
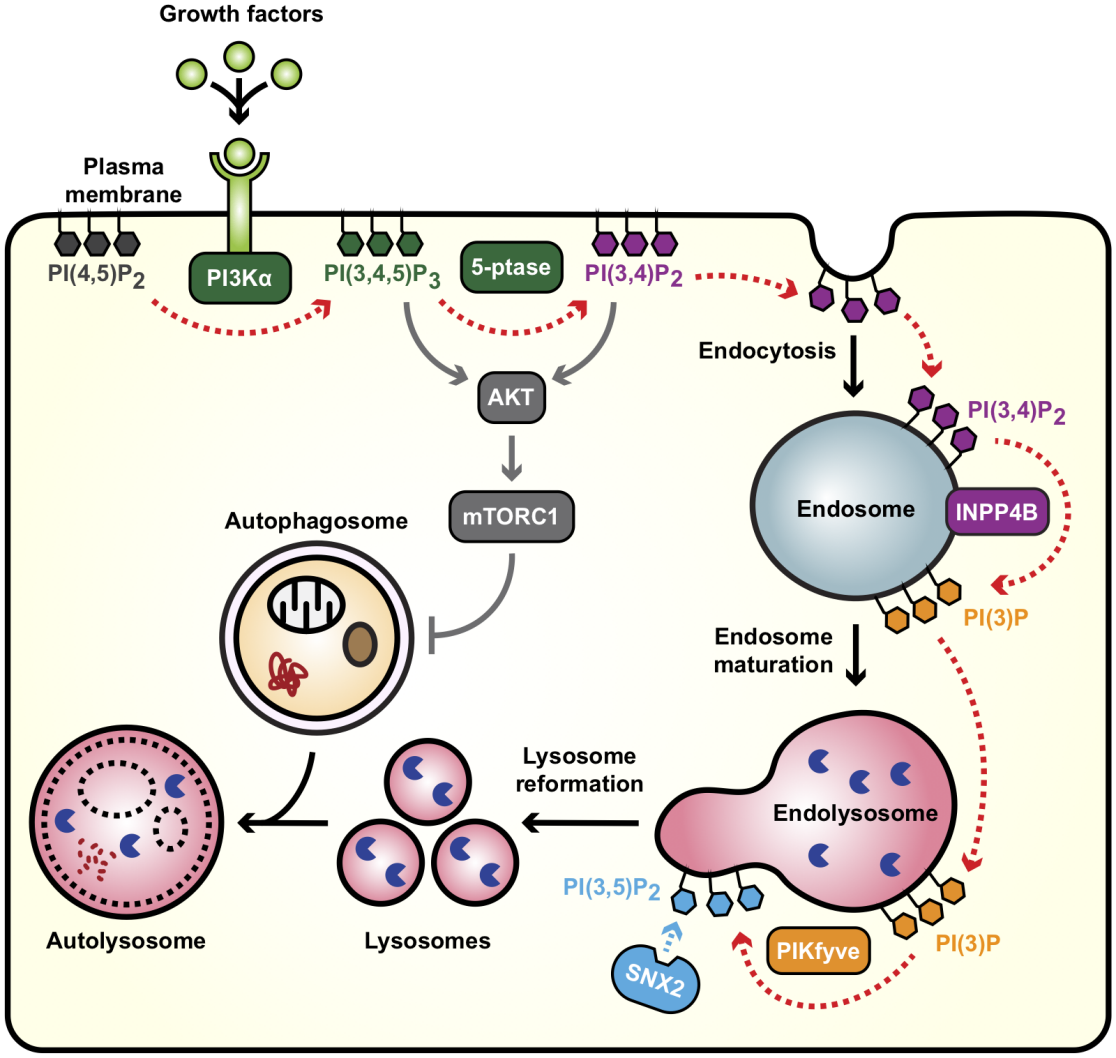
Phosphoinositide conversion via endosomes contributes to lysosome repopulation during basal autophagy Under nutrient‐rich conditions, PI3Kα generates PI(3,4,5)P_3_ at the plasma membrane, which is subsequently hydrolyzed to PI(3,4)P_2_ by inositol polyphosphate 5‐phosphatases. PI(3,4,5)P_3_ and PI(3,4)P_2_ recruit and activate the serine/threonine kinase AKT, which in turn activates mTORC1 to suppress autophagy. Alternatively, PI(3,4)P_2_ is also hydrolyzed by INPP4B on endosomes to PI(3)P, which is retained on endolysosomes following late endosome maturation. PI(3)P is then phosphorylated by PIKfyve to PI(3,5)P_2_, which recruits the SNX‐BAR protein SNX2 to promote lysosome reformation and basal autophagic degradation.

## Discussion

Here, we identify a molecular pathway that controls lysosome repopulation during basal autophagy that is dependent on endosome maturation and lysosome reformation from endolysosomes. PI3Kα generates PI(3,4,5)P_3_ at the plasma membrane in response to growth factor stimulation, which is rapidly hydrolyzed to PI(3,4)P_2_ by inositol polyphosphate 5‐phosphatases (Rodgers *et al*, 2017). INPP4B dephosphorylates this PI(3,4)P_2_ pool to generate PI(3)P on late endosomes (Rodgers *et al*, 2021). Through a comprehensive and systematic examination, we show here that INPP4B‐generated PI(3)P is maintained on endolysosomes following endosome maturation and serves as a substrate for PIKfyve phosphorylation to form PI(3,5)P_2_. The SNX‐BAR protein, SNX2, binds PI(3,5)P_2_ to promote endolysosome tubule formation and lysosome reformation. This molecular pathway is required to ensure lysosome homeostasis during basal autophagy, whereby suppression of phosphoinositide conversion or endosome maturation reduces lysosome numbers and basal autophagic degradation leading to cytotoxicity during proteotoxic stress. Therefore, our data suggest a model whereby PI3Kα signaling initiates a phosphoinositide pathway under nutrient‐rich conditions, which results in PI(3)P generation on late endosomes that promotes PI(3,5)P_2_‐dependent lysosome repopulation during basal autophagy (Fig 8).

The contribution of the class I PI3K signaling network to autophagy is complex, with evidence that class I PI3K effector proteins can promote or suppress autophagy (Yu *et al*, 2015; Manning & Toker, 2017). We demonstrate class I PI3K‐dependent PI(3)P synthesis regulates autophagy in a distinct manner to the canonical Vps34‐dependent PI(3)P pathway, suggesting functional specificity between these different PI(3)P pools. Vps34‐generated PI(3)P is required for starvation‐induced autophagy by promoting autophagosome formation, recruitment of autophagic cargo, autophagosome–lysosome fusion, and the repopulation of lysosomes by ALR (Axe *et al*, 2008; Russell *et al*, 2013; Dooley *et al*, 2014; Munson *et al*, 2015). In contrast, we show that INPP4B‐generated PI(3)P downstream of PI3Kα is required for basal autophagy by promoting lysosome reformation from endolysosomes. Notably, INPP4B‐generated PI(3)P is dispensable during starvation‐induced autophagy where PI3Kα activation is minimal (Manning & Toker, 2017; Rodgers *et al*, 2017). All cellular PI(3,5)P_2_ is generated via PI(3)P phosphorylation by the PIKfyve complex, which contains the catalytic 5‐kinase PIKfyve and two regulatory components, the lipid/protein phosphatase Fig4 and the scaffold protein Vac14 (Zolov *et al*, 2012; Lees *et al*, 2020). PI(3)P to PI(3,5)P_2_ conversion by the PIKfyve complex is required for multiple intracellular trafficking events including endosomal sorting, endosome‐to‐Golgi transport, and lysosome reformation (Rutherford *et al*, 2006; Jefferies *et al*, 2008; Bissig *et al*, 2017; Choy *et al*, 2018). Although a large proportion of the PI(3)P substrate for PIKfyve is synthesized by class III PI3K, there is a distinct Vps34‐independent PI(3)P substrate pool derived from class I PI3K (Ikonomov *et al*, 2015). Recent reports show that class I PI3K‐derived PI(3,4)P_2_ signals are hydrolyzed to PI(3)P on endosomes by INPP4B (Liu *et al*, 2018, 2020; Rodgers *et al*, 2021). Our findings demonstrate that INPP4B‐generated PI(3)P signals on endosomes are retained as this compartment matures into endolysosomes, and in turn PI(3)P is subsequently phosphorylated to PI(3,5)P_2_ by PIKfyve. Therefore, we propose that the lysosomal PI(3,5)P_2_ pool that functions during basal autophagy is derived from sequential action of PI3Kα, INPP4B, and PIKfyve to facilitate lysosome reformation from endolysosomes.

We propose that a functional endosomal system is required to maintain lysosome homeostasis during basal autophagy. There is an emerging body of evidence that autophagosome membranes are derived from recycling endosomes (Longatti *et al*, 2012; Puri *et al*, 2018) or hybrid Golgi‐endosome structures (Kumar *et al*, 2021), suggesting that significant convergence exists between the endosomal and autophagy pathways. However, the complexities of the functional intersection of these two pathways are still emerging. ESCRT proteins, which promote the formation of ILVs within late endosomes to facilitate endosome maturation, are also required for basal autophagic degradation by unknown mechanisms (Filimonenko *et al*, 2007; Rusten *et al*, 2007). Consistent with this, we found that suppression of late endosome maturation via inactivation of INPP4B or the ESCRT protein, Hrs, reduced basal autophagic degradation. Mechanistically, we propose that this block in autophagy results from an inability to retain and/or access PI(3)P on endolysosomes for PIKfyve generation of PI(3,5)P_2_. This reveals an unanticipated level of interplay between endosomal and autophagy pathways, whereby basal autophagy is dependent on the continued maturation of endosomes in order to facilitate PIKfyve‐dependent lysosome reformation.

Our findings provide mechanistic insight into how lysosome membrane recycling occurs under basal autophagy. Although lysosome reformation was first described over a decade ago (Pryor *et al*, 2000; Bright *et al*, 2005), the significant challenges associated with capturing these rapid membrane remodeling events have greatly limited our understanding of the molecular mechanisms that govern this process. Our development of a rapid imaging and quantitative analysis workflow enabled us to examine lysosome reformation in live cells and identify INPP4B, a PI(3)P‐generating enzyme, and SNX2, a PI(3,5)P_2_‐binding effector, as direct regulators of this process. The PI(3,5)P_2_‐binding calcium channel protein, TRPML1, promotes scission of endolysosome tubules (Miller *et al*, 2015), and here we show that SNX2 is required for the initiation and/or extension of endolysosome tubules, suggesting that these PI(3,5)P_2_ effectors work at different stages of lysosome reformation. Some SNX proteins require coincidence detection of multiple phosphoinositides or proteins to regulate their localization and activity (Carlton *et al*, 2004; Daste *et al*, 2017). We demonstrate that SNX2 recruitment to endolysosomes is dependent on PI(3,5)P_2_ generation by PIKfyve. However, as SNX2 can also bind PI(3)P, PI(4)P, and PI(3,4)P_2_ (Carlton *et al*, 2005; Chandra *et al*, 2019), we cannot exclude the possibility that co‐incidence phosphoinositide detection may contribute to SNX2 recruitment or activation on endolysosomes. Furthermore, there is likely to be additional molecular machinery that co‐ordinates lysosome reformation from endolysosomes, such as motor proteins or actin scaffolds that are essential for autolysosome tubule extension during ALR (Rong *et al*, 2012; Du *et al*, 2016; Dai *et al*, 2019; McGrath *et al*, 2021).

Dissecting the pathways that underpin lysosome repopulation during autophagy may provide important insights into the molecular basis of diseases associated with lysosome and autophagy dysfunction. Defective lysosome homeostasis is linked to a wide spectrum of human diseases (Bonam *et al*, 2019). We and others recently uncovered defective ALR leads to muscular and neurological disorders associated with enlarged lysosomal compartments and reduced autophagic capacity (Varga *et al*, 2015; Vantaggiato *et al*, 2019; McGrath *et al*, 2021). It is currently unknown whether defective lysosome reformation from endolysosomes is pathogenic. However, mutations in PIKfyve complex proteins Fig4 and Vac14 cause PI(3,5)P_2_ depletion and neurological disease (Chow *et al*, 2007; Zhang *et al*, 2007, 2008; Zolov *et al*, 2012). There is a strong association with lysosome dysfunction in these disorders, including the presence of enlarged lysosomes in both patient samples and mouse models. The reasons for lysosome enlargement are still emerging, but this is a characteristic feature observed with inhibition of lysosome reformation in cellular models (Bissig *et al*, 2017; Choy *et al*, 2018) and may relate to an inability to regenerate lysosomes from endolysosome membranes. Moreover, PIKfyve inhibition in lymphoma and neuronal cells also causes lysosome swelling and dysfunction leading to autophagosome accumulation (Martin *et al*, 2013; Gayle *et al*, 2017), consistent with a role for PIKfyve in maintaining the supply of lysosomes needed for autophagosome fusion to form autolysosomes. Our data demonstrates that disruption of INPP4B/PIKfyve‐dependent lysosome reformation leads to protein aggregate accumulation and proteotoxicity, processes that are associated with neurological disease (Dubnikov *et al*, 2017). It is interesting to speculate whether reduced lysosome reformation and increased proteotoxic stress may contribute to neurological disease resulting from PIKfyve complex inactivation, and in turn, whether dysregulation of lysosome reformation from endolysosomes may impact on other human diseases.

## Materials and Methods

### Cell culture

MCF‐7 (cat # HTB‐22), HeLa cells (cat # CCL2), and HEK293T (cat # CRL‐3216) cells were purchased from ATCC. MCF‐7 cells were cultured in DMEM supplemented with 10% (v/v) FCS, 2 mM L‐glutamine, 100 units/ml penicillin, 1% (v/v) streptomycin, and 10 μg/ml insulin. HeLa and HEK293T were cultured in DMEM supplemented with 10% (v/v) FCS, 2 mM L‐glutamine, 100 units/ml penicillin, and 1% (v/v) streptomycin. All cells were maintained in a 5% CO_2_‐humidified 37°C incubator. All aseptic culture techniques were performed in a class II biohazard hood. Cells were routinely tested to confirm the absence of mycoplasma contamination. Cell line authentication was not performed.

For nutrient deprivation, cells were washed twice with PBS then incubated in EBSS (Sigma, cat # E3024) for 2–6 h as indicated. For experiments using SAR405 (Selleckchem, # S7682), bafilomycin A1 (Sigma, cat # B1793), or YM201636 (Selleckchem, cat # S1219), cells were treated with the indicated dose or the same volume of DMSO as a vehicle control. For YM201636 washout experiments, cells were washed twice with growth media then incubated with fresh growth media.

### cDNA constructs

To generate pBMN‐LAMP1‐mCherry and pBMN‐LAMP1‐mEGFP, the pBMN‐Z retroviral vector (Gary Nolan, Stanford University, Addgene, cat # 1734) was digested with *Sal1*/*BamH1* before cloning using the HiFi DNA Assembly 1232 Kit (New England Biolabs, cat # E5520S) according to the manufacturer's instructions. LAMP1‐mCherry and LAMP1‐mEGFP were generated by PCR amplification of LAMP1‐RFP (Sherer *et al*, 2003; Addgene, cat # 1817) and either pBMN‐mCherry‐C1 (Michael Lazarou, Walter and Eliza Hall Institute of Medical Research and Monash University, Australia; Padman *et al*, 2019), or pBMN‐mEGFP‐INPP5K (McGrath *et al*, 2021), respectively. pCGN‐HA‐INPP4B^WT^ and pCGN‐HA‐INPP4B^C842A^ were generated by digesting pCGN‐HA (Gurung *et al*, 2003) with *Kpn1*. INPP4B^WT^ or INPP4B^C842A^ (Rijal *et al*, 2015) were amplified with *Kpn1* digestion sites, then digested and cloned into the *Kpn1* site of pCGN‐HA. All generated plasmid DNA sequences were verified by Sanger sequencing (Micromon, Monash University, Australia). pEGFP‐SNX2 was a kind gift from Brett Collins (University of Queensland, Australia). pEGFP‐PIKFYVE was a kind gift from Geert van den Bogaart (Addgene, cat # 121148; Baranov *et al*, 2019). pEGFP‐C2 was purchased from Clontech (cat # 6083‐1).

### Generation of stable cell lines by viral transduction

Retroviral and lentiviral transductions were carried out as previously described (Lazarou *et al*, 2015; Rodgers *et al*, 2021). Cells transduced with lentiviral particles encoding pHIV‐1SDmCMV.pre GFP‐vector, pHIV‐1SDmCMV.pre GFP‐INPP4B (Fedele *et al*, 2010), pBMN‐LAMP1‐mEGFP, or pBMN‐LAMP1‐mCherry were selected by fluorescent activated cell sorting (FACS) (Flowcore, Monash University, Australia). Cells transduced with pLKO.1‐puro Non‐Mammalian shRNA (Sigma, cat # SHC002), *INPP4B* #1 mission^®^ shRNA (Sigma, cat # TRCN0000052721), *INPP4B* #2 mission^®^ shRNA (Sigma, cat # TRCN0000052722), or *HGS* mission® shRNA (Sigma, cat # TRCN000038‐0920) lentiviral particles were selected by culturing cells in media containing 1 μg/ml puromycin (Sigma, cat # P9620) for 1 week, then cultured into puromycin‐free media at least 1 week before experiments were conducted.

### Cell transfections

pCGN‐HA‐vector, pCGN‐HA‐INPP4B^WT^, pCGN‐HA‐INPP4B^C842A^, pEGFP‐PIKFYVE, pEGFP‐SNX2, or pEGFP‐C2 were transfected into cells using Lipofectamine 3000 (Invitrogen, cat # L3000001) according to the manufacturer's instructions. Cells were co‐stained with HA or GFP antibodies (Appendix Table S1) in all experiments in order to detect transfected cells. For siRNA depletion, cells were reverse transfected with *INPP4B* siRNA (Qiagen, cat # SI05133338), *PIK3CA* siRNA #1 (Qiagen, cat # SI02622207), *PIK3CA* siRNA #2 (Qiagen, cat # SI02665369), *PIP5K3* siRNA #1 (Qiagen, cat # SI03063928), *PIP5K3* siRNA #2 (Qiagen, cat # SI05049359), *SNX1* siRNA (Qiagen, cat # SI00047775), *SNX2* siRNA (Qiagen, cat # SI04258394), *SNX2* 3'‐UTR siRNA (Qiagen, cat # SI05077534), or NT siRNA (Qiagen, cat # 1027281) using Lipofectamine RNAiMAX (Invitrogen, cat # 13778075) according to the manufacturer's directions (*Transfecting Stealth™ RNAi or siRNA into MCF7 Cells Using Lipofectamine™ RNAiMAX*). Following transfections, cells were incubated for 24 h at 37°C before experiments were conducted.

### Immunoblotting

Cell lysates were prepared for immunoblotting by washing cells once with TBS on ice followed by direct cell lysis in 40 mM of Tris pH 6.8, 4% (w/v) SDS, 20% (v/v) glycerol, 0.0002% (w/v) bromophenol blue, 50 mM of DTT. Lysates were boiled for 5 min at 100°C, and proteins were separated by 10% SDS‐PAGE at 150 V for 1–1.5 h. LC3B proteins were transferred to PDVF by electrophoresis at 100 mA for 2 h. All other proteins were transferred to PVDF at 250 mA for 1.5 h. Immunoblot blocking solution (5% skim milk in TBS) was added to membranes for 1 h at room temperature while rocking. Membranes were incubated in primary antibodies (Appendix Table S1) diluted in TSB‐T overnight at 4°C while rocking. Membranes were washed three times with TBS‐T while rocking for 10 min each. Secondary HRP‐conjugated antibodies (Appendix Table S1) diluted in TBS‐T were added for 1 h at room temperature then membranes were washed three times in TBS‐T while rocking for 10 min each. Membranes were immersed in ECL Plus for 1 min, then exposed to X‐ray film in a dark room and developed using a Fuji processor. Densitometry with ImageJ version 2.0.0 software (https://imagej.nih.gov) (Schneider *et al*, 2012) was used to quantify protein bands, with signals being normalized to the loading control protein GAPDH.

### Immunofluorescence

#### Immunofluorescence of lysosomes and endosomes

Endosomes and lysosomes were visualized by immunofluorescence using a saponin‐based protocol that preserves endosomal structures (Scheffler *et al*, 2014). Cells were seeded onto 15 mm round coverslips. After 24 h, cells were fixed with 4% (w/v) PFA for 20 min, then washed three times in 50 mM of NH_4_Cl in PBS. Cells were blocked and permeabilized in 2% (w/v) BSA, 0.05% (w/v) saponin in PBS for 1 h. Primary antibodies (Appendix Table S1) were diluted in blocking solution and added overnight at 4°C, then cells were washed three times in PBS. Alexa Fluor® secondary antibodies, phalloidin and DAPI (Appendix Table S1) were diluted in blocking solution and added for 1 h at room temperature. Cells were washed three times with PBS, and mounted onto slides with Fluoromount‐G. Slides were imaged using a Leica SP8 invert confocal laser scanning microscope (Monash Micro Imaging, Monash University, Australia) and LAS X version 3.5.6.21594 software (Leica, https://www.leica‐microsystems.com/). Single z‐plane images were taken using the same laser power for all conditions within the same experiment. For super resolution microscopy, slides were imaged using a Zeiss LSM980 confocal laser scanning microscope with an Airyscan 2 detector (Monash Micro Imaging, Monash University, Australia) and ZEN version 3.3 (Zeiss, https://www.zeiss.com/).

To visualize INPP4B at lysosomes and endosomes, cells were pretreated with saponin before fixation to remove cytoplasmic proteins and retain proteins bound by intracellular membranes as previously described (Marat *et al*, 2017). Media was aspirated from cells, then cells were permeabilized with 0.02% (w/v) saponin, 25 mM of KCl, 2.5 mM of MgCl_2_, 25 mM of HEPES, pH 7.4 for 30 s. Cells were then fixed and stained using the saponin‐based protocol as described above.

#### Immunofluorescence of autophagosomes and protein aggregates

Cells were seeded onto 15 mm coverslips. The following day, cells washed three times with PBS and fixed with 4% (w/v) PFA for 30 min. Cells were washed three times in PBS. Cells were permeabilized with 0.1% (v/v) Triton X‐100 in PBS for 90 s. Cells were washed three times with PBS. Cells were blocked in 3% (w/v) BSA in PBS for 1 h. Primary antibodies (Appendix Table S1) were diluted in blocking solution and added for 1 h. Cells were washed three times with PBS. Alexa Fluor^®^ secondary antibodies, phalloidin, and DAPI (Appendix Table S1) were diluted in block and added for 1 h. Cells were washed three times with PBS and mounted on to slides with Fluoromount G. Slides were imaged using a Leica SP8 invert confocal laser scanning microscope and LAS X version 3.5.6.21594 software. Single z‐plane images were taken using the same laser power for all conditions within the same experiment.

#### GFP‐mCherry‐LC3B biosensor

Cells were transduced with retroviral particles encoding pBABE‐puro‐mCherry‐EGFP‐LC3B (Addgene, cat #22418; N'Diaye *et al*, 2009) as previously described (Lazarou *et al*, 2015). Following transduction, cells were seeded onto 15 mm round coverslips. The following day, cells were fixed in 4% (w/v) PFA for 20 min, then washed three times with PBS. DAPI was diluted in PBS and added to cells for 30 min, then cells were washed three times with PBS. Coverslips were mounted onto slides with Fluoromount‐G and imaged using a Leica SP8 invert confocal laser scanning microscope and LAS X version 3.5.6.21594 software. Single z‐plane images were taken using the same laser power for all conditions within the same experiment.

#### Detection of PI(3)P by immunofluorescence

PI(3)P staining was performed as previously described using the “Golgi” staining method (Hammond *et al*, 2009). Briefly, cells were seeded onto 15 mm round coverslips. The following day, cells were fixed in 2% (w/v) PFA for 15 min. Cells were washed three times with 50 mM of NH_4_Cl in PBS, and then permeabilized with Buffer A (20 mM PIPES, 137 mM NaCl, 2.7 mM KCl, pH 6.8) with 20 μM of digitonin for 5 min. Cells were washed three times in Buffer A and then blocked with 5% (v/v) goat serum, 50 mM of NH_4_Cl in Buffer A for 45 min. Eight μg/ml of recombinant GST‐2xFYVE^Hrs^ (Naughtin *et al*, 2010) was diluted with 5% (v/v) goat serum in Buffer A and added to cells for 45 min. Cells were washed twice with Buffer A then primary antibodies (Appendix Table S1) were diluted in 5% (v/v) goat serum in Buffer A and added to cells for 1.5 h. Cells were washed twice with Buffer A. Alexa Fluor® secondary antibodies and DAPI (Appendix Table S1) were diluted with 5% (v/v) goat serum in Buffer A and added to cells for 45 min. Cells were washed four times with Buffer A. Cells were postfixed with 2% (w/v) PFA then washed three times in 50 mM of NH_4_Cl in PBS and once with dH_2_O. Coverslips were mounted onto slides with Fluoromount‐G and imaged using a Leica SP8 invert confocal laser scanning microscope and LAS X version 3.5.6.21594 software. Single z‐plane images were taken using the same laser power for all conditions within the same experiment.

#### Particle analysis

Images were analyzed using ImageJ version 2.0.0 software. For particle analysis, the same channel threshold was applied to all images from the same experiment. The “*analyse particle*” plugin was used to determine the number and/or size of p62‐positive, LAMP1‐positive, or LAMP2‐positive puncta greater than 0.1 μm^2^ per cell. To determine the distance of lysosomes from the nucleus, the centroid coordinates of individual lysosomes and nuclei were used to determine distance using the equation: distance = √((lysosome *x* coordinate − nucleus *x* coordinate)^2^ + (lysosome *y* coordinate − nucleus *y* coordinate)^2^). Particle co‐localization analysis was performed as previously described (De Leo *et al*, 2016). To determine the proportion of PI(3)P‐positive lysosomes, SNX2‐positive lysosomes, or SNX2‐positive early endosomes, the LAMP1‐positive or EEA1‐positive puncta were used to construct a mask that was overlaid with the 2xFYVE‐positive or SNX2‐positive puncta. To quantify CD63‐positive/p62‐positive amphisomes, the CD63‐positive puncta were used to construct a mask that was overlaid with the p62‐positive puncta. The number of double positive puncta with > 30% overlap in area were quantified. To determine lysosomal phospho‐mTOR staining intensity, the LAMP1 channel was used to construct a mask that was overlaid with the phospho‐mTOR staining. Fluorescence intensity of phospho‐mTOR signals co‐localizing with LAMP1 were calculated and normalized to cell area.

### Live cell imaging

#### Magic Red™ cathepsin B

Magic Red™ cathepsin B assays (Bio‐Rad, cat # 6133) were carried out according to the manufacturer's instructions. Cells were seeded in 4‐well chamber slides. The following day, cells were washed once with PBS, then incubated for 30 min with Magic Red™ cathepsin B and 1 μg/ml Hoechst 33342 diluted in live cell media containing phenol red‐free DMEM, 10% (v/v) FCS, and 10 μg/ml insulin. Live cells were imaged in a 5% CO_2_‐humidified 37°C chamber using a Leica SP8 invert confocal laser scanning microscope and LAS X version 3.5.6.21594 software. Single z‐plane images were taken using the same laser power for all conditions within the same experiment. The “*analyse particle*” plugin was used to determine the number of Magic Red™ cathepsin B‐positive puncta per cell, with the same channel threshold applied to each image in the same experiment. To quantify LAMP1‐positive/Magic Red™ cathepsin B‐negative terminal storage lysosomes, the LAMP1‐positive puncta were used to construct a mask that was overlaid with the Magic Red™ cathepsin B‐positive puncta.

#### Lysosome reformation

Cells expressing LAMP1‐mCherry were seeded in a 35 mm FluoroDish. The following day, cells were washed once with PBS then phenol red‐free DMEM containing 10% (v/v) FCS and 10 μg/ml insulin were added. Live cells were imaged in a 5% CO_2_‐humidified 37°C chamber using a 3i Marianas Spinning Disk microscope (Monash Micro Imaging, Monash University, Australia) and Slidebook version 6.0.22 (Intelligent Imaging Innovations, https://www.intelligent‐imaging.com/). To visualize lysosome reformation, three z‐planes 0.27 μm apart were acquired every 500 or 1,000 ms for 20 min using the same laser power for all conditions within the same experiment. Maximum intensity projections of lysosomes were converted into three‐dimensional kymographs using *x*, *y*, and time axes, and lysosome reformation analysis was performed using Dragonfly software version 2020.2 (Object Research Systems Inc, http://www.theobjects.com/dragonfly). Briefly, kymographs of lysosomes were segmented using OTSU's method for thresholding (Otsu, 1979), and skeletonized to detect tubulations events leading to scissions as branches off the main stem that represents the parent lysosome. The skeletons were then analyzed for nodes and branches in order identify and quantify lysosome reformation events (Appendix Fig S5A–E).

To visualize SNX2 at lysosomes undergoing reformation, MCF‐7 cells expressing LAMP1‐mCherry were transfected with pEGFP‐SNX2. After 24 h, cells were washed once with PBS then phenol red‐free DMEM containing 10% (v/v) FCS and 10 μg/ml insulin were added. Live cells were imaged in a 5% CO_2_‐humidified 37°C chamber using a 3i Marianas Spinning Disk microscope and Slidebook version 6.0.22. Two z‐planes 0.27 μm apart were acquired every 1,000 ms for 20 min.

### Immuno‐gold labeling of cryosections for transmission electron microscopy

Tokuyasu sample preparation and immuno‐gold labeling of cryosections for transmission electron microscopy was performed as described (Slot & Geuze, 2007; Oorschot *et al*, 2021). A version of the protocol is maintained at protocols.io (dx.doi.org/10.17504/protocols.io.btmrnk56). Cell monolayers were cultured in 10 cm plates and allowed to reach 80% confluency before being fixed overnight at 4°C with 0.1 M phosphate buffered 2% (w/v) PFA and 0.2% (w/v) glutaraldehyde. Cells were washed in PBS (3 × 10 min) and then rinsed for 10 min in 0.15% (w/v) glycine in PBS. The fixed samples were scraped in 1% (w/v) gelatin in 0.1 M phosphate buffer and infused for 30 min at 37°C with 12% (w/v) gelatin in 0.1 M phosphate buffer, which was subsequently solidified at 4°C before being cut into small cubes measuring ~0.5 mm on each edge. The gelatin embedded cells were infiltrated with 2.3 M sucrose in 0.1 M phosphate buffer at 4°C overnight, rotating. Sucrose‐infiltrated cell blocks were mounted on aluminum cryo‐pins and then frozen in liquid nitrogen for cryo‐ultramicrotomy.

Frozen cell blocks were trimmed at −100°C with a cutting speed of 100 mm/s and a 100 nm feed, and then sectioned at −120°C with an 0.8 mm/s cutting speed and 62 nm feed using a UC7/FC7 cryo‐ultramicrotome (Leica Microsystems) equipped with a 20° cryo‐trim diamond knife (Diatome) and a 35° cryo‐immuno diamond knife (Diatome), respectively. Cryosections were retrieved using a stainless steel loop with a droplet of phosphate buffered 1% (w/v) methyl cellulose and 1.15 M sucrose, then deposited on formvar‐coated grids for immunolabeling.

Samples were prepared for immunolabeling by floating grids section‐side down in PBS at 37°C for 1 h. Grids were then rinsed with 0.15% (w/v) glycine in PBS (3 × 2 min) and blocked with 1% (w/v) BSA in PBS for 5 min, before incubation with rabbit anti‐GFP primary antibodies (Appendix Table S1) in 1% (w/v) BSA in PBS for 1 h at room temperature. Following washes in 0.1% (w/v) BSA in PBS (5 × 2 min), grids were incubated with Protein‐A conjugated 10‐nm gold particles (1:50, Cell Biology, UMC Utrecht, Netherlands) diluted in 1% (w/v) BSA in PBS for 30 min at room temperature and then washed in PBS (5 × 2 min). The labeling reaction was stabilized by fixation with 1% (w/v) glutaraldehyde in PBS for 5 min and grids rinsed in distilled water (6 × 1 min). Grids were then contrasted with 2% (w/v) uranyloxalate (pH 7) for 5 min at room temperature, and floated twice briefly and then for 10 min on ice cold 0.4% (w/v) uranyl acetate in 1.8% (w/v) aqueous methyl cellulose (pH 4). Finally, grids were looped out and dried in a thin film of 0.4% (w/v) uranyl acetate in 1.8% (w/v) aqueous methyl cellulose (pH 4) using a wire loop. High resolution electron micrographs were acquired using a JEOL‐1400 Plus transmission electron microscope at 80 keV and TEM Centre version 1.7.18.2349 software (Monash Ramaciotti Centre for Cryo‐Electron Microscopy, Monash University, Australia).

### RNA analysis

RNA was extracted from cells using the Isolate II RNA extraction kit (Bioline, cat # BIO‐52073) according to the manufacturer's instructions. RNA concentration was determined using a Nanodrop 1000 spectrophotometer (Thermo Fisher Scientific). Extracted RNA was diluted to 50 ng/ml, and subjected to two‐step qRT‐PCR using the iScript gDNA clear cDNA synthesis kit (Bio‐Rad, cat # 172‐5035) and the QuantiTect SYBR Green PCR Kit (Qiagen, cat # 204143) according to the manufacturers' instructions. Reactions were subjected to thermocycling using a CFX384 Real Time PCR System (Bio‐Rad), and analyzed using CFX Manager version 3.1 software (Bio‐Rad, https://www.bio‐rad.com). All qRT‐PCR primers are listed in Appendix Table S2. A no template control without cDNA and a no reverse transcriptase control were included to ensure no genomic DNA was present. The relative expression of the gene of interest was compared to the standard loading control genes *ACTB* or *RRN18S* and quantified using the ΔΔCT method (Dussault & Pouliot, 2006).

### Cell viability assays

Cell viability was assessed using CellTiter‐Glo® 3D Cell Viability Assay (Promega, cat # G9681). Briefly, ~24,000 cells were seeded per well in a 24‐well plate. The following day, cells were treated with 10 μg/ml puromycin and/or 5 μM YM201636 for 2, 4, or 6 h. At the treatment endpoint, media was removed and replaced with 200 μl of fresh media without inhibitors and 200 μl CellTiter reagent. The plate was shaken for 5 min and left to incubate in the dark for 30 min. The integrated luminescence signal was detected using a BMG LABTECH CLARIOstar Plus plate reader with CLARIOstar version 5.20 R5 and MARS 3.10 R6 software.

### Statistics and reproducibility

Genetically modified cells were derived from the same pool of parent cells. Cells were randomly assigned to treatment or control groups. No sample size calculations were performed. Blinding was not applied to experiments. All experiments were repeated at least three times independently to ensure statistical significance of the results. Statistical analysis was performed using Prism version 7.0 (GraphPad, https://www.graphpad.com). Two‐tailed unpaired *t* test was used for experiments with two groups, one‐way ANOVA was used for experiments with more than two groups and one independent variable, and two‐way ANOVA was used for experiments with more than two groups and two independent variables. Details of statistical testing can be found in the figure legends. Differences between groups were considered statistically different for *P* values < 0.05.

## Author contributions

**Samuel J Rodgers:** Conceptualization; formal analysis; supervision; investigation; methodology; writing – original draft; writing – review and editing. **Emily I Jones:** Formal analysis; investigation; writing – review and editing. **Senthil Arumugam:** Resources; formal analysis; writing – review and editing. **Sabryn A Hamila:** Validation; investigation; writing – review and editing. **Jill Danne:** Investigation; writing – review and editing. **Rajendra Gurung:** Methodology; writing – review and editing. **Matthew J Eramo:** Methodology; writing – review and editing. **Randini Nanayakkara:** Methodology; writing – review and editing. **Georg Ramm:** Resources; funding acquisition; writing – review and editing. **Meagan J McGrath:** Conceptualization; supervision; funding acquisition; writing – original draft; writing – review and editing. **Christina A Mitchell:** Conceptualization; resources; supervision; funding acquisition; writing – original draft; writing – review and editing.

## Disclosure and competing interests statement

The authors declare that they have no conflict of interest.

## Supporting information




Appendix

Expanded View Figures PDF

Movie EV1

Movie EV2

Movie EV3

Movie EV4

Source Data for Expanded View and Appendix

Source Data for Figure 1

Source Data for Figure 2

Source Data for Figure 3

Source Data for Figure 4

Source Data for Figure 5

Source Data for Figure 6

Source Data for Figure 7

PDF+

## Data Availability

This study includes no data deposited in external repositories. Source data and uncropped blots are provided with this paper. All data that support the findings of this study are available from the corresponding author upon reasonable request.

## References

[embj2021110398-bib-0001] Axe EL , Walker SA , Manifava M , Chandra P , Roderick HL , Habermann A , Griffiths G , Ktistakis NT (2008) Autophagosome formation from membrane compartments enriched in phosphatidylinositol 3‐phosphate and dynamically connected to the endoplasmic reticulum. J Cell Biol 182: 685–701 18725538 10.1083/jcb.200803137 PMC2518708

[embj2021110398-bib-0002] Bache KG , Stuffers S , Malerød L , Slagsvold T , Raiborg C , Lechardeur D , Wälchli S , Lukacs GL , Brech A , Stenmark H (2006) The ESCRT‐III subunit hVps24 is required for degradation but not silencing of the epidermal growth factor receptor. Mol Biol Cell 17: 2513–2523 16554368 10.1091/mbc.E05-10-0915 PMC1474783

[embj2021110398-bib-0003] Baranov MV , Bianchi F , Schirmacher A , van Aart MAC , Maassen S , Muntjewerff EM , Dingjan I , Ter Beest M , Verdoes M , Keyser SGL *et al* (2019) The phosphoinositide kinase PIKfyve promotes cathepsin‐S‐mediated major histocompatibility complex class II antigen presentation. iScience 11: 160–177 30612035 10.1016/j.isci.2018.12.015 PMC6319320

[embj2021110398-bib-0004] Bissig C , Hurbain I , Raposo G , van Niel G (2017) PIKfyve activity regulates reformation of terminal storage lysosomes from endolysosomes. Traffic 18: 747–757 28857423 10.1111/tra.12525

[embj2021110398-bib-0005] Bonam SR , Wang F , Muller S (2019) Lysosomes as a therapeutic target. Nat Rev Drug Discov 18: 923–948 31477883 10.1038/s41573-019-0036-1 PMC7097195

[embj2021110398-bib-0006] Boukhalfa A , Nascimbeni AC , Ramel D , Dupont N , Hirsch E , Gayral S , Laffargue M , Codogno P , Morel E (2020) PI3KC2α‐dependent and VPS34‐independent generation of PI3P controls primary cilium‐mediated autophagy in response to shear stress. Nat Commun 11: 294 31941925 10.1038/s41467-019-14086-1 PMC6962367

[embj2021110398-bib-0007] Bright NA , Davis LJ , Luzio JP (2016) Endolysosomes are the principal intracellular sites of acid hydrolase activity. Curr Biol 26: 2233–2245 27498570 10.1016/j.cub.2016.06.046 PMC5026700

[embj2021110398-bib-0008] Bright NA , Gratian MJ , Luzio JP (2005) Endocytic delivery to lysosomes mediated by concurrent fusion and kissing events in living cells. Curr Biol 15: 360–365 15723798 10.1016/j.cub.2005.01.049

[embj2021110398-bib-0009] Carlton J , Bujny M , Peter BJ , Oorschot VM , Rutherford A , Mellor H , Klumperman J , McMahon HT , Cullen PJ (2004) Sorting nexin‐1 mediates tubular endosome‐to‐TGN transport through coincidence sensing of high‐ curvature membranes and 3‐phosphoinositides. Curr Biol 14: 1791–1800 15498486 10.1016/j.cub.2004.09.077

[embj2021110398-bib-0010] Carlton JG , Bujny MV , Peter BJ , Oorschot VM , Rutherford A , Arkell RS , Klumperman J , McMahon HT , Cullen PJ (2005) Sorting nexin‐2 is associated with tubular elements of the early endosome, but is not essential for retromer‐mediated endosome‐to‐TGN transport. J Cell Sci 118: 4527–4539 16179610 10.1242/jcs.02568 PMC1904489

[embj2021110398-bib-0011] Catimel B , Schieber C , Condron M , Patsiouras H , Connolly L , Catimel J , Nice EC , Burgess AW , Holmes AB (2008) The PI(3,5)P2 and PI(4,5)P2 interactomes. J Proteome Res 7: 5295–5313 19367725 10.1021/pr800540h

[embj2021110398-bib-0012] Chandra M , Chin YK , Mas C , Feathers JR , Paul B , Datta S , Chen KE , Jia X , Yang Z , Norwood SJ *et al* (2019) Classification of the human phox homology (PX) domains based on their phosphoinositide binding specificities. Nat Commun 10: 1528 30948714 10.1038/s41467-019-09355-y PMC6449406

[embj2021110398-bib-0013] Chen D , Fan W , Lu Y , Ding X , Chen S , Zhong Q (2012) A mammalian autophagosome maturation mechanism mediated by TECPR1 and the Atg12‐Atg5 conjugate. Mol Cell 45: 629–641 22342342 10.1016/j.molcel.2011.12.036 PMC3299828

[embj2021110398-bib-0014] Chow CY , Zhang Y , Dowling JJ , Jin N , Adamska M , Shiga K , Szigeti K , Shy ME , Li J , Zhang X *et al* (2007) Mutation of FIG4 causes neurodegeneration in the pale tremor mouse and patients with CMT4J. Nature 448: 68–72 17572665 10.1038/nature05876 PMC2271033

[embj2021110398-bib-0015] Choy CH , Saffi G , Gray MA , Wallace C , Dayam RM , Ou ZA , Lenk G , Puertollano R , Watkins SC , Botelho RJ (2018) Lysosome enlargement during inhibition of the lipid kinase PIKfyve proceeds through lysosome coalescence. J Cell Sci 131: jcs213587 29661845 10.1242/jcs.213587 PMC6031331

[embj2021110398-bib-0016] Cozier GE , Carlton J , McGregor AH , Gleeson PA , Teasdale RD , Mellor H , Cullen PJ (2002) The phox homology (PX) domain‐dependent, 3‐phosphoinositide‐mediated association of sorting nexin‐1 with an early sorting endosomal compartment is required for its ability to regulate epidermal growth factor receptor degradation. J Biol Chem 277: 48730–48736 12198132 10.1074/jbc.M206986200

[embj2021110398-bib-0017] Dai A , Yu L , Wang HW (2019) WHAMM initiates autolysosome tubulation by promoting Actin polymerization on autolysosomes. Nat Commun 10: 3699 31420534 10.1038/s41467-019-11694-9 PMC6697732

[embj2021110398-bib-0018] Daste F , Walrant A , Holst MR , Gadsby JR , Mason J , Lee JE , Brook D , Mettlen M , Larsson E , Lee SF *et al* (2017) Control of Actin polymerization via the coincidence of phosphoinositides and high membrane curvature. J Cell Biol 216: 3745–3765 28923975 10.1083/jcb.201704061 PMC5674896

[embj2021110398-bib-0019] De Leo MG , Staiano L , Vicinanza M , Luciani A , Carissimo A , Mutarelli M , Di Campli A , Polishchuk E , Di Tullio G , Morra V *et al* (2016) Autophagosome‐lysosome fusion triggers a lysosomal response mediated by TLR9 and controlled by OCRL. Nat Cell Biol 18: 839–850 27398910 10.1038/ncb3386 PMC5040511

[embj2021110398-bib-0020] Dikic I , Elazar Z (2018) Mechanism and medical implications of mammalian autophagy. Nat Rev Mol Cell Biol 19: 349–364 29618831 10.1038/s41580-018-0003-4

[embj2021110398-bib-0021] Dooley HC , Razi M , Polson HEJ , Girardin SE , Wilson MI , Tooze SA (2014) WIPI2 links LC3 conjugation with PI3P, autophagosome formation, and pathogen clearance by recruiting Atg12‐5‐16L1. Mol Cell 55: 238–252 24954904 10.1016/j.molcel.2014.05.021 PMC4104028

[embj2021110398-bib-0022] Dou Z , Chattopadhyay M , Pan J‐A , Guerriero JL , Jiang Y‐P , Ballou LM , Yue Z , Lin RZ , Zong W‐X (2010) The class IA phosphatidylinositol 3‐kinase p110‐beta subunit is a positive regulator of autophagy. J Cell Biol 191: 827–843 21059846 10.1083/jcb.201006056 PMC2983054

[embj2021110398-bib-0023] Dou Z , Pan JA , Dbouk HA , Ballou LM , DeLeon JL , Fan Y , Chen JS , Liang Z , Li G , Backer JM *et al* (2013) Class IA PI3K p110β subunit promotes autophagy through Rab5 small GTPase in response to growth factor limitation. Mol Cell 50: 29–42 23434372 10.1016/j.molcel.2013.01.022 PMC3628298

[embj2021110398-bib-0024] Du W , Su QP , Chen Y , Zhu Y , Jiang D , Rong Y , Zhang S , Zhang Y , Ren H , Zhang C *et al* (2016) Kinesin 1 drives autolysosome Tubulation. Dev Cell 37: 326–336 27219061 10.1016/j.devcel.2016.04.014

[embj2021110398-bib-0025] Dubnikov T , Ben‐Gedalya T , Cohen E (2017) Protein quality control in health and disease. Cold Spring Harb Perspect Biol 9: a023523 27864315 10.1101/cshperspect.a023523 PMC5334259

[embj2021110398-bib-0026] Dussault A‐A , Pouliot M (2006) Rapid and simple comparison of messenger rna levels using real‐time PCR. Biol Proced Online 8: 1–10 16446781 10.1251/bpo114 PMC1352391

[embj2021110398-bib-0027] Fan W , Tang Z , Chen D , Moughon D , Ding X , Chen S , Zhu M , Zhong Q (2010) Keap1 facilitates p62‐mediated ubiquitin aggregate clearance via autophagy. Autophagy 6: 614–621 20495340 10.4161/auto.6.5.12189 PMC4423623

[embj2021110398-bib-0028] Fedele CG , Ooms LM , Ho M , Vieusseux J , O'Toole SA , Millar EK , Lopez‐Knowles E , Sriratana A , Gurung R , Baglietto L *et al* (2010) Inositol polyphosphate 4‐phosphatase II regulates PI3K/Akt signaling and is lost in human basal‐like breast cancers. Proc Natl Acad Sci USA 107: 22231–22236 21127264 10.1073/pnas.1015245107 PMC3009830

[embj2021110398-bib-0029] Filimonenko M , Stuffers S , Raiborg C , Yamamoto A , Malerød L , Fisher EM , Isaacs A , Brech A , Stenmark H , Simonsen A (2007) Functional multivesicular bodies are required for autophagic clearance of protein aggregates associated with neurodegenerative disease. J Cell Biol 179: 485–500 17984323 10.1083/jcb.200702115 PMC2064794

[embj2021110398-bib-0030] Gayle S , Landrette S , Beeharry N , Conrad C , Hernandez M , Beckett P , Ferguson SM , Mandelkern T , Zheng M , Xu T *et al* (2017) Identification of apilimod as a first‐in‐class PIKfyve kinase inhibitor for treatment of B‐cell non‐Hodgkin lymphoma. Blood 129: 1768–1778 28104689 10.1182/blood-2016-09-736892 PMC5766845

[embj2021110398-bib-0031] Gewinner C , Wang ZC , Richardson A , Teruya‐Feldstein J , Etemadmoghadam D , Bowtell D , Barretina J , Lin WM , Rameh L , Salmena L *et al* (2009) Evidence that inositol polyphosphate 4‐phosphatase type II is a tumor suppressor that inhibits PI3K signaling. Cancer Cell 16: 115–125 19647222 10.1016/j.ccr.2009.06.006 PMC2957372

[embj2021110398-bib-0032] Gillooly DJ , Morrow IC , Lindsay M , Gould R , Bryant NJ , Gaullier JM , Parton RG , Stenmark H (2000) Localization of phosphatidylinositol 3‐phosphate in yeast and mammalian cells. EMBO J 19: 4577–4588 10970851 10.1093/emboj/19.17.4577 PMC302054

[embj2021110398-bib-0033] Gurung R , Tan A , Ooms LM , McGrath MJ , Huysmans RD , Munday AD , Prescott M , Whisstock JC , Mitchell CA (2003) Identification of a novel domain in two mammalian inositol‐polyphosphate 5‐phosphatases that mediates membrane ruffle localization. The inositol 5‐phosphatase skip localizes to the endoplasmic reticulum and translocates to membrane ruffles following epidermal growth factor stimulation. J Biol Chem 278: 11376–11385 12536145 10.1074/jbc.M209991200

[embj2021110398-bib-0034] Hammond GR , Schiavo G , Irvine RF (2009) Immunocytochemical techniques reveal multiple, distinct cellular pools of PtdIns4P and PtdIns(4,5)P(2). Biochem J 422: 23–35 19508231 10.1042/BJ20090428 PMC2722159

[embj2021110398-bib-0035] Hammond GRV , Takasuga S , Sasaki T , Balla T (2015) The ML1Nx2 phosphatidylinositol 3,5‐bisphosphate probe shows poor selectivity in cells. PLoS ONE 10: e0139957 26460749 10.1371/journal.pone.0139957 PMC4604148

[embj2021110398-bib-0036] Hara T , Nakamura K , Matsui M , Yamamoto A , Nakahara Y , Suzuki‐Migishima R , Yokoyama M , Mishima K , Saito I , Okano H *et al* (2006) Suppression of basal autophagy in neural cells causes neurodegenerative disease in mice. Nature 441: 885–889 16625204 10.1038/nature04724

[embj2021110398-bib-0037] Hong Z , Pedersen NM , Wang L , Torgersen ML , Stenmark H , Raiborg C (2017) PtdIns3P controls mTORC1 signaling through lysosomal positioning. J Cell Biol 216: 4217–4233 29030394 10.1083/jcb.201611073 PMC5716264

[embj2021110398-bib-0038] Ikonomov OC , Sbrissa D , Venkatareddy M , Tisdale E , Garg P , Shisheva A (2015) Class III PI 3‐kinase is the main source of PtdIns3P substrate and membrane recruitment signal for PIKfyve constitutive function in podocyte endomembrane homeostasis. Biochim Biophys Acta 1853: 1240–1250 25619930 10.1016/j.bbamcr.2015.01.008 PMC4431544

[embj2021110398-bib-0039] Inoki K , Li Y , Zhu T , Wu J , Guan KL (2002) TSC2 is phosphorylated and inhibited by Akt and suppresses mTOR signalling. Nat Cell Biol 4: 648–657 12172553 10.1038/ncb839

[embj2021110398-bib-0040] Ivetac I , Munday AD , Kisseleva MV , Zhang X‐M , Luff S , Tiganis T , Whisstock JC , Rowe T , Majerus PW , Mitchell CA (2005) The type Iα inositol polyphosphate 4‐phosphatase generates and terminates phosphoinositide 3‐kinase signals on endosomes and the plasma membrane. Mol Biol Cell 16: 2218–2233 15716355 10.1091/mbc.E04-09-0799 PMC1087230

[embj2021110398-bib-0041] James SR , Downes CP , Gigg R , Grove SJ , Holmes AB , Alessi DR (1996) Specific binding of the Akt‐1 protein kinase to phosphatidylinositol 3,4,5‐trisphosphate without subsequent activation. Biochem J 315: 709–713 8645147 10.1042/bj3150709 PMC1217264

[embj2021110398-bib-0042] Jefferies HB , Cooke FT , Jat P , Boucheron C , Koizumi T , Hayakawa M , Kaizawa H , Ohishi T , Workman P , Waterfield MD *et al* (2008) A selective PIKfyve inhibitor blocks PtdIns(3,5)P(2) production and disrupts endomembrane transport and retroviral budding. EMBO Rep 9: 164–170 18188180 10.1038/sj.embor.7401155 PMC2246419

[embj2021110398-bib-0043] Klionsky DJ , Petroni G , Amaravadi RK , Baehrecke EH , Ballabio A , Boya P , Bravo‐San Pedro JM , Cadwell K , Cecconi F , Choi AMK *et al* (2021) Autophagy in major human diseases. EMBO J 40: e108863 34459017 10.15252/embj.2021108863 PMC8488577

[embj2021110398-bib-0044] Komatsu M , Waguri S , Chiba T , Murata S , Iwata J , Tanida I , Ueno T , Koike M , Uchiyama Y , Kominami E *et al* (2006) Loss of autophagy in the central nervous system causes neurodegeneration in mice. Nature 441: 880–884 16625205 10.1038/nature04723

[embj2021110398-bib-0045] Kumar S , Javed R , Mudd M , Pallikkuth S , Lidke KA , Jain A , Tangavelou K , Gudmundsson SR , Ye C , Rusten TE *et al* (2021) Mammalian hybrid pre‐autophagosomal structure HyPAS generates autophagosomes. Cell 184: 5950–5969 34741801 10.1016/j.cell.2021.10.017 PMC8616855

[embj2021110398-bib-0046] Lazarou M , Sliter DA , Kane LA , Sarraf SA , Wang C , Burman JL , Sideris DP , Fogel AI , Youle RJ (2015) The ubiquitin kinase PINK1 recruits autophagy receptors to induce mitophagy. Nature 524: 309–314 26266977 10.1038/nature14893 PMC5018156

[embj2021110398-bib-0047] Lees JA , Li P , Kumar N , Weisman LS , Reinisch KM (2020) Insights into lysosomal PI(3,5)P(2) homeostasis from a structural‐biochemical analysis of the PIKfyve lipid kinase complex. Mol Cell 80: 736–743 33098764 10.1016/j.molcel.2020.10.003 PMC7962480

[embj2021110398-bib-0048] Li Chew C , Lunardi A , Gulluni F , Ruan DT , Chen M , Salmena L , Nishino M , Papa A , Ng C , Fung J *et al* (2015) In vivo role of INPP4B in tumor and metastasis suppression through regulation of PI3K‐AKT signaling at endosomes. Cancer Discov 5: 740–751 25883022 10.1158/2159-8290.CD-14-1347 PMC4497843

[embj2021110398-bib-0049] Liu H , Paddock MN , Wang H , Murphy CJ , Geck RC , Navarro AJ , Wulf GM , Elemento O , Haucke V , Cantley LC *et al* (2020) The INPP4B tumor suppressor modulates EGFR trafficking and promotes triple negative breast cancer. Cancer Discov 10: 1226–1239 32513774 10.1158/2159-8290.CD-19-1262 PMC7415683

[embj2021110398-bib-0050] Liu SL , Wang ZG , Hu Y , Xin Y , Singaram I , Gorai S , Zhou X , Shim Y , Min JH , Gong LW *et al* (2018) Quantitative lipid imaging reveals a new signaling function of phosphatidylinositol‐3,4‐bisphophate: isoform‐ and site‐specific activation of Akt. Mol Cell 71: 1092–1104 30174291 10.1016/j.molcel.2018.07.035 PMC6214670

[embj2021110398-bib-0051] Longatti A , Lamb CA , Razi M , Yoshimura S , Barr FA , Tooze SA (2012) TBC1D14 regulates autophagosome formation via Rab11‐ and ULK1‐positive recycling endosomes. J Cell Biol 197: 659–675 22613832 10.1083/jcb.201111079 PMC3365497

[embj2021110398-bib-0052] Ma K , Cheung SM , Marshall AJ , Duronio V (2008) PI(3,4,5)P3 and PI(3,4)P2 levels correlate with PKB/akt phosphorylation at Thr308 and Ser473, respectively; PI(3,4)P2 levels determine PKB activity. Cell Signal 20: 684–694 18249092 10.1016/j.cellsig.2007.12.004

[embj2021110398-bib-0053] Malerød L , Stuffers S , Brech A , Stenmark H (2007) Vps22/EAP30 in ESCRT‐II mediates endosomal sorting of growth factor and chemokine receptors destined for lysosomal degradation. Traffic 8: 1617–1629 17714434 10.1111/j.1600-0854.2007.00630.x

[embj2021110398-bib-0054] Manning BD , Toker A (2017) AKT/PKB signaling: navigating the network. Cell 169: 381–405 28431241 10.1016/j.cell.2017.04.001 PMC5546324

[embj2021110398-bib-0055] Marat AL , Wallroth A , Lo WT , Muller R , Norata GD , Falasca M , Schultz C , Haucke V (2017) mTORC1 activity repression by late endosomal phosphatidylinositol 3,4‐bisphosphate. Science 356: 968–972 28572395 10.1126/science.aaf8310

[embj2021110398-bib-0056] Martin S , Harper CB , May LM , Coulson EJ , Meunier FA , Osborne SL (2013) Inhibition of PIKfyve by YM‐201636 dysregulates autophagy and leads to apoptosis‐independent neuronal cell death. PLoS ONE 8: e60152 23544129 10.1371/journal.pone.0060152 PMC3609765

[embj2021110398-bib-0057] Mauvezin C , Nagy P , Juhász G , Neufeld TP (2015) Autophagosome‐lysosome fusion is independent of V‐ATPase‐mediated acidification. Nat Commun 6: 7007 25959678 10.1038/ncomms8007 PMC4428688

[embj2021110398-bib-0058] McGrath MJ , Eramo MJ , Gurung R , Sriratana A , Gehrig SM , Lynch GS , Lourdes SR , Koentgen F , Feeney SJ , Lazarou M *et al* (2021) Defective lysosome reformation during autophagy causes skeletal muscle disease. J Clin Invest 131: e135124 10.1172/JCI135124 PMC7773396 33119550

[embj2021110398-bib-0059] Mellado M , Cuartero Y , Brugada R , Verges M (2014) Subcellular localisation of retromer in post‐endocytic pathways of polarised Madin‐Darby canine kidney cells. Biol Cell 106: 377–393 25081925 10.1111/boc.201400011

[embj2021110398-bib-0060] Miller A , Schafer J , Upchurch C , Spooner E , Huynh J , Hernandez S , McLaughlin B , Oden L , Fares H (2015) Mucolipidosis type IV protein TRPML1‐dependent lysosome formation. Traffic 16: 284–297 25491304 10.1111/tra.12249

[embj2021110398-bib-0061] Mizushima N , Yoshimori T (2007) How to interpret LC3 immunoblotting. Autophagy 3: 542–545 17611390 10.4161/auto.4600

[embj2021110398-bib-0062] Munson MJ , Allen GF , Toth R , Campbell DG , Lucocq JM , Ganley IG (2015) mTOR activates the VPS34‐UVRAG complex to regulate autolysosomal tubulation and cell survival. EMBO J 34: 2272–2290 26139536 10.15252/embj.201590992 PMC4585463

[embj2021110398-bib-0063] N'Diaye EN , Kajihara KK , Hsieh I , Morisaki H , Debnath J , Brown EJ (2009) PLIC proteins or ubiquilins regulate autophagy‐dependent cell survival during nutrient starvation. EMBO Rep 10: 173–179 19148225 10.1038/embor.2008.238 PMC2637314

[embj2021110398-bib-0064] Nakamura S , Yoshimori T (2018) Autophagy and longevity. Mol Cells 41: 65–72 29370695 10.14348/molcells.2018.2333 PMC5792715

[embj2021110398-bib-0065] Naughtin MJ , Sheffield DA , Rahman P , Hughes WE , Gurung R , Stow JL , Nandurkar HH , Dyson JM , Mitchell CA (2010) The myotubularin phosphatase MTMR4 regulates sorting from early endosomes. J Cell Sci 123: 3071–3083 20736309 10.1242/jcs.060103

[embj2021110398-bib-0066] Nobukuni T , Joaquin M , Roccio M , Dann SG , Kim SY , Gulati P , Byfield MP , Backer JM , Natt F , Bos JL *et al* (2005) Amino acids mediate mTOR/raptor signaling through activation of class 3 phosphatidylinositol 3OH‐kinase. Proc Natl Acad Sci USA 102: 14238–14243 16176982 10.1073/pnas.0506925102 PMC1242323

[embj2021110398-bib-0067] Oorschot V , Lindsey BW , Kaslin J , Ramm G (2021) TEM, SEM, and STEM‐based immuno‐CLEM workflows offer complementary advantages. Sci Rep 11: 899 33441723 10.1038/s41598-020-79637-9 PMC7806999

[embj2021110398-bib-0068] Otsu N (1979) A threshold selection method from gray level histograms. IEEE Trans Syst Man Cybern 9: 62–66

[embj2021110398-bib-0069] Padman BS , Nguyen TN , Uoselis L , Skulsuppaisarn M , Nguyen LK , Lazarou M (2019) LC3/GABARAPs drive ubiquitin‐independent recruitment of optineurin and NDP52 to amplify mitophagy. Nat Commun 10: 408 30679426 10.1038/s41467-019-08335-6 PMC6345886

[embj2021110398-bib-0070] Park J , Park Y , Ryu I , Choi MH , Lee HJ , Oh N , Kim K , Kim KM , Choe J , Lee C *et al* (2017) Misfolded polypeptides are selectively recognized and transported toward aggresomes by a CED complex. Nat Commun 8: 15730 28589942 10.1038/ncomms15730 PMC5467238

[embj2021110398-bib-0071] Polson HE , de Lartigue J , Rigden DJ , Reedijk M , Urbé S , Clague MJ , Tooze SA (2010) Mammalian Atg18 (WIPI2) localizes to omegasome‐anchored phagophores and positively regulates LC3 lipidation. Autophagy 6: 506–522 20505359 10.4161/auto.6.4.11863

[embj2021110398-bib-0072] Pryor PR , Mullock BM , Bright NA , Gray SR , Luzio JP (2000) The role of intraorganellar ca(2+) in late endosome‐lysosome heterotypic fusion and in the reformation of lysosomes from hybrid organelles. J Cell Biol 149: 1053–1062 10831609 10.1083/jcb.149.5.1053 PMC2174832

[embj2021110398-bib-0073] Puri C , Vicinanza M , Ashkenazi A , Gratian MJ , Zhang Q , Bento CF , Renna M , Menzies FM , Rubinsztein DC (2018) The RAB11A‐positive compartment is a primary platform for autophagosome assembly mediated by WIPI2 recognition of PI3P‐RAB11A. Dev Cell 45: 114–131 29634932 10.1016/j.devcel.2018.03.008 PMC5896254

[embj2021110398-bib-0074] Qiu S , Lavallée‐Adam M , Côté M (2021) Proximity interactome map of the Vac14‐Fig4 complex using BioID. J Proteome Res 20: 4959–4973 34554760 10.1021/acs.jproteome.1c00408

[embj2021110398-bib-0075] Rijal S , Fleming S , Cummings N , Rynkiewicz NK , Ooms LM , Nguyen NY , Teh TC , Avery S , McManus JF , Papenfuss AT *et al* (2015) Inositol polyphosphate 4‐phosphatase II (INPP4B) is associated with chemoresistance and poor outcome in AML. Blood 125: 2815–2824 25736313 10.1182/blood-2014-09-603555

[embj2021110398-bib-0076] Rodgers SJ , Ferguson DT , Mitchell CA , Ooms LM (2017) Regulation of PI3K effector signalling in cancer by the phosphoinositide phosphatases. Biosci Rep 37: BSR20160432 28082369 10.1042/BSR20160432 PMC5301276

[embj2021110398-bib-0077] Rodgers SJ , Ooms LM , Oorschot VMJ , Schittenhelm RB , Nguyen EV , Hamila SA , Rynkiewicz N , Gurung R , Eramo MJ , Sriratana A *et al* (2021) INPP4B promotes PI3Kα‐dependent late endosome formation and Wnt/β‐catenin signaling in breast cancer. Nat Commun 12: 3140 34035258 10.1038/s41467-021-23241-6 PMC8149851

[embj2021110398-bib-0078] Ronan B , Flamand O , Vescovi L , Dureuil C , Durand L , Fassy F , Bachelot MF , Lamberton A , Mathieu M , Bertrand T *et al* (2014) A highly potent and selective Vps34 inhibitor alters vesicle trafficking and autophagy. Nat Chem Biol 10: 1013–1019 25326666 10.1038/nchembio.1681

[embj2021110398-bib-0079] Rong Y , Liu M , Ma L , Du W , Zhang H , Tian Y , Cao Z , Li Y , Ren H , Zhang C *et al* (2012) Clathrin and phosphatidylinositol‐4,5‐bisphosphate regulate autophagic lysosome reformation. Nat Cell Biol 14: 924–934 22885770 10.1038/ncb2557

[embj2021110398-bib-0080] Russell RC , Tian Y , Yuan H , Park HW , Chang Y‐Y , Kim J , Kim H , Neufeld TP , Dillin A , Guan K‐L (2013) ULK1 induces autophagy by phosphorylating Beclin‐1 and activating VPS34 lipid kinase. Nat Cell Biol 15: 741–750 23685627 10.1038/ncb2757 PMC3885611

[embj2021110398-bib-0081] Rusten TE , Vaccari T , Lindmo K , Rodahl LM , Nezis IP , Sem‐Jacobsen C , Wendler F , Vincent JP , Brech A , Bilder D *et al* (2007) ESCRTs and Fab1 regulate distinct steps of autophagy. Curr Biol 17: 1817–1825 17935992 10.1016/j.cub.2007.09.032

[embj2021110398-bib-0082] Rutherford AC , Traer C , Wassmer T , Pattni K , Bujny MV , Carlton JG , Stenmark H , Cullen PJ (2006) The mammalian phosphatidylinositol 3‐phosphate 5‐kinase (PIKfyve) regulates endosome‐to‐TGN retrograde transport. J Cell Sci 119: 3944–3957 16954148 10.1242/jcs.03153 PMC1904490

[embj2021110398-bib-0083] Scheffler JM , Schiefermeier N , Huber LA (2014) Mild fixation and permeabilization protocol for preserving structures of endosomes, focal adhesions, and Actin filaments during immunofluorescence analysis. Methods Enzymol 535: 93–102 24377919 10.1016/B978-0-12-397925-4.00006-7

[embj2021110398-bib-0084] Schneider CA , Rasband WS , Eliceiri KW (2012) NIH image to ImageJ: 25 years of image analysis. Nat Methods 9: 671–675 22930834 10.1038/nmeth.2089 PMC5554542

[embj2021110398-bib-0085] Settembre C , Di Malta C , Polito VA , Garcia Arencibia M , Vetrini F , Erdin S , Erdin SU , Huynh T , Medina D , Colella P *et al* (2011) TFEB links autophagy to lysosomal biogenesis. Science 332: 1429–1433 21617040 10.1126/science.1204592 PMC3638014

[embj2021110398-bib-0086] Settembre C , Zoncu R , Medina DL , Vetrini F , Erdin S , Erdin S , Huynh T , Ferron M , Karsenty G , Vellard MC *et al* (2012) A lysosome‐to‐nucleus signalling mechanism senses and regulates the lysosome via mTOR and TFEB. EMBO J 31: 1095–1108 22343943 10.1038/emboj.2012.32 PMC3298007

[embj2021110398-bib-0087] Sherer NM , Lehmann MJ , Jimenez‐Soto LF , Ingmundson A , Horner SM , Cicchetti G , Allen PG , Pypaert M , Cunningham JM , Mothes W (2003) Visualization of retroviral replication in living cells reveals budding into multivesicular bodies. Traffic 4: 785–801 14617360 10.1034/j.1600-0854.2003.00135.x

[embj2021110398-bib-0088] Shin H‐W , Hayashi M , Christoforidis S , Lacas‐Gervais S , Hoepfner S , Wenk MR , Modregger J , Uttenweiler‐Joseph S , Wilm M , Nystuen A *et al* (2005) An enzymatic cascade of Rab5 effectors regulates phosphoinositide turnover in the endocytic pathway. J Cell Biol 170: 607–618 16103228 10.1083/jcb.200505128 PMC2171494

[embj2021110398-bib-0089] Slot JW , Geuze HJ (2007) Cryosectioning and immunolabeling. Nat Protoc 2: 2480–2491 17947990 10.1038/nprot.2007.365

[embj2021110398-bib-0090] Urwin H , Authier A , Nielsen JE , Metcalf D , Powell C , Froud K , Malcolm DS , Holm I , Johannsen P , Brown J *et al* (2010) Disruption of endocytic trafficking in frontotemporal dementia with CHMP2B mutations. Hum Mol Genet 19: 2228–2238 20223751 10.1093/hmg/ddq100 PMC2865375

[embj2021110398-bib-0091] van Weering JR , Sessions RB , Traer CJ , Kloer DP , Bhatia VK , Stamou D , Carlsson SR , Hurley JH , Cullen PJ (2012) Molecular basis for SNX‐BAR‐mediated assembly of distinct endosomal sorting tubules. EMBO J 31: 4466–4480 23085988 10.1038/emboj.2012.283 PMC3512392

[embj2021110398-bib-0092] Vantaggiato C , Panzeri E , Castelli M , Citterio A , Arnoldi A , Santorelli FM , Liguori R , Scarlato M , Musumeci O , Toscano A *et al* (2019) ZFYVE26/SPASTIZIN and SPG11/SPATACSIN mutations in hereditary spastic paraplegia types AR‐SPG15 and AR‐SPG11 have different effects on autophagy and endocytosis. Autophagy 15: 34–57 30081747 10.1080/15548627.2018.1507438 PMC6287682

[embj2021110398-bib-0093] Varga R‐E , Khundadze M , Damme M , Nietzsche S , Hoffmann B , Stauber T , Koch N , Hennings JC , Franzka P , Huebner AK *et al* (2015) In vivo evidence for lysosome depletion and impaired autophagic clearance in hereditary spastic paraplegia type SPG11. PLoS Genet 11: e1005454 26284655 10.1371/journal.pgen.1005454 PMC4540459

[embj2021110398-bib-0094] Yu L , McPhee CK , Zheng L , Mardones GA , Rong Y , Peng J , Mi N , Zhao Y , Liu Z , Wan F *et al* (2010) Termination of autophagy and reformation of lysosomes regulated by mTOR. Nature 465: 942–946 20526321 10.1038/nature09076 PMC2920749

[embj2021110398-bib-0095] Yu X , Long YC , Shen HM (2015) Differential regulatory functions of three classes of phosphatidylinositol and phosphoinositide 3‐kinases in autophagy. Autophagy 11: 1711–1728 26018563 10.1080/15548627.2015.1043076 PMC4824607

[embj2021110398-bib-0096] Zhang X , Chow CY , Sahenk Z , Shy ME , Meisler MH , Li J (2008) Mutation of FIG4 causes a rapidly progressive, asymmetric neuronal degeneration. Brain 131: 1990–2001 18556664 10.1093/brain/awn114 PMC2724900

[embj2021110398-bib-0097] Zhang Y , Zolov SN , Chow CY , Slutsky SG , Richardson SC , Piper RC , Yang B , Nau JJ , Westrick RJ , Morrison SJ *et al* (2007) Loss of Vac14, a regulator of the signaling lipid phosphatidylinositol 3,5‐bisphosphate, results in neurodegeneration in mice. Proc Natl Acad Sci USA 104: 17518–17523 17956977 10.1073/pnas.0702275104 PMC2077288

[embj2021110398-bib-0098] Zhao YG , Codogno P , Zhang H (2021) Machinery, regulation and pathophysiological implications of autophagosome maturation. Nat Rev Mol Cell Biol 22: 733–750 34302147 10.1038/s41580-021-00392-4 PMC8300085

[embj2021110398-bib-0099] Zolov SN , Bridges D , Zhang Y , Lee WW , Riehle E , Verma R , Lenk GM , Converso‐Baran K , Weide T , Albin RL *et al* (2012) In vivo, Pikfyve generates PI(3,5)P2, which serves as both a signaling lipid and the major precursor for PI5P. Proc Natl Acad Sci USA 109: 17472–17477 23047693 10.1073/pnas.1203106109 PMC3491506

