# Supplementary material for: Endosome maturation links PI3Kα signaling to lysosome repopulation during basal autophagy
Source: EMBO J. 2022 Aug 15;41(19):e110398. doi: 10.15252/embj.2021110398 (PMC9531306; doi:10.15252/embj.2021110398)
Supplement: Supplementary file 1 — Appendix [file EMBJ-41-e110398-s001.pdf]

Appendix

Endosome maturation links PI3Kα signaling to lysosome repopulation during basal autophagy

Samuel J. Rodgers, Emily I. Jones, Senthil Arumugam, Sabryn A. Hamila, Jill Danne, Rajendra Gurung, Matthew J. Eramo, Randini Nanayakkara, Georg Ramm, Meagan J. McGrath, Christina A. Mitchell

Table of contents

Appendix Figure S1: INPP4B promotes basal autophagic flux…………………… **Page 1**

Appendix Figure S2: Rapid imaging and analysis workflow used to quantify lysosome reformation……………………………………………………………… **Page 4**

Appendix Figure S3: Validation of SNX2 siRNA depletion……………………… **Page 5**

Appendix Table S1: Antibodies and dyes…………………………………………. **Page 7**

Appendix Table S2: Oligonucleotides……………………………………….......... **Page 9**

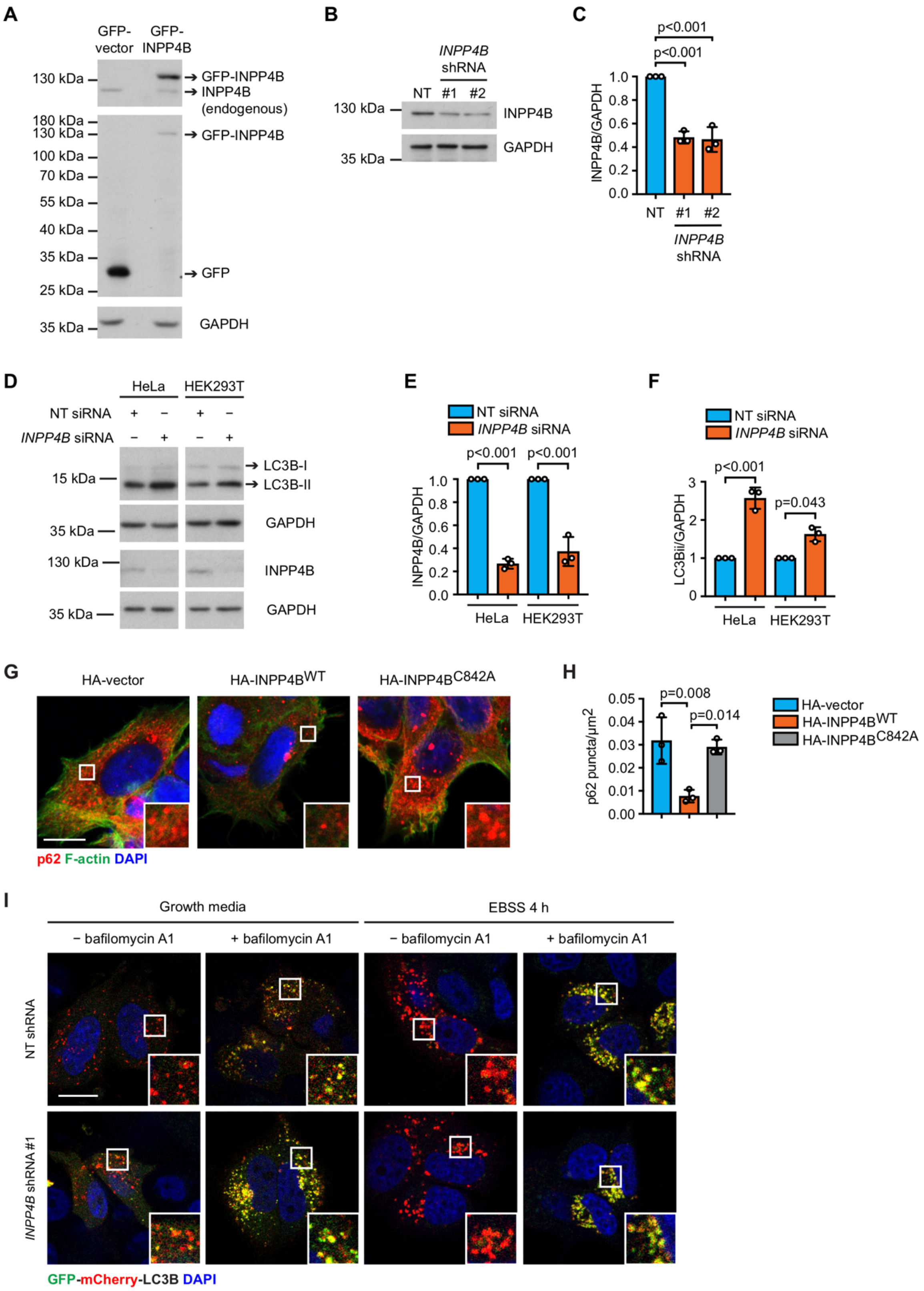
A
GFP-vector
GFP-INPP4B
130 kDa
180 kDa
130 kDa
100 kDa
70 kDa
55 kDa
40 kDa
35 kDa
25 kDa
35 kDa
GFP-INPP4B
INPP4B (endogenous)
GFP-INPP4B
GFP
GAPDH
B
INPP4B shRNA
NT #1 #2
130 kDa
35 kDa
INPP4B
GAPDH
C
p<0.001
p<0.001
INPP4B/GAPDH
NT #1 #2
INPP4B shRNA
D
HeLa
HEK293T
NT siRNA
INPP4B siRNA
15 kDa
35 kDa
130 kDa
35 kDa
LC3B-I
LC3B-II
GAPDH
INPP4B
GAPDH
E
NT siRNA
INPP4B siRNA
p<0.001 p<0.001
INPP4B/GAPDH
HeLa HEK293T
F
NT siRNA
INPP4B siRNA
p<0.001
p=0.043
LC3Bii/GAPDH
HeLa HEK293T
G
HA-vector
HA-INPP4BWT
HA-INPP4BC842A
p62 F-actin DAPI
H
p=0.008
p=0.014
p62 puncta/µm2
HA-vector
HA-INPP4BWT
HA-INPP4BC842A
I
Growth media
EBSS 4 h
− bafilomycin A1
+ bafilomycin A1
− bafilomycin A1
+ bafilomycin A1
NT shRNA
INPP4B shRNA #1
GFP-mCherry-LC3B DAPI

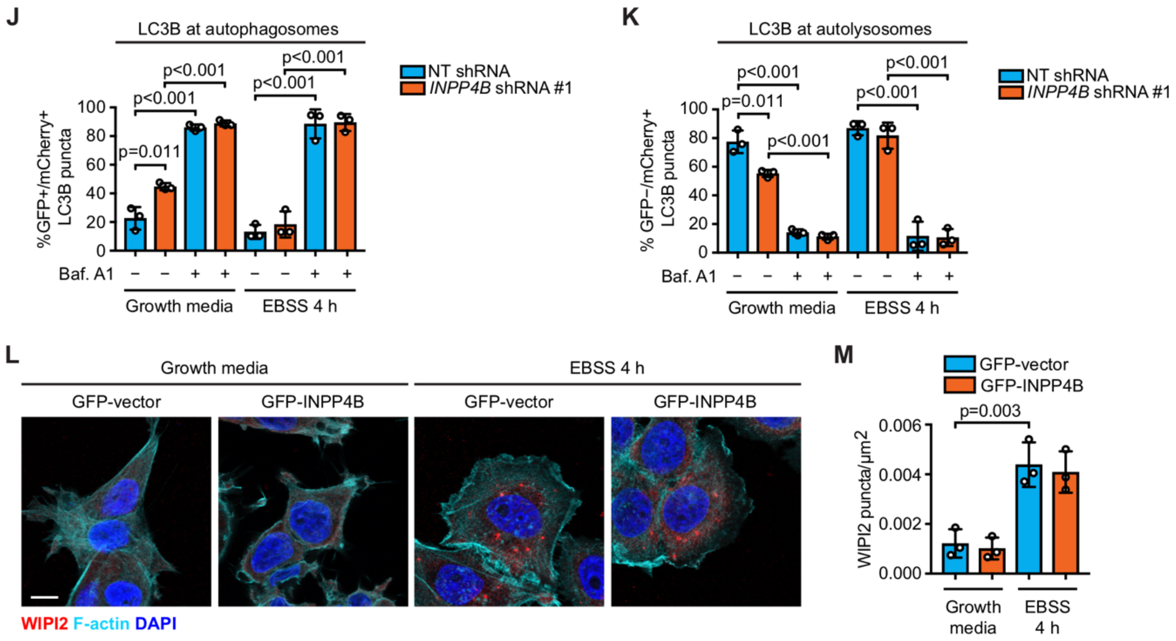

Appendix Figure S1: INPP4B promotes basal autophagic flux

A MCF-7 cells were transduced with lentiviral particles encoding GFP-INPP4B or GFP-vector. Cells were lysed and immunoblotted with INPP4B and GFP antibodies, and GAPDH antibodies as a loading control.

B, C MCF-7 cells were transduced with lentiviral particles encoding NT, *INPP4B* #1 or *INPP4B* #2 shRNA. Cells were lysed and immunoblotted with INPP4B antibodies, and GAPDH antibodies as a loading control (**B**). Data represent the relative INPP4B levels normalized to GAPDH, and expressed relative to NT shRNA cells which were assigned an arbitrary value of 1 (n=3 experiments) (**C**).

D-F HeLa or HEK293T cells were transfected with NT or *INPP4B* siRNA. After 24 hours, cells were lysed and immunoblotted with LC3B and INPP4B antibodies, and GAPDH antibodies as a loading control (**D**). Data represent the relative INPP4B (**E**) or LC3B-II (**F**) levels normalized to GAPDH, and expressed relative to NT siRNA cells which were assigned an arbitrary value of 1 (n=3 experiments).

G, H MCF-7 cells were transfected with HA-vector, HA-INPP4B^{WT} or HA-INPP4B^{C842A}. After 24 hours, cells were fixed and immunostained with p62 antibodies, and co-stained with DAPI and phalloidin (**G**). Data represent the number of p62+ puncta relative to cell area (μm^2) (n=3 experiments, >20 cells/experiment) (**H**).

I-K MCF-7 cells co-expressing GFP-mCherry-LC3B and NT or *INPP4B* #1 shRNA were cultured in growth media or EBSS in the presence of 100 nM bafilomycin A1 or DMSO as a vehicle control for 4 hours, then fixed and stained with DAPI (**I**). Data represent the proportion of LC3B at autophagosomes (GFP+/mCherry+) (**J**) or autolysosomes (GFP−/mCherry+) (**K**) (n=3 experiments, >30 cells/experiment).

L, M MCF-7 cells expressing GFP-INPP4B or GFP-vector were cultured in growth media or EBSS for 4 hours, then fixed and immunostained with WIPI2 antibodies, and co-stained

with DAPI and phalloidin (**L**). Data represent the number of WIPI2+ puncta relative to cell area (μm^2) (n=3 experiments, >50 cells/experiment) (**M**).

Data information: Data is presented as mean ± SD. The insets at the lower right of each image are higher power regions of the boxed areas. Scale bar is 10 µm in **G**, **I**, **L**. p values determined by one-way ANOVA with Tukey post hoc test in **C**, **H**, **J**, **K**, **M**, or by two-tailed unpaired t test in **E**, **F**.

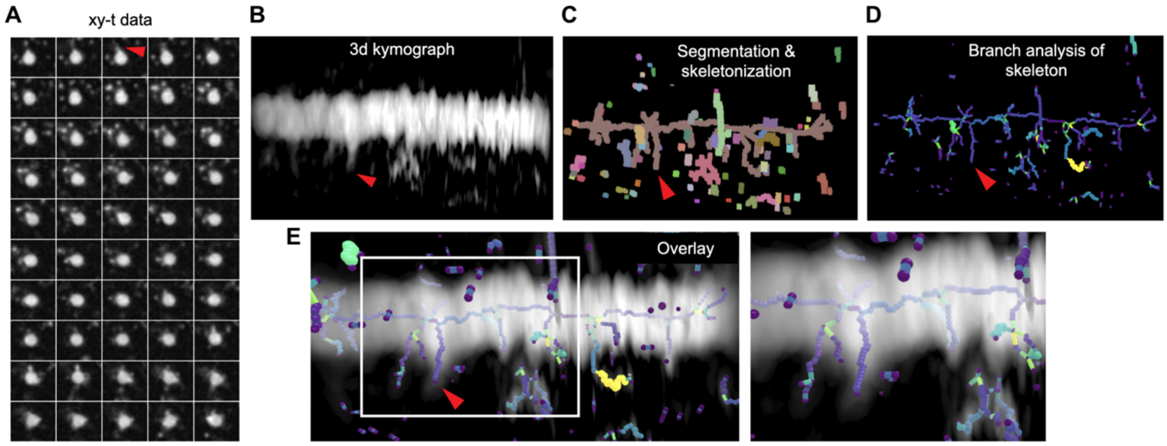

Appendix Figure S2: Rapid imaging and analysis workflow used to quantify lysosome reformation

A-E Spinning disk movies of LAMP1-mCherry signals were projected as maximum intensity from 3 z-planes (**A**). 3-dimensional kymographs were constructed from *x*, *y* and time dimensions (**B**), then subjected to segmentation and skeletonization (**C**). Branch points from the parent lysosome indicate lysosome reformation (**D**). Overlay between 3-dimensional kymographs and skeletons (**E**). Lysosome reformation event is indicated with red arrows. The inset at the bottom right is a higher power region of the boxed area.

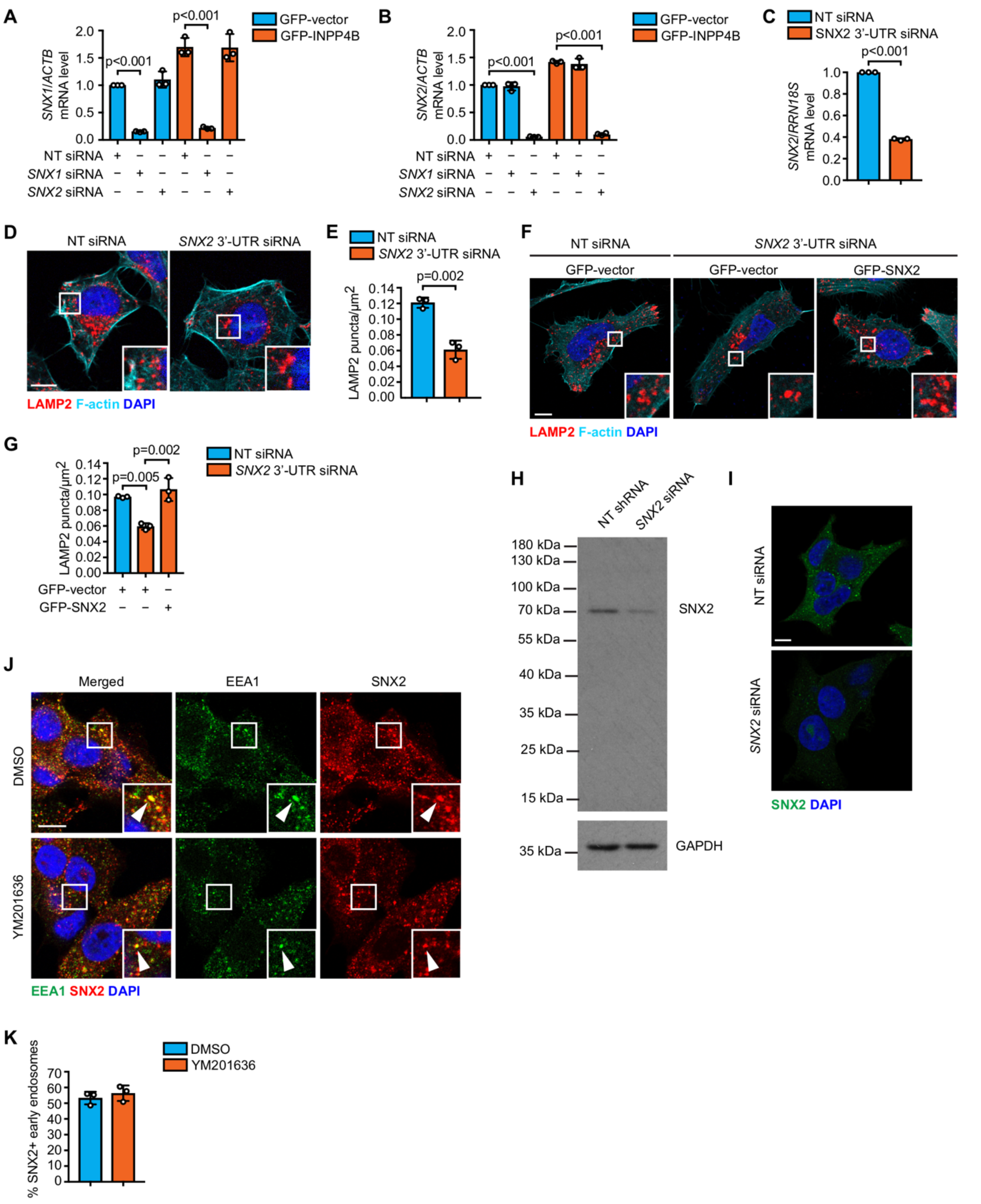

Appendix Figure S3: Validation of SNX2 siRNA depletion

A, B MCF-7 cells expressing GFP-INPP4B or GFP-vector were transfected with NT, *SNX1* or *SNX2* siRNA. After 24 hours, RNA was extracted and two-step quantitative RT-PCR was performed using primers for *SNX1* (**A**) or *SNX2* (**B**), and expression was normalized to *ACTB*. Expression was determined using the ΔΔCt method and expressed relative to GFP-vector;NT siRNA cells which were assigned an arbitrary value of 1 (n=3 experiments).

C HeLa cells were transfected with NT or *SNX2* siRNA. After 24 hours, RNA was extracted and two-step quantitative RT-PCR was performed using primers for *SNX2*, and expression was normalized to *RRN18S*. Expression was determined using the ΔΔCt method and expressed relative to NT siRNA cells which were assigned an arbitrary value of 1 (n=3 experiments).

D, E HeLa cells were co-transfected with NT or *SNX2* 3'-UTR siRNA. Cells were fixed and immunostained with LAMP2 antibodies, and co-stained with DAPI and phalloidin (**D**). Data represent the number of LAMP2+ puncta relative to cell area (μm^2) (n=3 experiments, >50 cells per experiment) (**E**).

F, G HeLa cells were co-transfected with NT or *SNX2* 3'-UTR siRNA, and GFP-vector or GFP-SNX2. Cells were fixed and immunostained with LAMP2 antibodies, and co-stained with DAPI and phalloidin (**F**). Data represent the number of LAMP2+ puncta relative to cell area (μm^2) (n=3 experiments, >20 cells per experiment) (**G**).

H MCF-7 cells were transfected with NT or *SNX2* siRNA, then lysed and immunoblotted with SNX2 antibodies, and GAPDH antibodies as a loading control.

I MCF-7 cells were transfected with NT or *SNX2* #1 siRNA, then fixed and immunostained with SNX2 antibodies, and co-stained with DAPI.

J, K MCF-7 cells were treated with 5 μM YM201636 or DMSO as a vehicle control for 4 hours. Cells were fixed and immunostained with SNX2 and EEA1 antibodies, and co-stained with DAPI (**J**). Data represent the percentage of SNX2+ early endosomes (n=3 experiments, >50 cells/experiment) (**K**).

Data information: Data is presented as mean ± SD. The insets at the bottom of each image are higher power regions of the boxed areas. Scale bar is 10 μm in **D**, **F**, **I**, **J**. p values determined by one-way ANOVA with Tukey post hoc test in **A**, **B**, **G**, or by two-tailed unpaired t test in **C**, **E**.

Appendix Table S1: Antibodies and dyes

AKT(pan) (IB – 1:1000)	Cell Signaling Technologies	Cat # 4691
CD63 (IF – 1:200)	DSHB	Cat # H5C6
GAPDH (IB – 1:500,000)	ThermoFisher Scientific	Cat # AM4300
GFP (IB – 1:50,000)	Roche	Cat # 11814460001
GFP (IF – 1:500)	Invitrogen	Cat # A10262
GFP (IEM – 1:500)	Abcam	Cat # ab6556
GST (IF - 1:500)	Invitrogen	Cat # 71-7500
HA (IB – 1:1000, IF – 1:1600)	Cell Signaling Technologies	Cat # 3724
Hrs (IB – 1:1000)	Cell Signaling Technologies	Cat # 15087
INPP4B 3D5 (IB – 1:1000, IF – 1:50)	(Fedele et al., 2010)	N/A
LAMP1 (IF – 1:100)	DSHB	Cat # G1/139/5
LAMP2 (IF – 1:100)	DSHB	Cat # H4B4
LC3B (IB – 1:1000)	Cell Signaling Technologies	Cat # 2775
Phospho-AKT^{S473} (IB - 1:1000)	Cell Signaling Technologies	Cat # 4058
Phospho-mTOR^{S2448} (IF – 1:100)	Cell Signaling Technologies	Cat # 2971
Phospho-S6K^{T389} (IB – 1:2000)	Cell Signaling Technologies	Cat # 9234
S6K (IB – 1:2000)	Cell Signaling Technologies	Cat # 9202
SNX2 (IB – 1:2000, IF – 1:100)	Invitrogen	Cat # PA5-83367
SQSTM1/p62 (IF – 1:500)	Abcam	Cat # ab56416
Ubiquitin (IF – 1:500)	Enzo Life Sciences	Cat # BML-PW8810-0500
WIPI2 (IF – 1:500)	Abcam	Cat # ab105459
Anti-mouse HRP-conjugated (IB – 1:10,000)	Millipore	Cat # AP308P
Anti-rabbit HRP-conjugated (IB – 1:10,000)	Millipore	Cat # AP307P
Donkey anti-mouse IgG (H+L) Alexa-Fluor 488-conjugated (IF – 1:500)	Life Technologies	Cat # A-21202
Donkey anti-mouse IgG (H+L) Alexa-Fluor 555-conjugated (IF – 1:500)	Life Technologies	Cat # A-31570

Goat anti-mouse IgG1 Alexa-Fluor 555-conjugated (IF – 1:500)	Life Technologies	Cat # A-21127
Goat anti-mouse IgG1 Alexa-Fluor 647-conjugated (IF – 1:500)	Life Technologies	Cat # A-21240
Goat anti-mouse IgG2a Alexa-Fluor 488-conjugated (IF – 1:500)	Life Technologies	Cat # A-21131
Donkey anti-rabbit IgG (H+L) Alexa-Fluor 488-conjugated (IF – 1:500)	Life Technologies	Cat # A-21206
Donkey anti-rabbit IgG (H+L) Alexa-Fluor 555-conjugated (IF – 1:500)	Life Technologies	Cat # A-31572
DAPI (IF – 1:1000)	Sigma	Cat # D9542
Phalloidin Alexa-Fluor 647-conjugated (IF – 1:500)	Life Technologies	Cat # A22287

Appendix Table S2: Oligonucleotides

Human *ATCB* (qRT-PCR)	Qiagen	QuantiTect GeneGlobe ID # QT00095431
Human *ATP6V1C1* (qRT-PCR)	Qiagen	QuantiTect GeneGlobe ID # QT00015022
Human *ATP6V0D1* (qRT-PCR)	Qiagen	QuantiTect GeneGlobe ID # QT00018116
Human *CTNS* (qRT-PCR)	Qiagen	QuantiTect GeneGlobe ID # QT00046914
Human *LAMP1* (qRT-PCR)	Qiagen	QuantiTect GeneGlobe ID # QT00070994
Human *M6PR* (qRT-PCR)	Qiagen	QuantiTect GeneGlobe ID # QT00026894
Human *PIKFYVE* (qRT-PCR)	Qiagen	QuantiTect GeneGlobe ID # QT00035231
Human *RRN18S* (qRT-PCR)	Qiagen	QuantiTect GeneGlobe ID # QT00199367
Human *SNX1* (qRT-PCR)	Sigma	KiCqStart primer pair #1
Human *SNX2* (qRT-PCR)	Qiagen	QuantiTect GeneGlobe ID # QT02307648
Human *TPP1* (qRT-PCR)	Qiagen	QuantiTect GeneGlobe ID # QT00097363
