# Supplementary material for: Endosome maturation links PI3Kα signaling to lysosome repopulation during basal autophagy
Source: EMBO J. 2022 Aug 15;41(19):e110398. doi: 10.15252/embj.2021110398 (PMC9531306; doi:10.15252/embj.2021110398)
Supplement: Supplementary file 2 — Expanded View Figures PDF [file EMBJ-41-e110398-s011.pdf]

Expanded View Figures

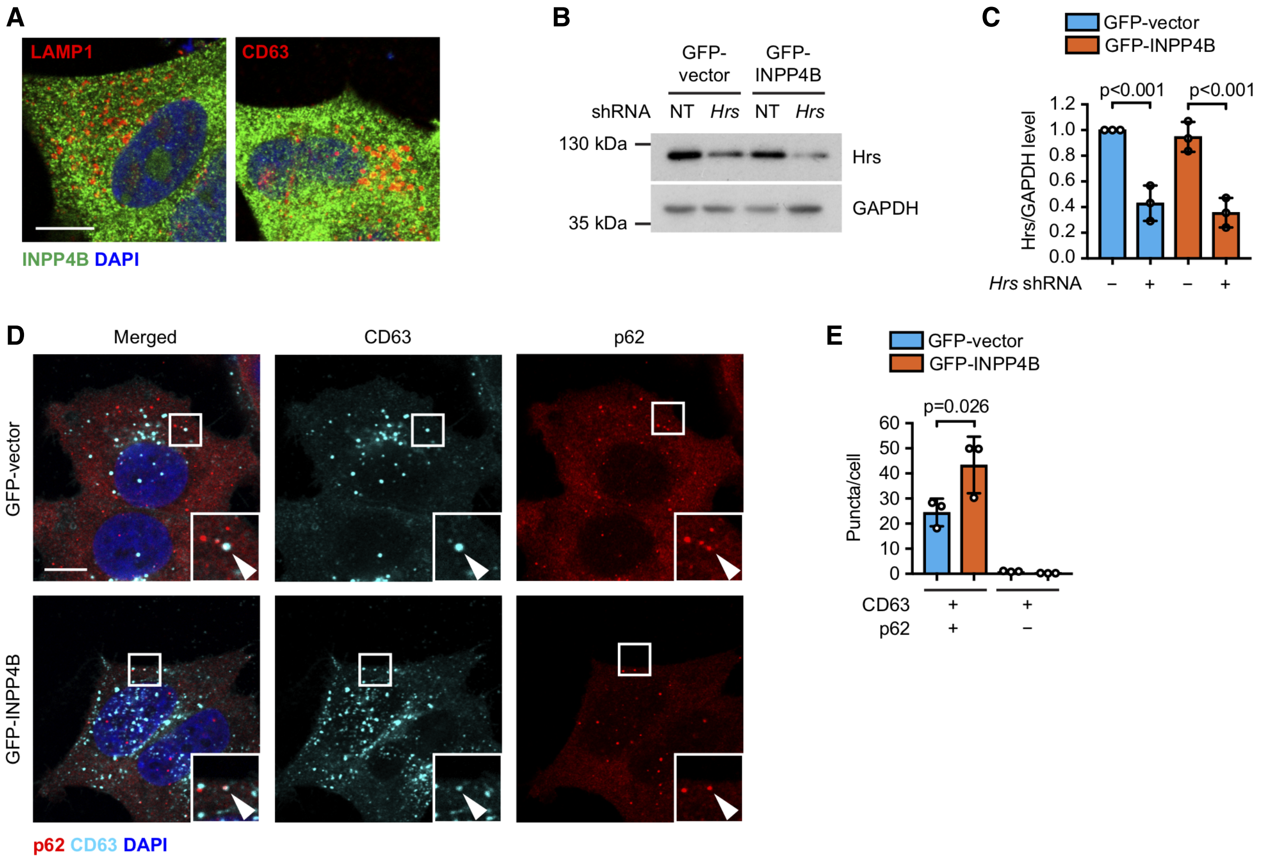

Figure EV1. INPP4B does not affect amphisome formation.

A MCF-7 cells were fixed and immunostained with INPP4B and either LAMP1 or CD63 antibodies, and co-stained with DAPI.

B, C MCF-7 cells expressing GFP-INPP4B or GFP-vector were transduced with lentiviral particles encoding nontargeted (NT) or *Hrs* shRNA. Cells were lysed and immunoblotted with Hrs antibodies, and GAPDH antibodies as a loading control (B). Data represent the relative Hrs levels normalized to GAPDH expression, and expressed relative to GFP-vector;NT shRNA cells which were assigned an arbitrary value of 1 (n = 3 experiments) (C).

D, E MCF-7 cells expressing GFP-INPP4B or GFP-vector were fixed and stained with CD63 and p62 antibodies, and co-stained with DAPI (D). Data represent the number of late endosomes ($CD63^{+}/p62^{-}$ puncta) and amphisomes ($CD63^{+}/p62^{+}$ puncta) per cell (n = 3 experiments, > 50 cells/experiment) (E). Arrows indicate amphisomes.

Data information: Data are presented as mean ± SD. The insets at the lower right of each image are higher power regions of the boxed areas. Scale bar is 10 μm in (A, D). *P* values determined by one-way ANOVA with Tukey *post hoc* test in (C, E).
Source data are available online for this figure.

 © 2022 The Authors

Figure EV2. INPP4B does not affect lysosome size or dispersion.

A, B MCF-7 cells expressing nontargeted (NT), *INPP4B* #1, or *INPP4B* #2 shRNA were fixed and immunostained with LAMP1 antibodies, and co-stained with DAPI and phalloidin (A). Data represent the number of $LAMP1^+$ puncta relative to cell area (μm^2) (n = 3 experiments, > 50 cells/experiment) (B).

C, D HeLa cells were transfected with NT or *INPP4B* siRNA. After 24 h, cells were fixed and immunostained with LAMP2 antibodies, and co-stained with DAPI and phalloidin (C). Data represent the number of $LAMP2^+$ puncta relative to cell area (μm^2) (n = 3 experiments, > 50 cells/experiment) (D).

E, F MCF-7 cells were transfected with HA-vector, HA-$INPP4B,^{WT}$ or HA-$INPP4B^{C842A}$. Forty-eight hours later, cells were fixed and immunostained with LAMP2 antibodies, and co-stained with DAPI and phalloidin (E). Data represent the number of $LAMP2^+$ puncta relative to cell area (μm^2) (n = 3 experiments, > 20 cells/experiment) (F).

G, H MCF-7 cells co-expressing GFP-INPP4B or GFP-vector, and *Hrs* or NT shRNA, were fixed and immunostained with LAMP1 antibodies, and co-stained with DAPI and phalloidin (G). Data represent the number of $LAMP1^+$ puncta relative to cell area (μm^2) (n = 3 experiments, > 40 cells per experiment) (H).

I–K MCF-7 cells expressing GFP-INPP4B or GFP-vector were fixed and immunostained with LAMP1 antibodies, and imaged using super resolution microscopy (I). Data represent the $LAMP1^+$ puncta size (J) and $LAMP1^+$ puncta size distribution (K) (n = 3 experiments, > 20 cells/experiment).

L, M Data represent the distance of $LAMP1^+$ puncta from the center of the nucleus (L), and the proportion of perinuclear $LAMP1^+$ puncta (< 15 μm from center of nucleus) and peripheral $LAMP1^+$ puncta (> 15 μm from center of nucleus) (M) (n = 3 experiments, > 20 cells/experiment).

N MCF-7 cells expressing GFP-INPP4B or GFP-vector were transfected with NT, *PIK3CA* #1, or *PIK3CA* #2 siRNA. After 24 h, cells were lysed and immunoblotted with PI3K p110α antibodies, and GAPDH antibodies as a loading control.

O, P MCF-7 cells expressing GFP-INPP4B or GFP-vector were treated with 2 μM BYL719 (PI3Kα inhibitor) or DMSO as a vehicle control for 24 h, then fixed and immunostained with LAMP1 antibodies, and co-stained with DAPI and phalloidin (O). Data represent the number of $LAMP1^+$ puncta relative to cell area (μm^2) (n = 3 experiments, > 50 cells per experiment) (P).

Data information: Data are presented as mean ± SD. The insets at the bottom of each image are higher power regions of the boxed areas. Scale bar is 10 μm in (A, C, E, G, I, O). *P* values determined by one-way ANOVA with Tukey *post hoc* test in (B, F, P), by two-tailed unpaired *t* test in (D), or by one-way ANOVA in (H).
Source data are available online for this figure.

© 2022 The Authors

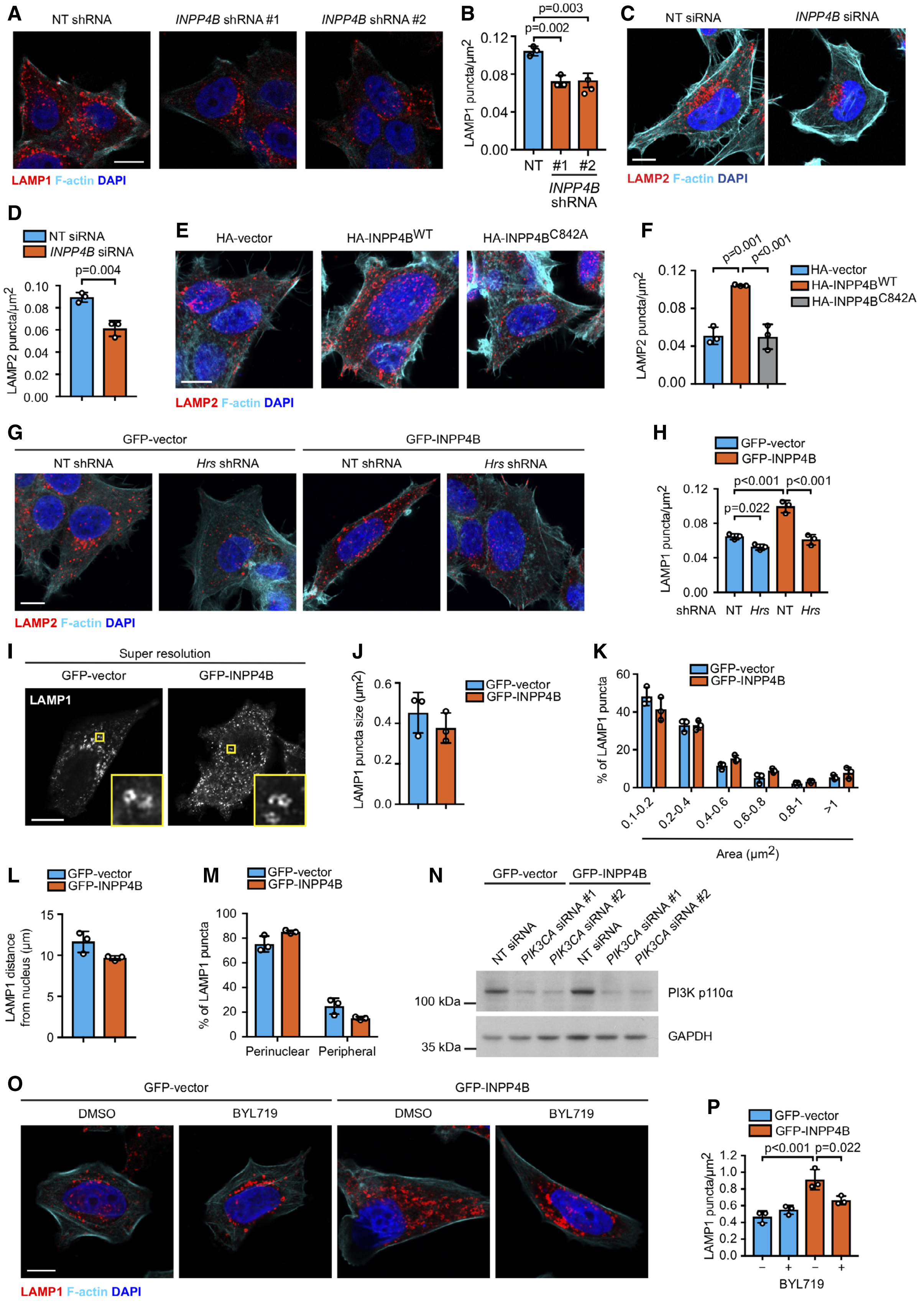

Figure EV2.

© 2022 The Authors

Figure EV3. INPP4B does not regulate mTOR-dependent lysosome biogenesis.

A–C MCF-7 cells expressing GFP-INPP4B or GFP-vector were cultured in growth media, EBSS (4 h), or EBSS (4 h) followed by 10% FCS (30 min). Cells were lysed and immunblotted with $pAKT^{S473}$, AKT(pan), $pS6K^{T389,}$ and S6K antibodies, and GAPDH antibodies as a loading control (A). Data represent the relative $pAKT^{S473}$ levels normalized to AKT(pan) (B) or $pS6K^{T389}$ levels normalized to S6K (C), and expressed relative to growth media-treated GFP-vector cells which were assigned an arbitrary value of 1 ($n = 3$ experiments).

D, E MCF-7 cells expressing GFP-INPP4B or GFP-vector were cultured in growth media, EBSS (4 h) or EBSS (4 h) followed by 10% FCS (30 min). Cells were fixed and immunostained with $pmTOR^{S2448}$ and LAMP1 antibodies, and co-stained with DAPI (D). Data represent mean $pmTOR^{S2448}$ fluorescence intensity overlapping with $LAMP1^{+}$ puncta expressed relative to growth media-treated GFP-vector cells which were assigned an arbitrary value of 1 ($n = 3$ experiments) (E).

F MCF-7 cells expressing GFP-INPP4B or GFP-vector were cultured in growth media or EBSS for 4 h. RNA was extracted and two-step quantitative RT-PCR was performed using primers for *LAMP1*, *ATP6V1C1*, *ATP6V0D1*, *CTNS*, *TPP1*, or *M6PR*, and expression was normalized to *ACTB*. Expression was determined using the $\Delta\Delta$Ct method and expressed relative to growth media-treated GFP-vector cells, which were assigned an arbitrary value of 1 ($n = 3$ experiments).

Data information: Data are presented as mean $\pm$ SD. The insets at the bottom of each image are higher power regions of the boxed areas. Scale bar is 10 μm in (D). *P* values determined by two-way ANOVA in (B, C, F).
Source data are available online for this figure.

© 2022 The Authors

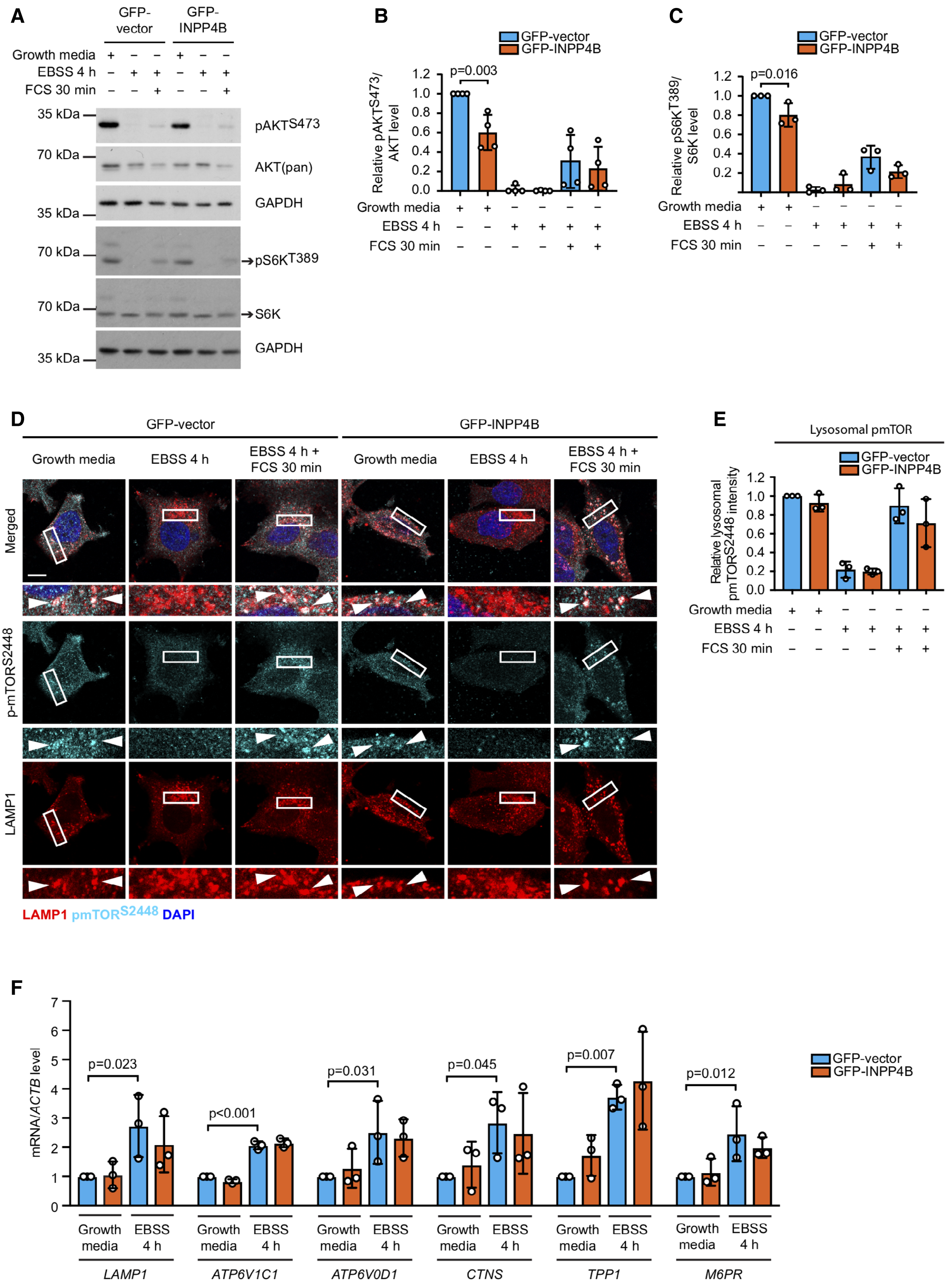

Figure EV3.

© 2022 The Authors

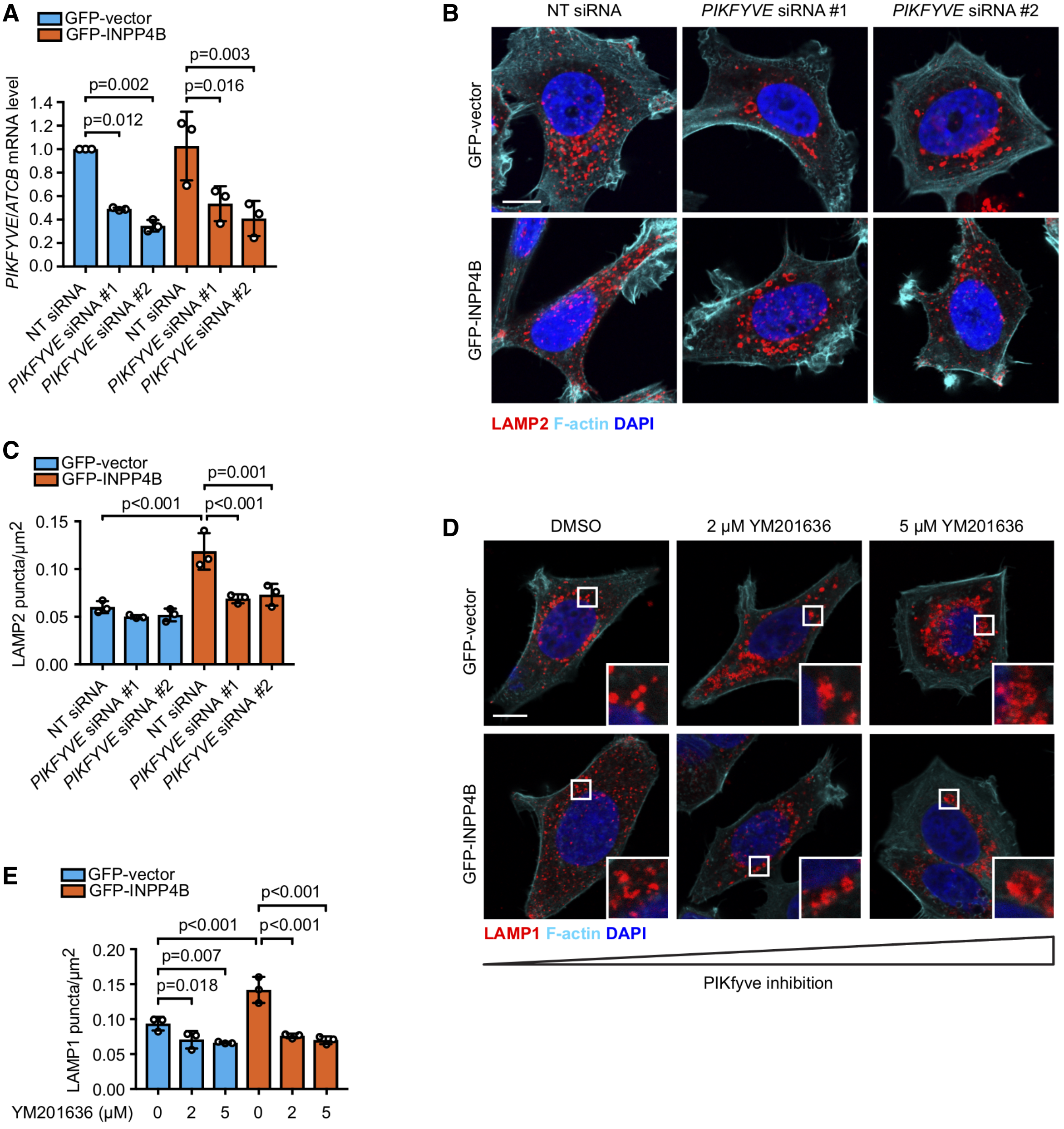

Figure EV4. INPP4B promotes PIKfyve-dependent lysosome reformation.

A MCF-7 cells expressing GFP-INPP4B or GFP-vector were transfected with nontargeted (NT), *PIKFYVE* #1, or *PIKFYVE* #2 siRNA. After 24 h, RNA was extracted and two-step quantitative RT-PCR was performed using primers for *PIKFYVE*, and expression was normalized to *ACTB*. Expression was determined using the ΔΔCt method and expressed relative to GFP-vector;NT siRNA cells which were assigned an arbitrary value of 1 (n = 3 experiments).

B, C MCF-7 cells expressing GFP-INPP4B or GFP-vector were transfected with NT, *PIKFYVE* #1, or *PIKFYVE* #2 siRNA. After 24 h, cells were fixed and immunostained with LAMP2 antibodies, and co-stained with DAPI and phalloidin (B). Data represent the number of LAMP2$^+$ puncta relative to cell area (μm^2) (n = 3 experiments, > 40 cells per experiment) (C).

D, E MCF-7 cells expressing GFP-INPP4B or GFP-vector were treated with 2 or 5 μM YM201636 (PIKfyve inhibitor) or DMSO as a vehicle control for 4 h. Cells were fixed and immunostained with LAMP1 antibodies, and co-stained with DAPI and phalloidin (D). Data represent the number of LAMP1$^+$ puncta relative to cell area (μm^2) (n = 3 experiments, > 30 cells/experiment) (E).

Data information: Data are presented as mean ± SD. The insets at the lower right of each image are higher power regions of the boxed areas. Scale bar is 10 μm in (B, D). *P* values determined by one-way ANOVA with Tukey *post hoc* test in (A, C), or by one-way ANOVA in (E).
Source data are available online for this figure.

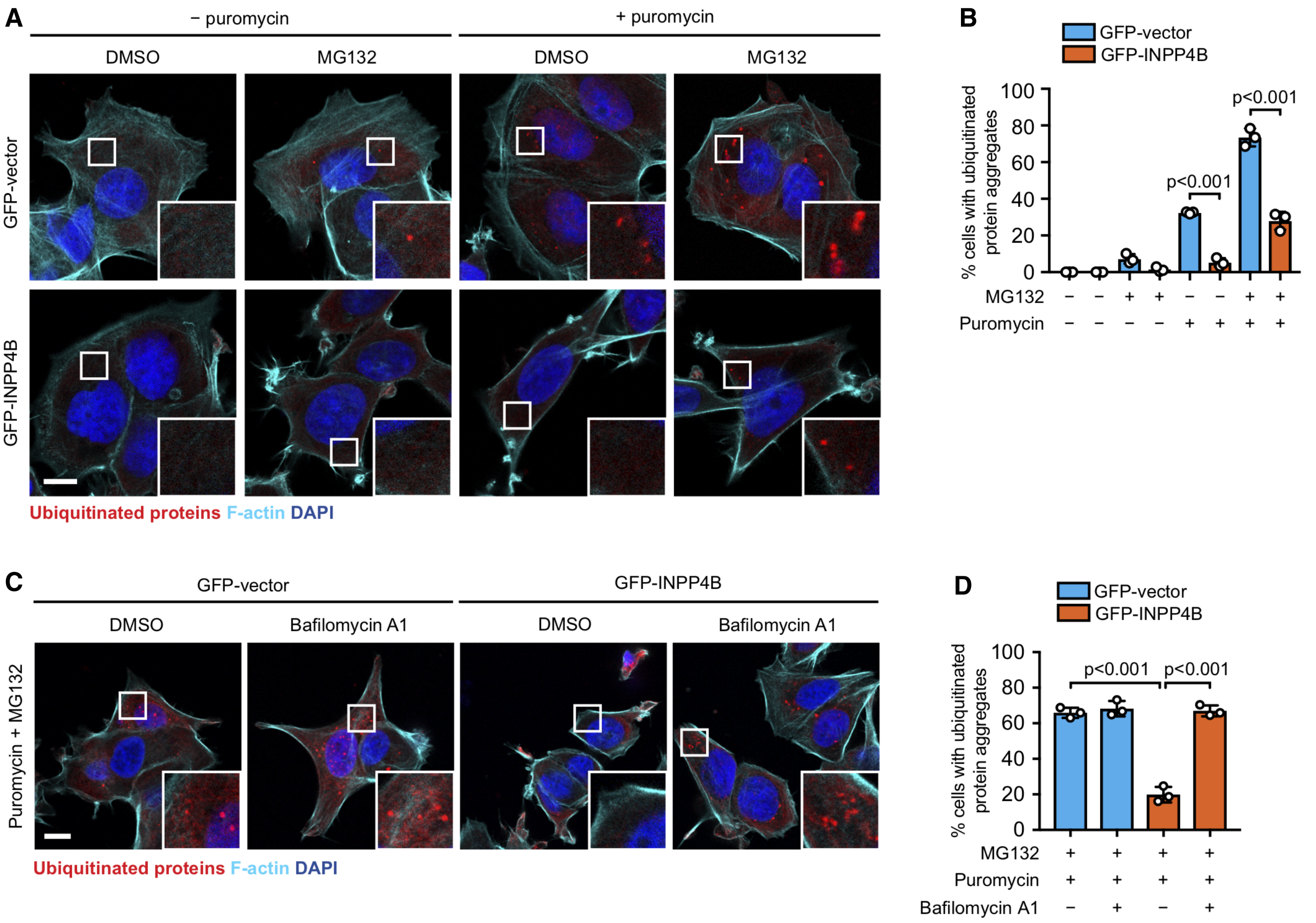

Figure EV5. INPP4B promotes autophagic degradation of protein aggregates during proteotoxic stress.

A, B MCF-7 cells expressing GFP-INPP4B or GFP-vector were treated for 4 h with 10 μg/ml puromycin ±5 μm MG132 or DMSO as a vehicle control. Cells were fixed and immunostained with ubiquitin antibodies, and co-stained with DAPI and phalloidin (A). Data represent the percentage of cells with ubiquitinated protein aggregates (n = 3 experiments, > 200 cells/experiment) (B).

C, D MCF-7 cells expressing GFP-INPP4B or GFP-vector were treated for 4 h with 10 μg/ml of puromycin, 5 μm of MG132, and either 100 nM of bafilomycin A1 or DMSO as a vehicle control. Cells were fixed and immunostained with ubiquitin antibodies, and co-stained with DAPI and phalloidin (C). Data represent the percentage of cells with ubiquitinated protein aggregates (n = 3 experiments, > 200 cells/experiment) (D).

Data information: Data are presented as mean ± SD. The insets at the lower right or bottom of each image are higher power regions of the boxed areas. Scale bar is 10 μm in (A, C). *P* values determined by one-way ANOVA in with Tukey *post hoc* test in (B, D).
Source data are available online for this figure.

 © 2022 The Authors
