# Supplementary figures and images for: Endosome maturation links PI3Kα signaling to lysosome repopulation during basal autophagy
Source: EMBO J. 2022 Aug 15;41(19):e110398. doi: 10.15252/embj.2021110398 (PMC9531306; doi:10.15252/embj.2021110398)

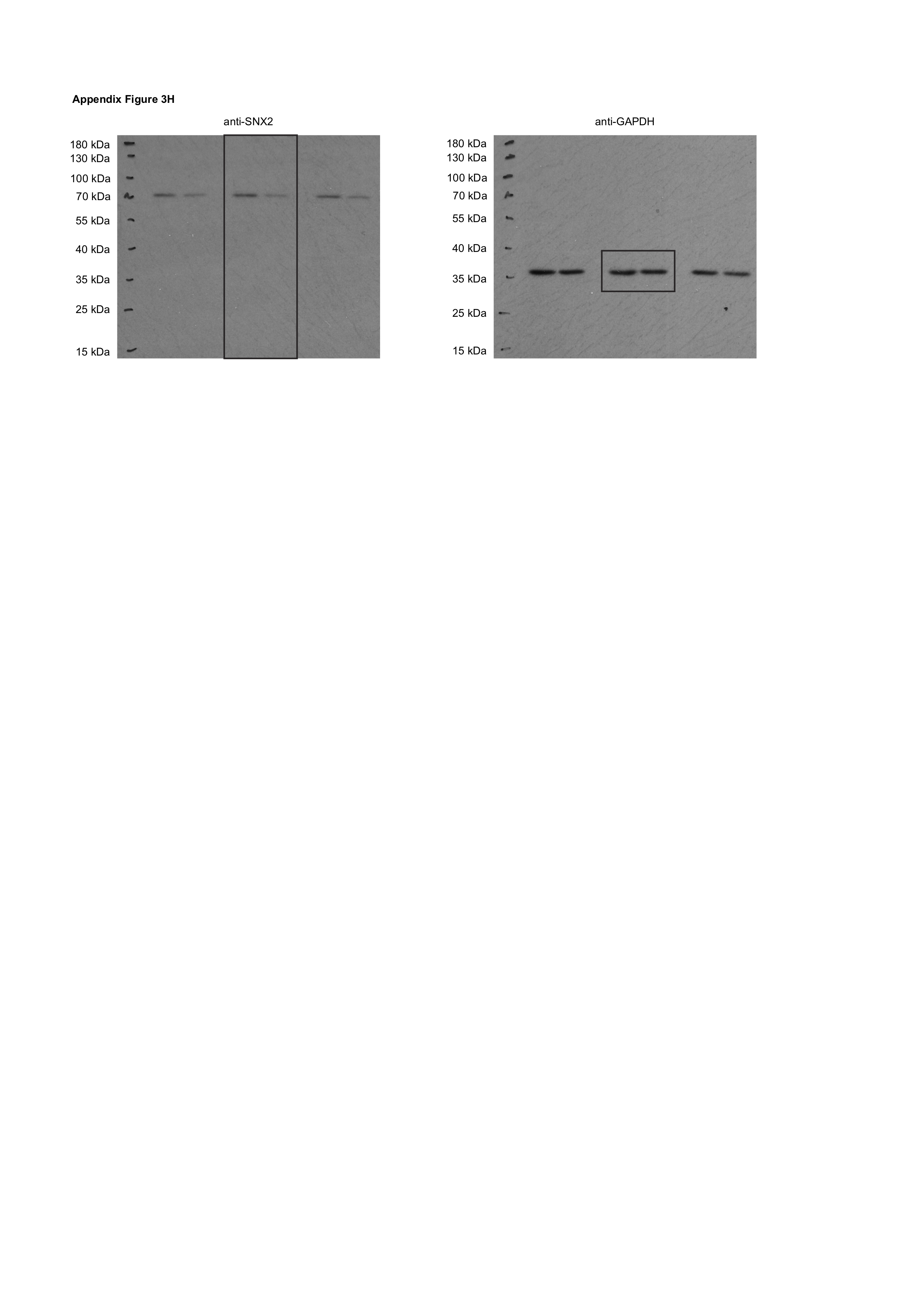

Supplement: Supplementary file 7 — Source Data for Expanded View and Appendix [file EMBJ-41-e110398-s015.zip › EMBOJ-2021-110398_SourceDataForExpandedViewAndAppendix/Appendix Fig 3/Uncropped blots Appendix Fig 3.tiff]

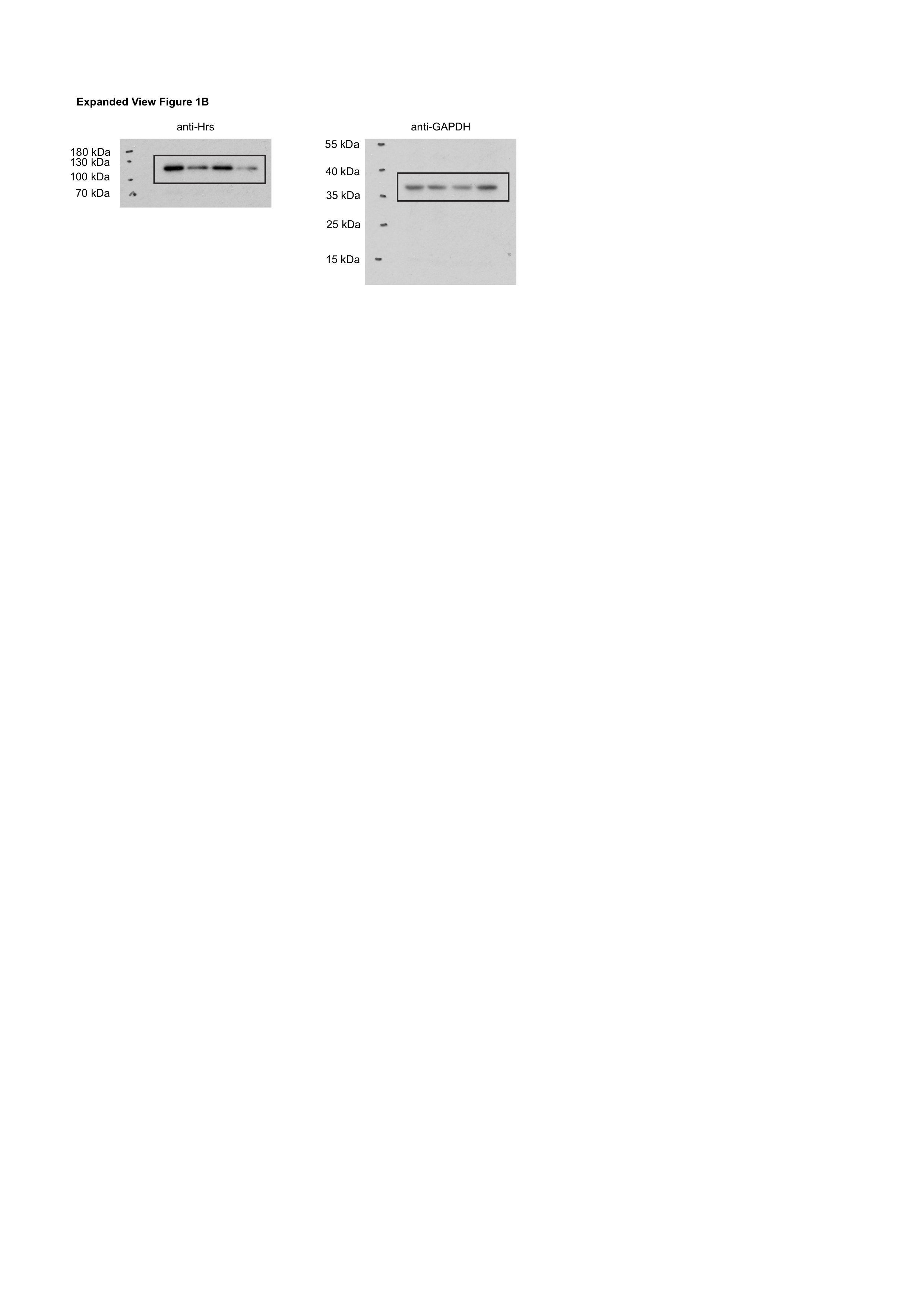

Supplement: Supplementary file 7 — Source Data for Expanded View and Appendix [file EMBJ-41-e110398-s015.zip › EMBOJ-2021-110398_SourceDataForExpandedViewAndAppendix/Fig EV1/Uncropped blots Fig EV1.tiff]

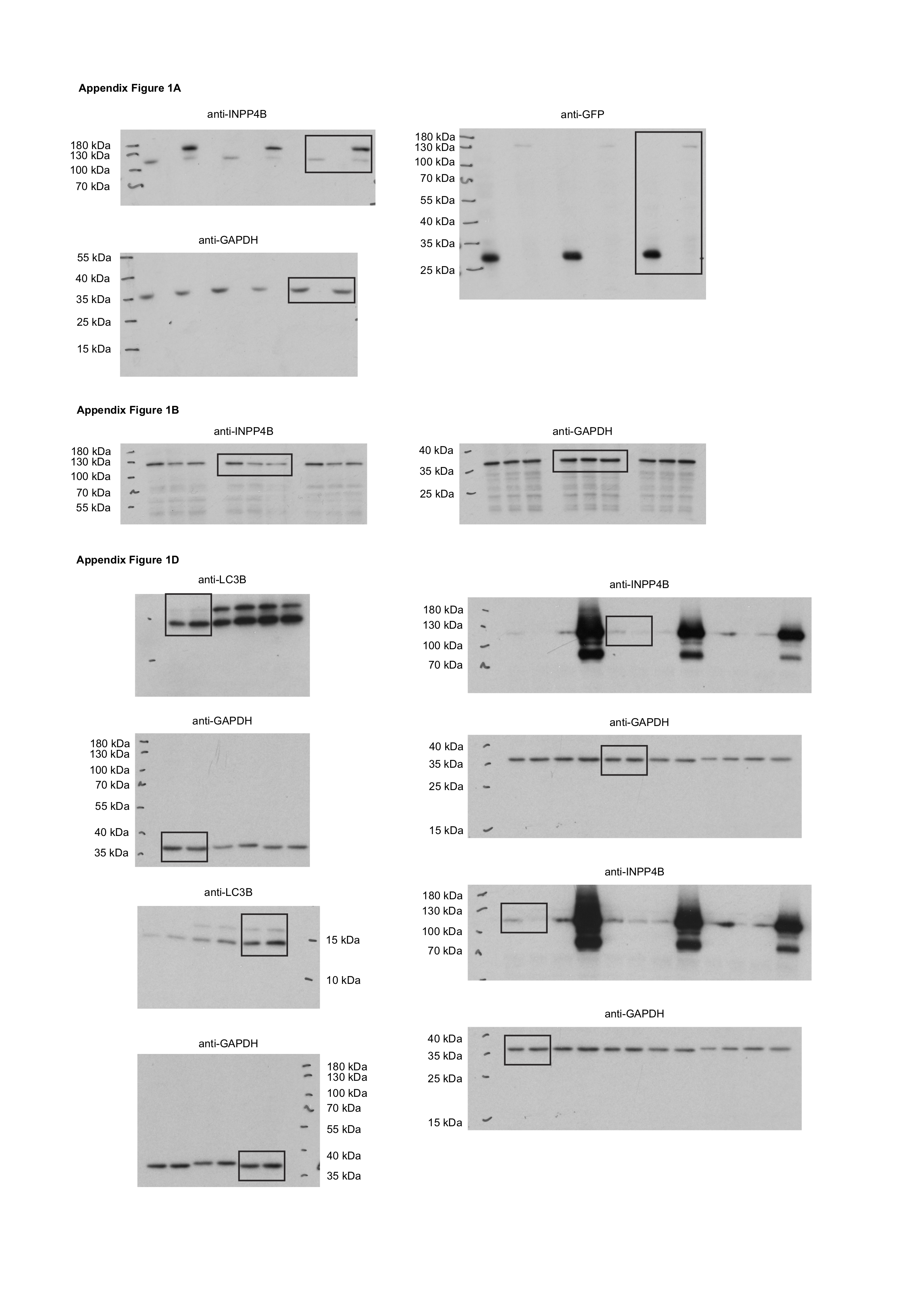

Supplement: Supplementary file 7 — Source Data for Expanded View and Appendix [file EMBJ-41-e110398-s015.zip › EMBOJ-2021-110398_SourceDataForExpandedViewAndAppendix/Appendix Fig 1/Uncropped blots Appendix Fig 1.tiff]

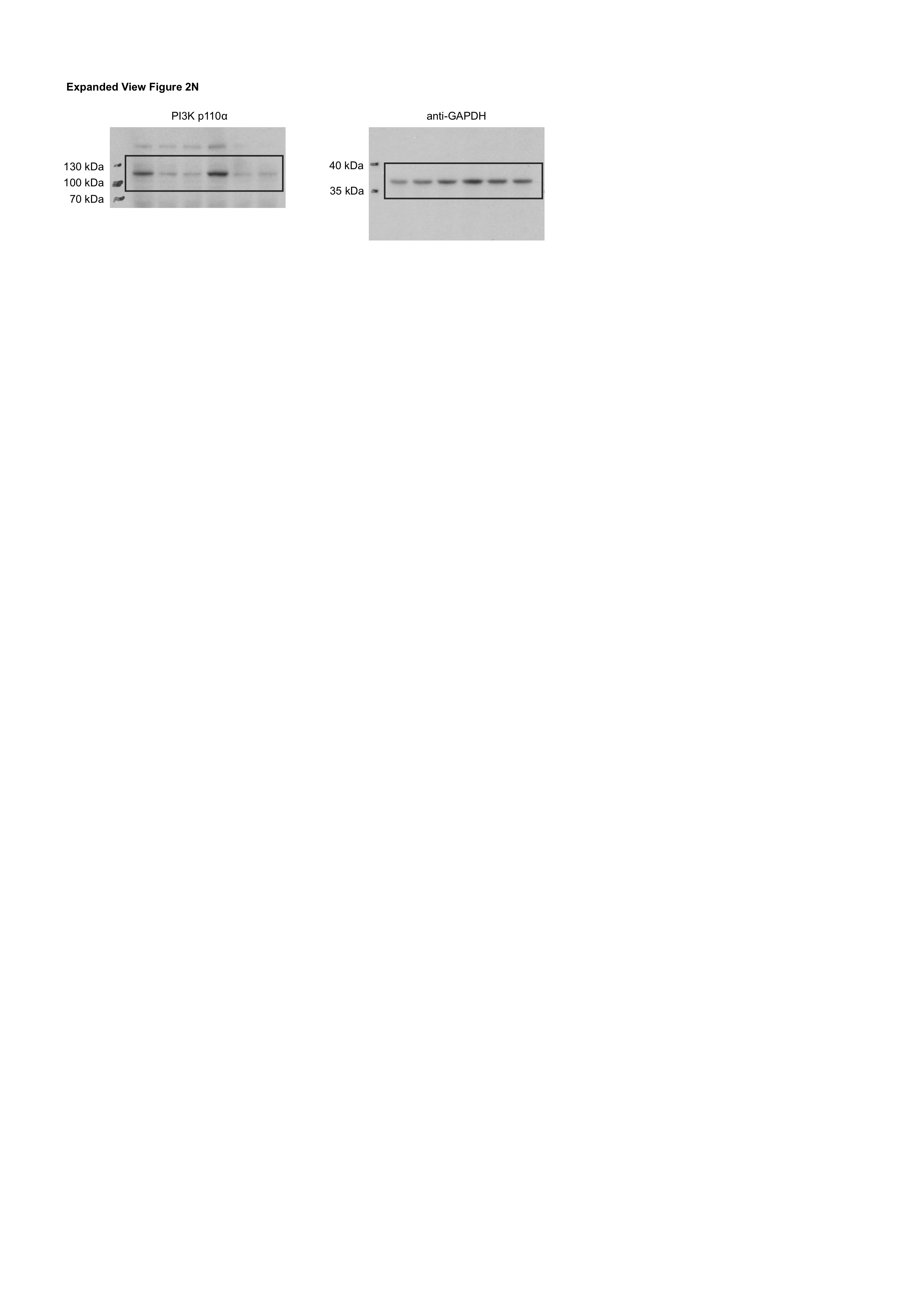

Supplement: Supplementary file 7 — Source Data for Expanded View and Appendix [file EMBJ-41-e110398-s015.zip › EMBOJ-2021-110398_SourceDataForExpandedViewAndAppendix/Fig EV2/Uncropped blots Fig EV2.tiff]

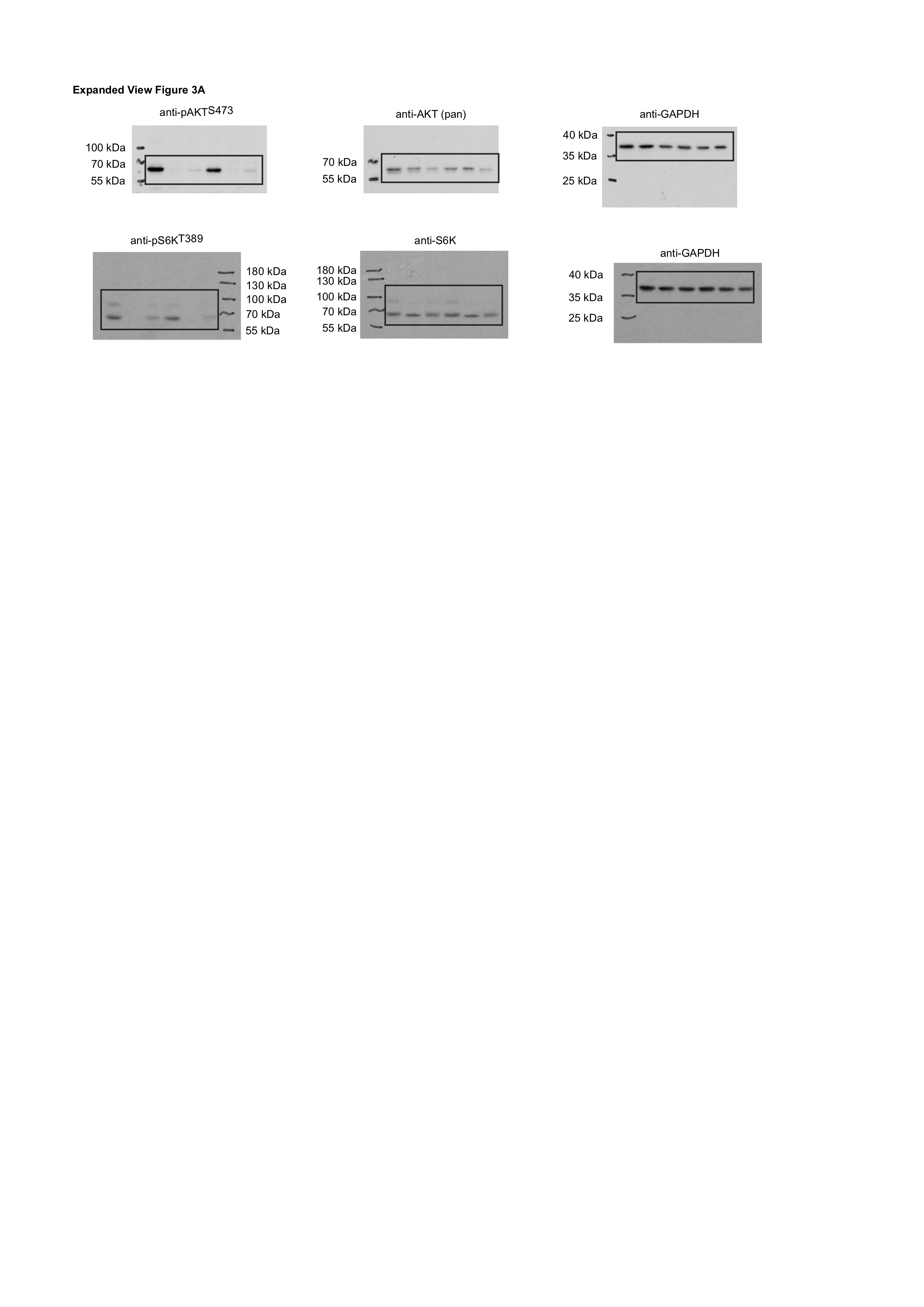

Supplement: Supplementary file 7 — Source Data for Expanded View and Appendix [file EMBJ-41-e110398-s015.zip › EMBOJ-2021-110398_SourceDataForExpandedViewAndAppendix/Fig EV3/Uncropped blots Fig EV3.tiff]

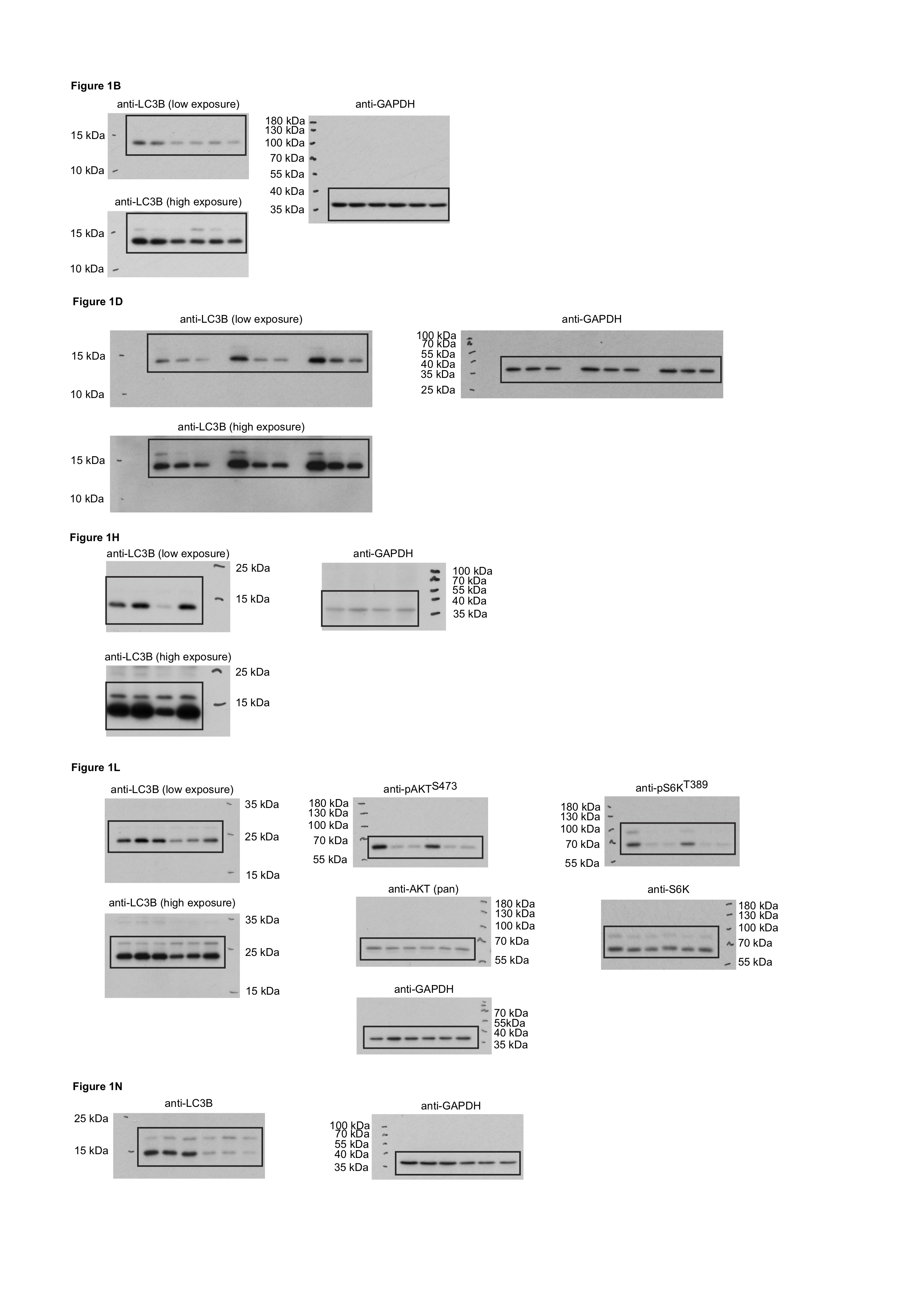

Supplement: Supplementary file 8 — Source Data for Figure 1 [file EMBJ-41-e110398-s004.zip › EMBOJ-2021-110398_SourceDataForFigure1/Uncropped blots Fig 1.tiff]

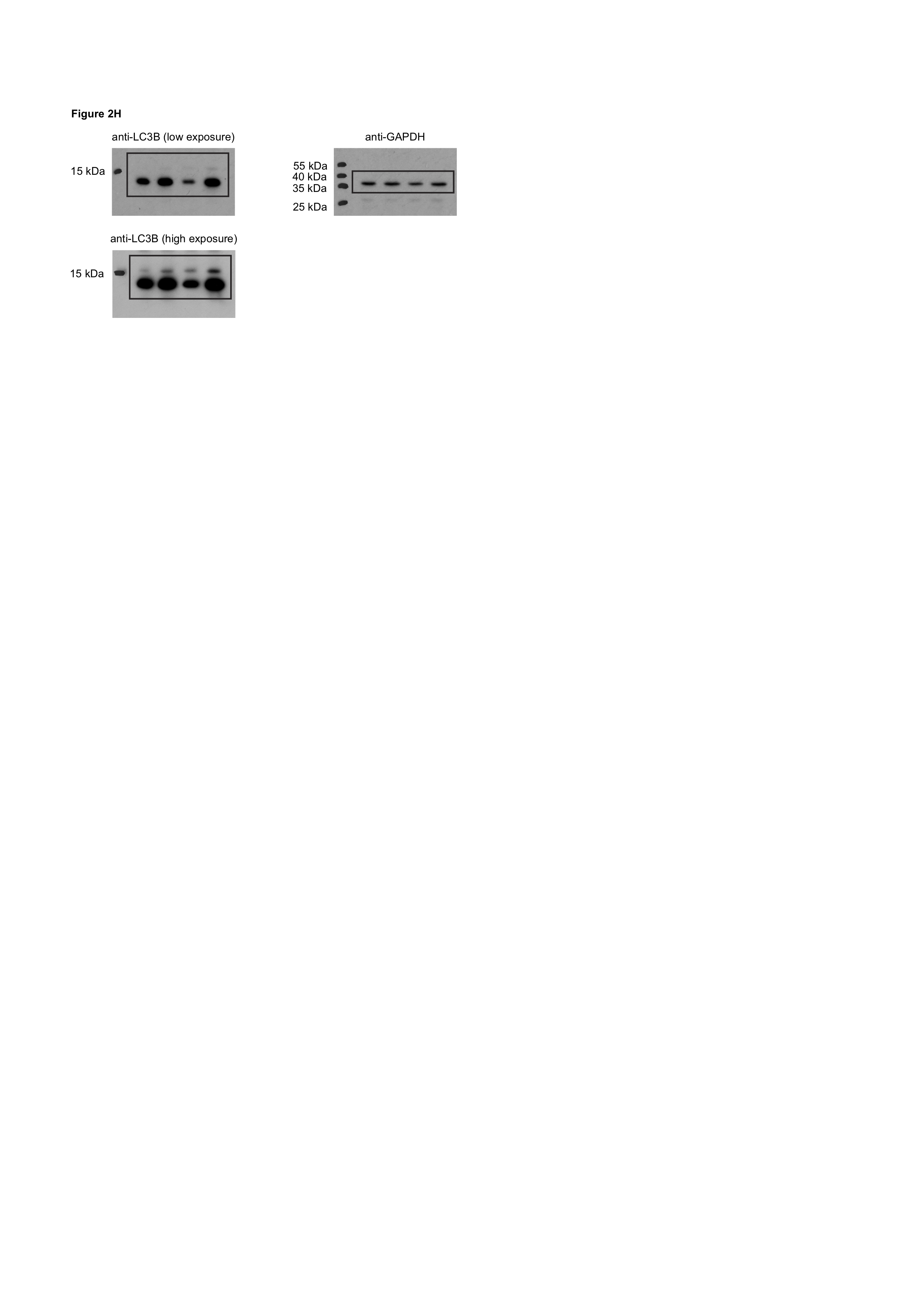

Supplement: Supplementary file 9 — Source Data for Figure 2 [file EMBJ-41-e110398-s016.zip › EMBOJ-2021-110398_SourceDataForFigure2/Uncropped blots Fig 2.tiff]

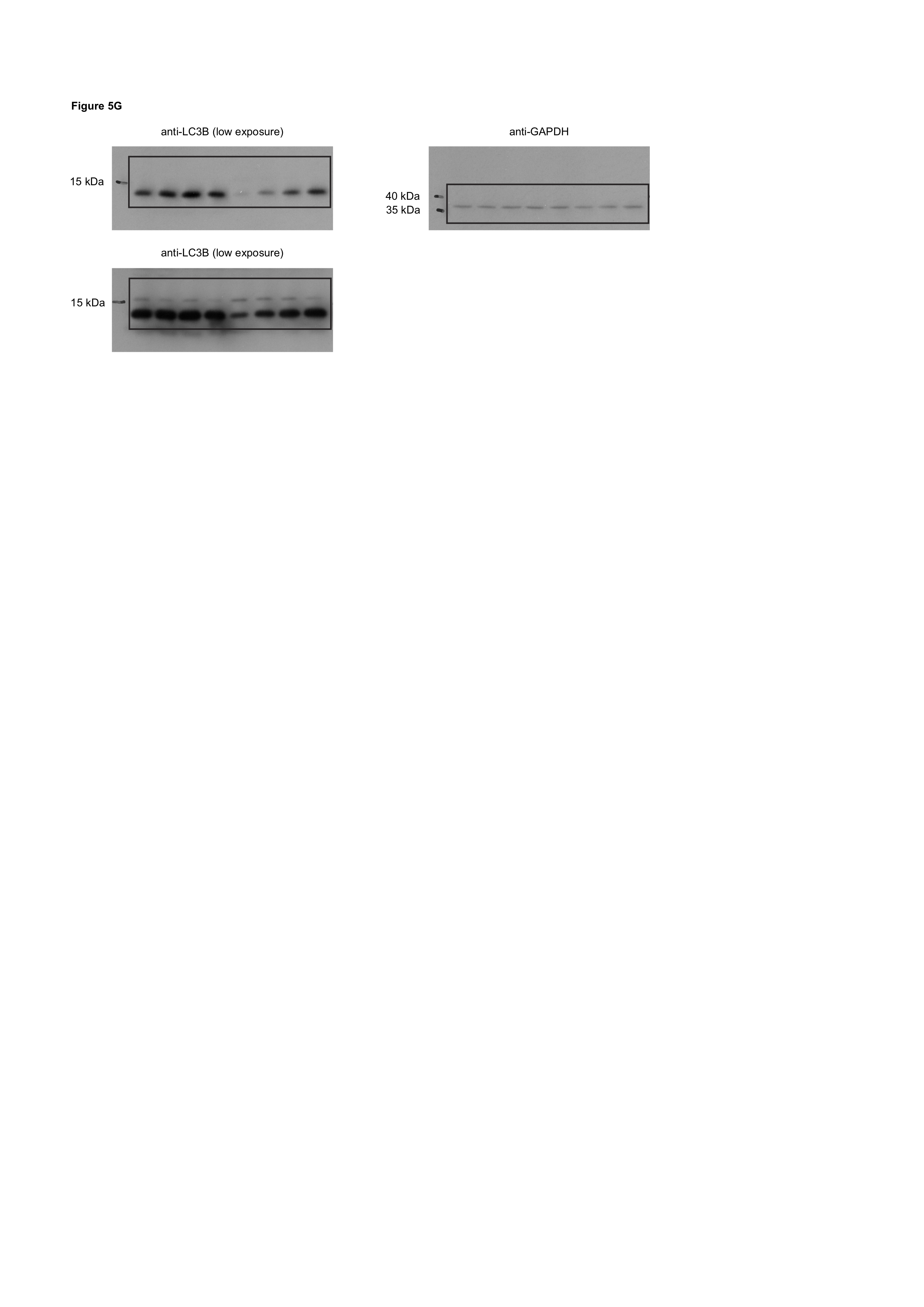

Supplement: Supplementary file 12 — Source Data for Figure 5 [file EMBJ-41-e110398-s005.zip › EMBOJ-2021-110398_SourceDataForFigure5/Uncropped blots Fig 5.tiff]
