# Supplementary material for: Endosome maturation links PI3Kα signaling to lysosome repopulation during basal autophagy
Source: EMBO J. 2022 Aug 15;41(19):e110398. doi: 10.15252/embj.2021110398 (PMC9531306; doi:10.15252/embj.2021110398)
Supplement: Supplementary file 16 [file EMBJ-41-e110398-s002.pdf]

THE EMBO JOURNAL

Endosome maturation links PI3Kα signaling to lysosome repopulation during basal autophagy

Samuel Rodgers, Emily Jones, Senthil Arumugam, Sabryn Hamila, Jill Danne, Rajendra Gurung, Matthew Eramo, Randini Nanayakkara, Georg Ramm, Meagan McGrath, and Christina Mitchell
DOI: 10.15252/embj.2021110398

Corresponding author(s): Christina Mitchell (christina.mitchell@monash.edu)

Review Timeline:

Submission Date:	14th Dec 21
Editorial Decision:	5th Jan 22
Revision Received:	23rd Feb 22
Editorial Decision:	29th Mar 22
Revision Received:	1st Jun 22
Editorial Decision:	15th Jul 22
Revision Received:	19th Jul 22
Accepted:	21st Jul 22

Editor: William Teale

Transaction Report:

(Note: With the exception of the correction of typographical or spelling errors that could be a source of ambiguity, letters and reports are not edited. Depending on transfer agreements, referee reports obtained elsewhere may or may not be included in this compilation. Referee reports are anonymous unless the Referee chooses to sign their reports.)

1st Editorial Decision 5th Jan 2022

Dear Prof. Mitchell,

Thank you for submitting your manuscript for consideration by the EMBO Journal.

Your manuscript was sent to two referees and we have now received the comments from both of them. As you will see from the reports which are included below, neither referee is able to offer the publication of your manuscript in the EMBO Journal their full support.

Given these opinions and the fact that the EMBO Journal can only afford to accept papers which receive enthusiastic support from both referees, we see little choice other than to decide not to offer publication at EMBO Journal.

While we cannot pursue this manuscript further, we encourage you to transfer your study to our not-for-profit open-access sister journal, Life Science Alliance (LSA). We shared your manuscript and the accompanying reviews with LSA Executive Editor, Eric Sawey, who is interested in these findings, and would like to invite further consideration of this manuscript at LSA pending the following revisions:

- Address Reviewer 1's comments
- Address Reviewer 2's Main Points, only if data is readily available, plus all Minor Points

We understand that such a revision might need to be re-reviewed, in which case, Dr. Sawey will walk the Reviewers through our transfer process.

We encourage you to use the link below to transfer your manuscript to LSA. You do not need to revise the manuscript before transferring it to LSA. Once you transfer, Dr. Sawey will email you an invitation to revise and resubmit, listing the same revision requests as mentioned above. Please feel free to reach out at e.sawey@life-science-alliance.org if you have any questions about the LSA journal, the transfer process or the revisions requested.

Yours sincerely,

William Teale

William Teale, PhD
Editor
The EMBO Journal
w.teale@embojournal.org

Referee #1:

The manuscript by Rodgers and co-workers builds on recent findings from the authors that the PI 4-phosphatase IMPP4B plays an important role in regulation of endosomal functions. The authors here report that the class I PI 3-kinase PIK3CA (PI3Ka) mediates basal (but not starvation-induced) autophagy via PI(3,4,5)P3, which is subsequently converted to PI(3,4)P2 by a 5-phosphatase at the plasma membrane and then to PI(3)P via INPP4B on late endosomes. This PI(3)P pool was found to act as substrate for the PI(3)P 5-kinase PIKfyve to produce PI(3,5)P2, and SNX2 was suggested to be recruited by this phosphoinositide to mediate endolysosome reformation.
The proposed concept is an interesting one, but parts of the underpinning data are not convincing.

Major points:

1. The authors interchangeably refer to "lysosome reformation" and "endolysosome reformation". It is not entirely clear what they mean, and they need to revise the text for consistency, but presumably they refer to the reformation of lysosomes from hybrid organelles that originate from fusion of lysosomes with late endosomes. However, the assay they use for measuring "lysosome reformation" is spinning-disk microscopy of cells expressing LAMP1-mCherry and monitoring scission of LAMP1 structures. If the authors want to imply that the fragments being abscised from LAMP1 vesicles represent reformed lysosomes (as opposed to, for instance, recycling tubules), they need to provide evidence for this.

2. The idea that basal autophagy is mediated by PIK3CA is interesting, but under physiological conditions PIK3CA activity is likely to fluctuate. This raises the question whether increased PIK3CA activity would increase autophagy, as suggested by the model in Figure 7. The authors should investigate this.

3. The most speculative part of the manuscript is the last section that deals with PIKfyve and SNX2. As the authors mention, SNX2 binds to both PI(3)P and PI(3,4)P2 in addition to PI(3,5)P2, and binding to PI(3)P seems to have highest affinity. It is therefore not clear how SNX2 would be specifically recruited to endolysosomes via PI(3,5)P binding, especially because SNX2 has been reported to localize to early endosomes (for instance, Mellado et al., Biol.Cell, 2014). This proposal would have been strengthened if the authors could show colocalization between SNX2 and PI(3,5)P2 on endolysosomes. The effect of PIKfyve inhibition on SNX2 localization (Fig 6F) is not convincing since the strong effect of YM210636 on endolysosome morphology makes comparisons difficult. Immunoelectron microscopy could resolve this issue.

4. In general, the authors use RNAi technology to deplete proteins of interest, and, as the authors are well aware of, this technology frequently comes with the disadvantage of off-target effects. Key effects of RNAi-mediated knock-downs must be verified by rescue transfections with RNAi-resistant plasmids.

Minor points:

1. The authors should use standard nomenclature for PIK3CA.

2. The authors frequently omit "that" from sentences, making them difficult to comprehend. For instance, "..here we show SNX2 is required..". The text should be revised for clarity.

3. In Figure 7, lysosomes are formed from an "endolysosome" via "endolysosome reformation". This should be "lysosome reformation"?

Referee #2:

In this manuscript, Rodgers et al focus on mechanisms controlling lysosome "repopulation" under nutrient-rich conditions and their impact on basal autophagy. They identify a phosphoinositide pathway operating on the endolysosomal system where type I PI 3-Kinase alpha controls lysosome repopulation by generating a pool of PI(3,4,5)P3 that is converted to PI(3,4)P2 and PI(3)P, prior to phosphorylation to PI(3,5)P2 by lipid kinase PIKfyve. Specifically, the authors show, using a combination of silencing and overexpression approaches, that PI3Kalpha-derived PI(3)P is generated by PI phosphatase INPP4B on late endosomes and maintained on these organelles as they mature into lysosomes. A key effector of PI(3,5)P2 in this context is determined to be SNX2, a SNX-BAR protein involved in lysosomal tubulation/fission. Importantly, they show that this pathway is only relevant under nutrient-rich conditions and thus for basal autophagy, whereas nutrient deprivation-induced autophagy relies more on the better understood Vps34 pathway.

This is an interesting manuscript that provides additional evidence for the existence of Vps34-independent sources of PI(3)P and their physiological relevance in cells, following up on a number of studies from the same group. Mechanisms controlling basal autophagy are poorly understood and this study offers a novel perspective, through a careful mechanistic dissection of the phosphoinositide pathway and their associated signaling components. While the study is generally well conducted and interpreted, it does not address in depth the physiological or pathophysiological consequence of reduced basal autophagy, as achieved by manipulation of the INPP4B pathway. Additionally, it is unclear whether findings are limited to MCF-7 cells or whether they have broader implications. This limits the impact of the study and in the absence of the following suggested revisions, it may be more appropriate for specialized cell biology journals.

Main points:

1. The authors clearly show an impact of INPP4B knockdown and overexpression on basal autophagy, based on analysis of LC3. Can the authors demonstrate that these changes in basal autophagy achieved by INPP4B modulation under nutrient-rich conditions affect cellular responses in any meaningful way? For instance, are their specific autophagy cargoes that accumulate as a result of these changes and cause cellular dysfunction? Examining p62 levels (as the authors do) is a start, but it seems that a more thorough investigation would be in order here. This could be addressed by looking at pathological proteins/autophagy cargoes under more chronic conditions in intact cells in the presence of nutrients or in vivo using KO mice.
2. Studies are essentially conducted in MCF-7 cells. They would be more impactful if the key findings were confirmed in independent cell lines.

Minor points:

3. Some studies have shown that class II PI3Ks contribute to PI3P levels, including in autophagy settings. This should be mentioned in the introduction.
4. Line 136, bafilomycin is defined as an "autophagosome-lysosome fusion inhibitor" but as the authors know, it is primarily a

proton pump inhibitor and this should be mentioned; also it should be referred to as "bafilomycin A1".
5. Lane 397, Fig4 is primarily described as a lipid phosphatase rather than a lipid/protein kinase.

** As a service to authors, EMBO Press provides authors with the possibility to transfer a manuscript that one journal cannot offer to publish to another EMBO publication or the open access journal Life Science Alliance launched in partnership between EMBO Press, Rockefeller University Press and Cold Spring Harbor Laboratory Press. The full manuscript and if applicable, reviewers' reports, are automatically sent to the receiving journal to allow for fast handling and a prompt decision on your manuscript. For more details of this service, and to transfer your manuscript please click on Link Not Available. **

Please do not share this URL as it will give anyone who clicks it access to your account.

Dear Prof. Facundo Batista and Dr. William Teale,

We are writing to appeal the recent rejection of our manuscript EMBOJ-2021-110398, entitled "*Endosome maturation links PI3Kα signaling to lysosome repopulation during basal autophagy*" by Samuel Rodgers *et al.* We apologize for the delay in submitting this appeal due to the Christmas shutdown period and COVID-19 absences.

The basis of our appeal is the following:
It is difficult for us to understand why the manuscript was rejected based on the reviewers' concerns provided via the transparent peer review system. The reviewers described the manuscript positively and as being *"interesting"*. Reviewer 2 highlighted the "*novel perspective"* on basal autophagy that comes from our *"careful mechanistic dissection of the phosphoinositide pathway"* and that the study is *"well conducted and interpreted"*. Therefore, we request an opportunity to respond to their concerns and submit a revised manuscript, which we can undertake in a single round of revision.

Reviewer concerns:

- ***Reviewer 1*** indicates only that "*parts of our underpinning data are not convincing"*. It should be noted several "*major points*" raised by the reviewer are already answered by existing literature, or shown by our data in the current manuscript. In addition, we have the capacity to undertake additional experiments that directly address the reviewer's specific concerns.
- ***Reviewer 2*** indicates that we should explore the "*physiological/pathophysiological consequences of reduced basal autophagy*". We have demonstrated in unpublished work that INPP4B enhances autophagic degradation of protein aggregates in response to proteotoxic stress, which addresses the reviewer's concern.

If the manuscript was rejected based on a lack of significance or "*enthusiastic support*", we would argue against this with the following observations:

- The autophagy field has focused on lysosome repopulation pathways that only operate during starvation-induced autophagy including TFEB-dependent *de novo* lysosome biogenesis and autophagic lysosome reformation. Our study addresses a significant knowledge gap by describing how lysosome homeostasis is regulated under basal conditions to sustain autophagy.
- Interrogation of lysosome reformation pathways is of substantial current interest, as shown by recent studies:
    - Wang et al. *Nat Commun*. 2021. PMID: 34417467.
    - Wu et al. *Autophagy*. 2021. PMID 33629936.
    - Khundadze et al. *Autophagy*. 2021. PMID: 33618608.
    - Hirst et al. *J Cell Biol*. 2021. PMID: 33464297.
    - McGrath et al. *J Clin Invest.* 2021. PMID: 33119550.
- Our study aligns with the renewed interest in the functional interplay between the endosome and autophagy pathways:
    - Kumar et al. *Cell*. 2021. PMID: 34741801.
    - Wu et al. *Nat Commun.* 2021. PMID: 34789781.

- Wilfling et al. *Mol Cell*. 2020. PMID: 33207182

Below we provide a response to the reviewer’s comments and outline experimental plans to demonstrate how we can directly address all of the concerns raised by the reviewers in a timely manner.

We hope that you will reconsider our manuscript since we can comprehensively address the reviewer concerns. We welcome any further contact and discussion with you regarding this matter and request that you reconsider a revised manuscript.

Yours sincerely,

Christina Mitchell

REVIEWER COMMENTS

Reviewer #1

Major points:

1. The authors interchangeably refer to "lysosome reformation" and "endolysosome reformation". It is not entirely clear what they mean, and they need to revise the text for consistency, but presumably they refer to the reformation of lysosomes from hybrid organelles that originate from fusion of lysosomes with late endosomes.

The reviewer is correct, our study examines the reformation of lysosomes that are derived from endolysosome membranes (i.e. a hybrid organelle originating from fusion of a late endosome with a lysosome). The name of this process has not been properly defined in the literature, and has been referred to by different names such as:

- Lysosome reformation from endolysosomes
  - Bissig et al. *Traffic*. 2017. PMID: 28857423
  - Wilden et al. *Biochim Biophys Acta Mol Cell Res*. 2019. PMID: 2996662
- Endocytic lysosome reformation
  - Gan et al. *J Cell Biol*. 2019. PMID: 31235480
  - Yang and Wang. *J Cell Biol*. 2021. PMID: 33950241
- Lysosome reformation
  - Miller et al. *Traffic*. 2015. PMID: 25491304
  - Bright et al. *Curr Biol*. 2005. PMID: 15723798
  - Bright et al. *Curr Biol*. 2016. PMID: 27498570

We agree that our use of the term "*endolysosome reformation*" is slightly confusing. To improve clarity in the manuscript we will refer to this process as "*lysosome reformation from endolysosomes*" in line with the selected studies above.

The assay they use for measuring "lysosome reformation" is spinning-disk microscopy of cells expressing LAMP1-mCherry and monitoring scission of LAMP1 structures. If the authors want to imply that the fragments being abscised from LAMP1 vesicles represent reformed lysosomes (as opposed to, for instance, recycling tubules), they need to provide evidence for this.

The referee's suggestion that the LAMP1-positive structures observed by spinning disk microscopy may represent "*recycling tubules*" is not consistent with the field, which describes recycling tubules as those originating from early and recycling endosomes that do not contain LAMP1. For example, LAMP1 does not co-localize with the early endosome protein EEA1 (Kamentseva et al. PLoS One. 2020. PMID: 32357161) or the recycling endosome proteins Transferrin Receptor (Rush and Ceresa. *Mol Cell Endocrinol*. 2013. PMID: 23933150) or KIF13A (Delevoye et al. *Cell Rep*. 2014. PMID: 24462287). To our knowledge, the only LAMP1-positive structures described to undergo scission under growth conditions are lysosomes undergoing reformation.

Our use of spinning disk microscopy of cells expressing LAMP1-mCherry is a validated approach for examining lysosome reformation from endolysosomes (Bissig et al. *Traffic*. 2017. PMID: 28857423). Furthermore, our work did not rely solely on the results from spinning disk microscopy analysis of LAMP1-mCherry. Multiple other lines of our evidence examined lysosome reformation and homeostasis and downstream functional effects on autophagy. Briefly, this included:

- Immunostaining for the lysosome-membrane associated proteins 1 and 2 (LAMP1 and LAMP2) to examine effects on lysosome homeostasis (**Fig. 3A, 3B, 3E-H, 5D-F, 6A, 6B, Supplementary Fig. 3A-I, 6B-E)**
- Imaging of MAGIC RED® Cathepsin B to examine effects on functional lysosomes (**Fig. 3C and 3D)**
- Changes to autophagic flux consistent with lysosome reformation defect, assessed by p62 immunostaining, LC3B immunoblotting, LC3 fluorescent biosensor (**Fig. 1B-M, 2H, 2I, 5G, 5H, Supplementary Fig. 1D-J).**
- Exclusion of other lysosome repopulation pathways (*de novo* lysosome biogenesis, ALR) **(Supplementary Fig. 4A-F)**

These approaches are also validated and in line with standard methodology to identify deficits in lysosome reformation as outlined in:

- Yu et al. *Nature.* 2010. PMID: 20526321.
- Rong et al. *Nat Cell Biol*. 2012. PMID: 22885770.
- Munson et al. *EMBO J.* 2015. PMID: 26139536
- Rong et al. *Proc Natl Acad Sci USA 2011.* PMID: 21518918
- Bissig et al. *Traffic*. 2017. PMID: 28857423.

Some reported studies also specifically quantify the number of terminal storage lysosomes, the initial structures formed via lysosome reformation from endolysosomes. Terminal storage lysosomes can be distinguished from endolysosomes by co-labeling lysosomes with Magic Red probes, which fluoresce in endolysosomes but not in terminal storage lysosomes (Bissig et al. *Traffic*. 2017. PMID: 28857423, Bright et al. *Curr Biol*. 2016. PMID: 27498570). We can perform these co-labeling experiments to confirm that INPP4B also regulates the number of terminal storage lysosomes.

2. The idea that basal autophagy is mediated by PIK3CA is interesting, but under physiological conditions PIK3CA activity is likely to fluctuate. This raises the question whether increased PIK3CA activity would increase autophagy, as suggested by the model in Figure 7. The authors should investigate this.

We agree with the reviewer that whether PIK3CA activity directly regulates basal autophagy is an interesting idea to explore, and this is something we can address experimentally. Our data shows that the pool of PI(3)P generated by INPP4B downstream of PIK3CA is required for lysosome reformation **(Fig. 3G and 3H)**, which is in turn required for basal autophagy. We can examine the effects of PIK3CA activity on basal autophagy by inactivating PIK3CA in MCF-7 cells which harbor a hyperactivating $PIK3CA^{E545K}$ mutation, and/or by expressing hyperactivate $PIK3CA^{H1047R}$ mutant protein in cells with wild-type PIK3CA (eg HeLa, HEK293).

3. The most speculative part of the manuscript is the last section that deals with PIKfyve and SNX2. As the authors mention, SNX2 binds to both PI(3)P and PI(3,4)P2 in addition to PI(3,5)P2, and binding to PI(3)P seems to have highest affinity. It is therefore not clear how SNX2 would be specifically recruited to endolysosomes via PI(3,5)P2 binding, especially because SNX2 has been reported to localize to early endosomes (for instance, Mellado et al., Biol.Cell, 2014).

Despite the fact that there is an established role for SNX2 at endosomes via PI(3)P-binding, this does not preclude a separate functional role for SNX2 via its binding to PI(3,5)P_2.

The reviewer's concern that SNX2 recruitment to lysosomes may be PI(3)P-dependent is addressed by the data already shown in our manuscript. PIKfyve inactivation results in increased PI(3)P on lysosomes (**Fig. 5A and 5B**). Therefore, if SNX2 were recruited to lysosomes by PI(3)P, we would have expected increased SNX2 lysosomal recruitment under conditions of PIKfyve inactivation. Instead, SNX2 recruitment to lysosomes only occurred once PIKfyve-dependent production of PI(3,5)P_2 was stimulated using an established YM210636

wash-out method, consistent with $PI(3,5)P_2$-dependent recruitment of SNX2 to lysosomes. To further alleviate the reviewer's concern, we can perform control studies to examine whether PIKfyve generation of $PI(3,5)P_2$ also affects SNX2 localization to early endosomes.

There is also additional literature supporting our contention that $PI(3,5)P_2$ recruits SNX2 to lysosomes, which we can further highlight in the discussion section of the manuscript:

- ***First*,** there are already multiple lines of evidence to support that SNX2 binds $PI(3,5)P_2$:
  - Although *in vitro* protein-lipid overlay assays using the purified PX (phox-homology) domain of SNX2, reported a marked preference for binding to PI(3)P (Zhong et al. *Proc Natl Acad Sci USA*. 2002. PMID: 11997453), subsequent phosphoinositide-binding specificity studies using more physiologically relevant sucrose-loaded liposome-based assays show the full-length SNX2 protein binds primarily to both PI(3)P and $PI(3,5)P_2$ with similar affinity (Carlton et al. *J Cell Sci.* 2007. PMID: 16179610).
  - SNX2 was identified as a putative $PI(3,5)P_2$-binding protein through a comprehensive and unbiased screen of the total cellular $PI(3,5)P_2$ interactome (Catimel et al. *J Proteome Res*. 2008. PMID: 19367725).
  - SNX2 was also identified from proximity interactome analysis of Fig4 and Vac14, two regulatory components of the PIKfyve enzyme complex which is responsible for generating the total cellular pool of $PI(3,5)P_2$ (Qiu et al. *J Proteome Res*. 2021. PMID: 34554760).

- ***Second***, there is evidence that SNX2 also localizes to vesicular structures other than early endosomes.
  - We agree that several studies have shown that a large cellular pool of SNX2 is present at early endosomes where it is part of the retromer complex, and this is dependent upon generation of PI(3)P which binds to the PX- (phox-homology) domain of SNX2 (Mellado et al. *Biol Cell*. 2014. PMID: 25081925, Zhong et al. *Proc Natl Acad Sci USA*. 2002. PMID: 11997453, Gullapalli et al. *Mol Biol Cell*. 2004. PMID: 14978220). However, these studies also show that there are undefined vesicular structures outside of early endosomes that contain SNX2.
  - These reported studies only examined SNX2 localization under basal conditions. As such, this is unlikely to have captured the dynamic recruitment of SNX2 to other intracellular membranes in response to the transient spatiotemporal generation of phosphoinositides. Our study examined the membrane recruitment of SNX2 in response to stimulation of $PI(3,5)P_2$ generation, which was induced by treatment with the reversible PIKfyve inhibitor YM210636 followed by its wash-out. This experimental approach is different to what has been undertaken previously, therefore we have been able to identify SNX2 localization to a new cellular compartment that is dependent upon $PI(3,5)P_2$ generation.

This proposal would have been strengthened if the authors could show colocalization between SNX2 and PI(3,5)P2 on endolysosomes.

This experiment suggested by the reviewer is not feasible due to issues with detection of $PI(3,5)P_2$, which are published :

- There is evidence that the GFP-ML1Nx2 fluorescent biosensor is not specific for $PI(3,5)P_2$ (Hammond et al. *PLoS One*. 2015. PMID: 26460749).
- Takatori *et al*. developed a method to detect $PI(3,5)P_2$ combining freeze-fracture electron microscopy with the use of the recombinant GST-ATG18-4×FLAG protein as a $PI(3,5)P_2$ binding probe, but again this was shown to not be specific for $PI(3,5)P_2$ (Takatori et al. *Traffic*. 2016. PMID: 26563567).
- There is a commercially available $PI(3,5)P_2$ antibody from Echelon Biosciences (product number Z-P035), but in our hands this antibody is not specific for $PI(3,5)P_2$ and this is not routinely used in the phosphoinositide field.

Given these technical challenges, it is standard in the phosphoinositide field not to undertake co-localization of $PI(3,5)P_2$ with its proposed effectors as indicated by the following studies:

- Li et al. *Nat Cell Biol.* 2016. PMID: 26950892
- Rosato et al. *Nat Commun*. 2019. PMID: 31822666
- Dong et al. *Nat Commun*. 2010. PMID: 20802798
- Bissig et al. *Traffic*. 2017. PMID: 28857423.

To address the reviewer's concern, we can instead assess SNX2 co-localization with PIKfyve, which is the sole enzyme that generates $PI(3,5)P_2$ and is used as a surrogate for $PI(3,5)P_2$ in co-localization studies (Rutherford et al. *J Cell Sci*. 2006. PMID: 16954148).

The effect of PIKfyve inhibition on SNX2 localization (Fig 6F) is not convincing since the strong effect of YM210636 on endolysosome morphology makes comparisons difficult. Immunoelectron microscopy could resolve this issue.

We agree with the reviewer that the representative images selected for **Fig. 6F** made it difficult to see SNX2 localization to lysosomes. We can amend this figure with the following representative images, split into individual channels, to more clearly demonstrate SNX2 localization to lysosomes (**Review Figure 1A and 1B**).

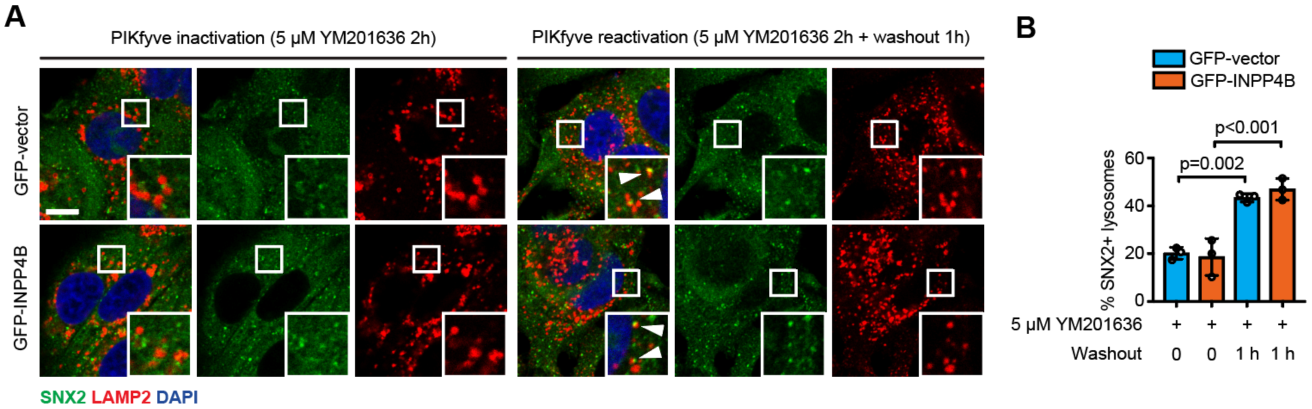

Review Figure 1: SNX2 is recruited to lysosomes in reponse to $PI(3,5)P_2$ generation by PIKfyve
A, **B** MCF-7 cells expressing GFP-INPP4B or GFP-vector were transfected with NT, *SNX1* or *SNX2* siRNA. After 24 hours, cells were fixed and immunostained with LAMP2 antibodies, and co-stained with DAPI and phalloidin (**A**). Data represent the number of LAMP2+ puncta relative to cell area (μm^2) ± SD (n=3 experiments, >40 cells per experiment) (**B**).
Scale bar in **A** is 10 µm.
p values determined by one-way ANOVA in with Tukey post hoc test in **B**

To further reinforce this conclusion, we have commenced the immuno-EM experiments suggested by the reviewer to confirm whether SNX2 is recruited to endolysosomes in response to $PI(3,5)P_2$ generation. We have the capacity and expertise to undertake these types of imaging experiments as shown previously (Rodgers et al. *Nat Commun.* 2021. PMID: 34035258).

4. In general, the authors use RNAi technology to deplete proteins of interest, and, as the authors are well aware of, this technology frequently comes with the disadvantage of off-target effects. Key effects of RNAi-mediated knock-downs must be verified by rescue transfections with RNAi-resistant plasmids.

We can easily perform these experiments. However, our interpretation and conclusions do not rely solely on the use of RNAi technology. We showed INPP4B overexpression versus knockdown had opposing effects on autophagic flux and lysosome homeostasis **(Fig. 1B-G, 2A, 2B, 2D-G, 3A-D, Supplementary Fig. 1D-H, 3A-D)**, whereas PIKfyve knockdown versus small molecule inhibitors had similar effects on lysosome homeostasis **(Supplementary Fig. 6B-E)**. The *INPP4B*/*HRS* shRNA and *PIK3CA* siRNA sequences used in this manuscript have been reported previously with appropriate controls (Fedele et al. *Proc Natl Acad Sci USA*. 2010. PMID: 21127264, Rodgers et al. *Nat Commun.* 2021. PMID: 34035258). To further validate our SNX2 siRNA experiments, we can transfect with siRNAs targeting the 3'-UTR region of SNX2 and perform rescue experiments with GFP-SNX2.

Minor points:

1. The authors should use standard nomenclature for PIK3CA.

2. The authors frequently omit "that" from sentences, making them difficult to comprehend. For instance, "..here we show SNX2 is required..". The text should be revised for clarity.

3. In Figure 7, lysosomes are formed from an "endolysosome" via "endolysosome reformation". This should be "lysosome reformation"?

All points can be addressed with minor text changes.

<u>**Reviewer #2**</u>

Main points:

1. The authors clearly show an impact of INPP4B knockdown and overexpression on basal autophagy, based on analysis of LC3. Can the authors demonstrate that these changes in basal autophagy achieved by INPP4B modulation under nutrient-rich conditions affect cellular responses in any meaningful way? For instance, are their specific autophagy cargoes that accumulate as a result of these changes and cause cellular dysfunction? Examining p62 levels (as the authors do) is a start, but it seems that a more thorough investigation would be in order here. This could be addressed by looking at pathological proteins/autophagy cargoes under more chronic conditions in intact cells in the presence of nutrients or in vivo using KO mice.

We agree with the reviewer that a more thorough investigation of the autophagy cargoes and cellular responses affected by the INPP4B-directed autophagy pathway would strengthen the manuscript. Basal autophagy is critically important for a number of pathways including the clearance of misfolded proteins, which are tagged with ubiquitin chains and delivered to autophagosomes where they are degraded by autophagy. Importantly, inhibition of basal autophagy *in vivo* causes accumulation of ubiquitinated protein aggregates, leading to cytotoxicity and neurodegenerative disease (Hara et al. *Nature*. 2006. PMID: 16625204, Komatsu et al. *Nature*. 2006. PMID: 16625205).

We predict that INPP4B modulation of basal autophagy may also regulate the autophagic clearance of protein aggregates in response to proteotoxic stress. To this end we have generated new preliminary data showing that INPP4B overexpression reduces the accumulation of ubiquiniated protein aggregates in response to

proteotoxic stress induced using puromycin, which induces protein misfolding and aggregation, ± the proteasome inhibitor MG132 (**Review Figure 2A and 2B)**. This is a standard methodology to evaluate effects on the autophagy-dependent removal of protein aggregates (Fan et al. *Autophagy.* 2010. PMID: 20495340, Park et al. *Nat Commun.* 2017. PMID: 28589942). Our preliminary data also suggests that co-treatment with PIKfyve inhibitors prevents the enhanced autophagic degradation of ubiquitinated protein aggregates **(Review Figure 2C and 2D)**, suggesting that INPP4B promotes PIKfyve-dependent lysosomal degradation of protein aggregates.

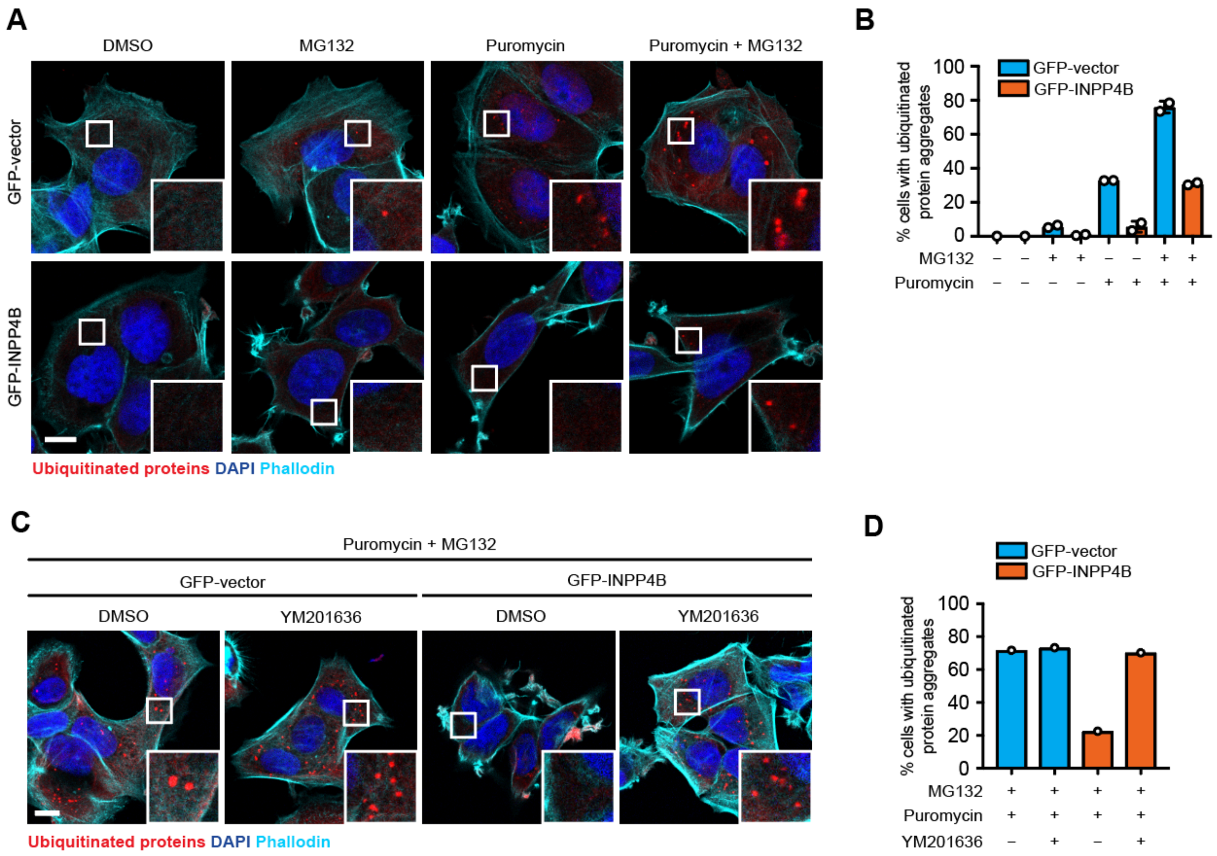

Review Figure 2: INPP4B promotes lysosomal degradation of protein aggregates in response to proteotoxic stress

A MCF-7 cells expressing GFP-INPP4B or GFP-vector were treated for 4 hours with 10 µg/mL puromycin ± 5 µm MG132, or DMSO as a vehicle control. Cells were fixed and immunostained with ubiqutin antibodies (1:100), and co-stained with DAPI and phalloidin.

B Data represent the percentage of cells with ubiquitinated protein aggregates (n>200 cells/experiment).

C MCF-7 cells expressing GFP-INPP4B or GFP-vector were treated for 4 hours with 10 µg/mL puromycin, 5 µm MG132 and either 5 µM YM201636 or DMSO as a vehicle control. Cells were fixed and immunostained with ubiqutin antibodies (1:100), and co-stained with DAPI and phalloidin.

D Data represent the percentage of cells with ubiquitinated protein aggregates (n>200 cells/experiment).

Scale bar in **A**, **C** is 10 µm.

To complete the characterization of this physiologically relevant pathway, we propose to perform the following additional experiments:

- Assess whether INPP4B overexpression and depletion have reciprocal effects on protein aggregate clearance via autophagy, in response to proteotoxic stress
- Co-treatment with bafilomycin as proof of concept that the reduced protein aggregates are a result of increased autophagic degradation, rather than reduced aggregate formation

- Assess cell viability and apoptosis to determine whether INPP4B suppresses cytotoxicity induced by proteotoxic stress

We predict that completion of these experiments will take approximately 2 months.

This new data will highlight the physiological/pathological significance of our signaling pathway, by demonstrating that lysosome reformation from endolysosomes is required for protein quality control and is cytoprotective against proteotoxic stress, which will address the reviewer's concern. This finding will be important given that mutations of PIKfyve regulatory components FIG4 and VAC14 cause neurodegenerative disease, where lysosome involvement is implicated in disease pathogenesis, but lacks mechanistic understanding (Chow et al. *Nature*. 2007. PMID: 17572665, Zhang et al. *Proc Natl Acad Sci USA*. 2007. PMID: 17956977, Zhang et al. *Brain*. 2008. PMID: 18556664, Zolov et al. *Proc Natl Acad Sci USA*. 2012. PMID: 23047693).

We are confident we can address the reviewer's concerns without the use of animal models, in line with other recent studies in the autophagy and lysosome biology field:
- Nthiga et al. *EMBO J.* 2020. PMID: 32525583
- Gu et al. *EMBO J.* 2019. PMID: 31625181
- Baba et al. *EMBO J.* 2019. PMID: 31368593

2. Studies are essentially conducted in MCF-7 cells. They would be more impactful if the key findings were confirmed in independent cell lines.

We agree and can easily examine the effects of this pathway in additional cell lines. MCF-7, HeLa and HEK293 are widely used cell lines in the field of lysosome homeostasis. We have already generated preliminary data showing that INPP4B depletion causes autophagosome accumulation in HeLa and HEK293 cells (**Review Figure 3A-C**), which phenocopies the effects observed with INPP4B depletion in MCF-7 cells. We will also confirm whether the increased autophagosome accumulation in these cells is a result of decreased lysosome formation.

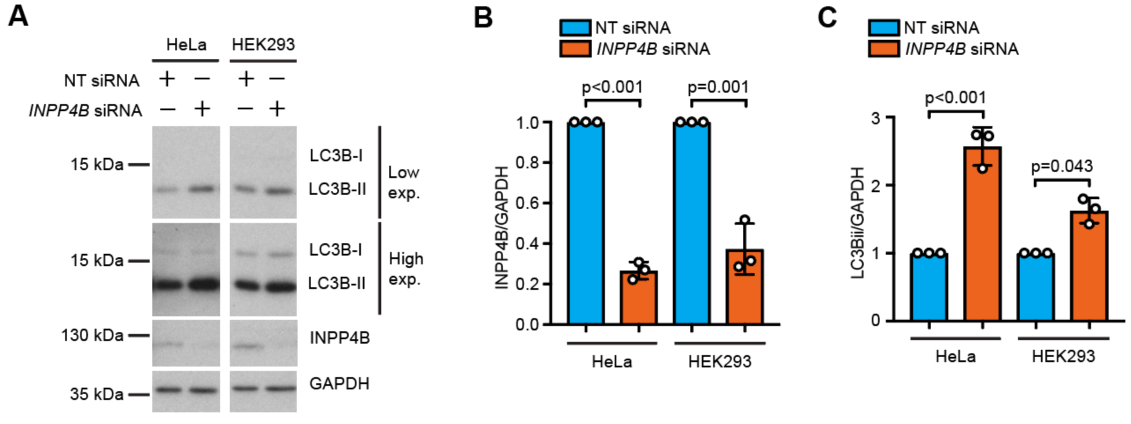

Review Figure 3: INPP4B depletion leads to autophagosome accumulation in HeLa and HEK293 cells
A HeLa or HEK293 cells were transfected with INPP4B or NT siRNA. After 24 hours, cells were lysed and immunoblotted with LC3B and INPP4B antibodies, and GAPDH antibodies as a loading control.
B, C Data represent the mean relative INPP4B **(B)** or LC3B-II **(C)** levels relative to GAPDH.
p values determined by two-tailed unpaired *t* test in **B**, **C**.

Minor points:

3. Some studies have shown that class II PI3Ks contribute to PI3P levels, including in autophagy settings. This should be mentioned in the introduction.

4. Line 136, bafilomycin is defined as an "autophagosome-lysosome fusion inhibitor" but as the authors know, it is primarily a proton pump inhibitor and this should be mentioned; also it should be referred to as "bafilomycin A1".

5. Lane 397, Fig4 is primarily described as a lipid phosphatase rather than a lipid/protein kinase.

We agree with these points, and can easily amend these via text changes in our manuscript.

Dear Dr Mitchell,

Thank you for submitting your revision plan for consideration. I forwarded it to both referees; at this stage though, one was not available to contribute any comments. After reading the feedback from the other referee and discussing your very careful point-by-point response with the editorial team here at EMBO Journal, I have concluded that all of your points are addressable within a realistic time-frame. I would therefore be open to re-sending a revised version of the manuscript to the referees.

At this stage, however, I must stress that this revised manuscript must gain strong support from both referees for us to be able to move forwards towards publication.

I should add that it is EMBO Journal policy to allow only a single round of revision, so the evaluation of your manuscript may only consider this revised version.

We generally allow three months as standard revision time. As a matter of policy, competing manuscripts published during this period will not negatively affect our assessment of the conceptual advance presented by your study. Please contact me as soon as possible upon publication of any related work, to discuss how to proceed. If you envisage needing this three-month window to be widened, please let me know.

Thank you again for the opportunity to consider your work for publication. I look forward to reading your revision.

Best wishes,

William Teale

William Teale, PhD
Editor
The EMBO Journal
w.teale@embojournal.org

Further information is available in our Guide For Authors: https://www.embopress.org/page/journal/14602075/authorguide

RESPONSE TO REVIEWER COMMENTS

Reviewer #1

The manuscript by Rodgers and co-workers builds on recent findings from the authors that the PI 4-phosphatase IMPP4B plays an important role in regulation of endosomal functions. The authors here report that the class I PI 3-kinase PIK3CA (PI3Ka) mediates basal (but not starvation-induced) autophagy via PI(3,4,5)P3, which is subsequently converted to PI(3,4)P2 by a 5-phosphatase at the plasma membrane and then to PI(3)P via INPP4B on late endosomes. This PI(3)P pool was found to act as substrate for the PI(3)P 5-kinase PIKfyve to produce PI(3,5)P2, and SNX2 was suggested to be recruited by this phosphoinositide to mediate endolysosome reformation.
The proposed concept is an interesting one, but parts of the underpinning data are not convincing.

We have addressed all of the concerns raised by reviewer 1 by undertaking the requested experiments **(new figures: Fig 1L, 1M, 4A, 4B, 6F, 6G, Fig EV2O, 2P, Appendix Fig 3C-G, 3J, 3K)**.

Major points:

1. The authors interchangeably refer to "lysosome reformation" and "endolysosome reformation". It is not entirely clear what they mean, and they need to revise the text for consistency, but presumably they refer to the reformation of lysosomes from hybrid organelles that originate from fusion of lysosomes with late endosomes.

The reviewer is correct, our study examines the reformation of lysosomes that are derived from endolysosome membranes (i.e. a hybrid organelle originating from fusion of a late endosome with a lysosome). The name of this process has not been universally defined in the literature, and has been referred to by several different names such as:

- Lysosome reformation from endolysosomes
  - Bissig et al. *Traffic*. 2017. PMID: 28857423
  - Wilden et al. *Biochim Biophys Acta Mol Cell Res*. 2019. PMID: 29966622
- Endocytic lysosome reformation
  - Gan et al. *J Cell Biol*. 2019. PMID: 31235480
  - Yang and Wang. *J Cell Biol*. 2021. PMID: 33950241
- Lysosome reformation
  - Miller et al. *Traffic*. 2015. PMID: 25491304
  - Bright et al. *Curr Biol*. 2005. PMID: 15723798
  - Bright et al. *Curr Biol*. 2016. PMID: 27498570

We agree that our use of the term "*endolysosome reformation*" is confusing. To improve clarity in the manuscript, we have revised this in the text to "*lysosome reformation from endolysosomes*" in line with the described literature above.

The assay they use for measuring "lysosome reformation" is spinning-disk microscopy of cells expressing LAMP1-mCherry and monitoring scission of LAMP1 structures. If the authors want to imply that the fragments being abscised from LAMP1 vesicles represent reformed lysosomes (as opposed to, for instance, recycling tubules), they need to provide evidence for this.

Our use of spinning disk microscopy of cells expressing LAMP1-mCherry is a validated approach for examining lysosome reformation from endolysosomes (Bissig et al. *Traffic*. 2017. PMID: 28857423). The referee proposes that the LAMP1-positive structures observed by spinning disk microscopy may represent

"*recycling tubules*", but this is not consistent with multiple reported studies describing recycling tubules as those originating from early/recycling endosomes, which do not contain LAMP1. For example, LAMP1 does not co-localize with the early endosome protein EEA1 (Kamentseva et al. PLoS One. 2020. PMID: 32357161) or recycling endosome proteins such as the transferrin receptor (Rush and Ceresa. *Mol Cell Endocrinol*. 2013. PMID: 23933150) or KIF13A (Delevoye et al. *Cell Rep*. 2014. PMID: 24462287). To our knowledge, the only LAMP1-positive structures described that undergo scission during growth conditions are lysosomes undergoing reformation.

To further substantiate these findings and address the reviewer's concerns, we have also assessed the number of terminal storage lysosomes, which are lysosomes that are newly formed via reformation from endolysosomes. Terminal storage lysosomes do not contain active hydrolases and can be distinguished from endolysosomes, which do contain active hydrolases (Bissig et al. *Traffic*. 2017. PMID: 28857423, Bright et al. *Curr Biol*. 2016. PMID: 27498570). Using this analysis, we found that *INPP4B* siRNA depletion significantly reduced the number of terminal storage lysosomes **(new Fig 4A and B)**, further supporting our contention that INPP4B is required for lysosome reformation.

There are other additional lines of evidence in the manuscript that support our findings of changes to lysosome reformation. Briefly, our analysis revealed:

- Systematic evaluation of lysosome homeostasis including lysosome number, size and dispersion assessed by LAMP1, LAMP2 and/or MAGIC RED® Cathepsin B staining (**Fig. 3A-H, Fig EV2A-N**)
- A dependence on PIKfyve **(Fig 5D-F, Fig EV4B-E)**, an established regulator of lysosome reformation from endolysosomes (Bissig et al. *Traffic*. 2017. PMID: 28857423).
- Exclusion of other lysosome repopulation pathways (*de novo* lysosome biogenesis and ALR) **(Fig EV3A-F)**.

These approaches are also validated and in line with reported standard methodology used to show deficits in lysosome reformation as outlined in:

- Yu et al. *Nature.* 2010. PMID: 20526321.
- Rong et al. *Nat Cell Biol*. 2012. PMID: 22885770.
- Munson et al. *EMBO J*. 2015. PMID: 26139536
- Rong et al. *Proc Natl Acad Sci USA 2011.* PMID: 21518918
- Bissig et al. *Traffic*. 2017. PMID: 28857423.

2. The idea that basal autophagy is mediated by PIK3CA is interesting, but under physiological conditions PIK3CA activity is likely to fluctuate. This raises the question whether increased PIK3CA activity would increase autophagy, as suggested by the model in Figure 7. The authors should investigate this.

The reviewer is correct in that PIK3CA activity fluctuates under physiological conditions dependent on growth factor stimulation of cells/tissues. However in most cell culture models, PIK3CA activity is high due to the abundance of growth factors in the culture media, and the frequent presence of PIK3CA mutations that constitutively active the protein in many cancer cell lines. The standard method to assess PIK3CA activity in cultured cells requries the starvation of cells overnight, followed by acute growth factor/insulin stimulation to reactivate growth factor receptors which in turn activate PI3K. However, these starvation conditions are also likely to activate starvation-induced autophagy.

We attempted to assess whether increased PIK3CA activity affects basal autophagy by expressing hyperactive HA-PIK3CA^{H1047R} mutant protein in HeLa cells, which express endogenous wild-type PIK3CA, and evaluating LC3B-I/LC3B-II levels by immunoblotting. However, we were not able to achieve sufficient transfection efficiency with this plasmid to reliably interpret these experimental results. Instead, we inhibited

PIK3CA using the PI3Kα inhibitor BYL719 in MCF-7 cells, which harbor a hyperactivating PIK3CA^{E545K} mutation. Despite suppressing AKT/mTOR activation, PI3Kα inhibition had minimal effect on basal autophagy in GFP-vector cells but significantly increased the reduced LC3B-II levels in GFP-INPP4B cells **(new Fig 1L and M)**. This data is consistent with our findings that INPP4B promotes lysosome formation downstream of PI3Kα under growth conditions **(Fig 3G and H, new Fig EV2O and P)**.

We have also highlighted in our discussion that "*the contribution of the class I PI3K signaling network to autophagy is complex, with evidence that class I PI3K effector proteins can promote or suppress autophagy*" (line 565). We have also amended our graphical figure **(Fig 7, now Fig 8)** to show that class I PI3K activation of AKT/mTOR may also affect autophagy, consistent with graphical figures from other studies (Dou et al. *J Cell Biol*. 2010. PMID: 21059846; Dou et al. *Mol Cell*. 2013. PMID: 23434372), to further highlight the complexity of this pathway.

3. The most speculative part of the manuscript is the last section that deals with PIKfyve and SNX2. As the authors mention, SNX2 binds to both PI(3)P and PI(3,4)P2 in addition to PI(3,5)P2, and binding to PI(3)P seems to have highest affinity. It is therefore not clear how SNX2 would be specifically recruited to endolysosomes via PI(3,5)P2 binding, especially because SNX2 has been reported to localize to early endosomes (for instance, Mellado et al., Biol.Cell, 2014). This proposal would have been strengthened if the authors could show colocalization between SNX2 and PI(3,5)P2 on endolysosomes.

We agree with the reviewer that there is an established role for SNX2 at endosomes via PI(3)P binding. However, this does not preclude a distinct functional role for SNX2 at lysosomes via PI(3,5)P_2 binding. Below, we provide a point by point argument to address the reviewer's concerns.

<u>SNX2 binds to PI(3,5)P_2 with high affinity</u>
Initial *in vitro* protein-lipid overlay assays, using the purified PX (phox-homology) domain of SNX2, reported a marked preference for binding to PI(3)P (Zhong et al. *Proc Natl Acad Sci USA*. 2002. PMID: 11997453). However, subsequent phosphoinositide-binding specificity studies, using more physiologically relevant sucrose-loaded liposome-based assays, show the full-length SNX2 protein binds to both PI(3)P and PI(3,5)P_2 with <u>similar affinity</u> (Carlton et al. *J Cell Sci*. 2007. PMID: 16179610). SNX2 binding to PI(3,5)P_2 has also been independently identified from PI(3,5)P_2 interactomes (Catimel et al. *J Proteome Res*. 2008. PMID: 19367725), and proximity interactomes of Fig4 and Vac14, the two regulatory components of the PIKfyve enzyme complex that generates all cellular PI(3,5)P_2 (Qiu et al. *J Proteome Res*. 2021. PMID: 34554760).

<u>SNX2 is recruited to lysosomes by PI(3,5)P_2 and not PI(3)P</u>
PIKfyve inactivation results in increased PI(3)P on lysosomes (**Fig. 5A and 5B**). Therefore, if SNX2 were recruited to lysosomes by PI(3)P, we would have expected increased SNX2 lysosomal recruitment under conditions of PIKfyve inactivation. Instead, SNX2 recruitment to lysosomes occurred only during experimental conditions that allowed PIKfyve-dependent production of PI(3,5)P_2, which was stimulated using an established YM201636 wash-out method **(Fig 6D and E)**. We have also performed control studies showing that PIKfyve inhibition did not affect SNX2 localization to early endosomes **(new Appendix Fig 3J and K)**, suggesting that PIKfyve does not affect the pool of PI(3)P that recruits SNX2 to endosomes.

<u>PI(3,5)P_2 and SNX2 co-localization experiments are not feasible</u>
This experiment suggested by the reviewer is unfortunately not feasible due to reported problems with detection of PI(3,5)P_2:

- There is evidence that the GFP-ML1Nx2 fluorescent biosensor is not specific for PI(3,5)P_2 (Hammond et al. *PLoS One*. 2015. PMID: 26460749).

- Takatori *et al.* developed a method to detect $PI(3,5)P_2$ combining freeze-fracture electron microscopy with the use of the recombinant GST-ATG18-4×FLAG protein as a $PI(3,5)P_2$ binding probe, but again this was shown not to be specific for $PI(3,5)P_2$ (Takatori et al. *Traffic*. 2016. PMID: 26563567).
- There is a commercially available $PI(3,5)P_2$ antibody from Echelon Biosciences (product number Z-P035), but in our hands this antibody is not specific for $PI(3,5)P_2$ and this is not routinely used.

Given these technical challenges, it is standard practise currently in the phosphoinositide field not to undertake $PI(3,5)P_2$ co-localization experiments as described in the following papers:

- Li et al. *Nat Cell Biol.* 2016. PMID: 26950892
- Rosato et al. *Nat Commun*. 2019. PMID: 31822666
- Dong et al. *Nat Commun*. 2010. PMID: 20802798
- Bissig et al. *Traffic*. 2017. PMID: 28857423.

To address the reviewer's concern, we have instead assessed SNX2 co-localization with PIKfyve, which is the sole enzyme that generates $PI(3,5)P_2$ and has been used as a surrogate for $PI(3,5)P_2$ in co-localization studies (Rutherford et al. *J Cell Sci*. 2006. PMID: 16954148). Using super resolution microscopy, we demonstrated that PIKfyve and SNX2 co-localized together on a sub-population of lysosomes **(new Figure 6G)**, strengthening our contention that SNX2 is recruited to lysosomes by $PI(3,5)P_2$ generated by PIKfyve.

The effect of PIKfyve inhibition on SNX2 localization (Fig 6F) is not convincing since the strong effect of YM210636 on endolysosome morphology makes comparisons difficult. Immunoelectron microscopy could resolve this issue.

We agree with the reviewer that the representative images selected for **Fig. 6F (now Fig 6D)** make it difficult to see SNX2 localization to lysosomes. We have amended this figure with representative images of lower fluorescence intensity, split into individual channels, to more clearly demonstrate SNX2 localization to lysosomes.

To further reinforce this conclusion, we have performed immuno-electron microscopy analysis as requested by the reviewer showing that a pool of GFP-SNX2 localized to lysosome membranes during PIKfyve reactivation **(new Fig 6F)**.

4. In general, the authors use RNAi technology to deplete proteins of interest, and, as the authors are well aware of, this technology frequently comes with the disadvantage of off-target effects. Key effects of RNAi-mediated knock-downs must be verified by rescue transfections with RNAi-resistant plasmids.

We agree with the reviewer that RNAi can have off-target effects. To further validate our *SNX2* siRNA experiments, we assessed lysosomes in cells co-transfected with siRNAs targeting the 3'-UTR region of *SNX2* and siRNA-resistant recombinant GFP-SNX2 **(new Appendix Fig 3C-G)**. This analysis showed that the reduced lysosomes numbers observed with *SNX2* depletion could be rescued by recombinant SNX2 expression. Our interpretation and conclusions of other RNAi experiments presented in the paperdo not rely solely on the use of RNAi technology. We showed INPP4B overexpression versus knockdown had opposing effects on autophagic flux, lysosome homeostasis and proteotoxic stress response **(Fig. 1B-G, 2A, 2B, D-G, 3A-F, 7A-H, Fig EV5A-D, Appendix Fig 1D-H, 3A-D)**. Small molecular inhibitors of PIKfyve or PIK3CA had similar effects to siRNA depletion on lysosome homeostasis **(new Fig EV2O and P) (Fig 3G, H, 5D-F, Fig EV4B-E)**. The *INPP4B*/*Hrs* shRNA and *PIK3CA* siRNA sequences used in this manuscript have been reported previously and validated with appropriate controls (Fedele et al. *Proc Natl Acad Sci USA*. 2010. PMID: 21127264, Rodgers et al. *Nat Commun.* 2021. PMID: 34035258).

Minor points:

1. The authors should use standard nomenclature for PIK3CA.

We have clarified in the text that "*PI3Kα is a heterodimer consisting of a catalytic p110α subunit and a p85 regulatory subunit*" (line 282), whereas *PIK3CA* "*encodes the p110α catalytic subunit of PI3Kα*" (line 284). This is the standard nomenclature used in the field (Vasan et al. *Science*. 2019. PMID: 31699932; Rodgers et al. *Nat Commun*. 2021. PMID: 34035258).

2. The authors frequently omit "that" from sentences, making them difficult to comprehend. For instance, "..here we show SNX2 is required..". The text should be revised for clarity.

We have amended these grammatical errors.

3. In Figure 7, lysosomes are formed from an "endolysosome" via "endolysosome reformation". This should be "lysosome reformation"?

The reviewer is correct, we have now amended the text in this figure to "*lysosome reformation*" **(now Fig 8).**

Reviewer #2

In this manuscript, Rodgers et al focus on mechanisms controlling lysosome "repopulation" under nutrient-rich conditions and their impact on basal autophagy. They identify a phosphoinositide pathway operating on the endolysosomal system where type I PI 3-Kinase alpha controls lysosome repopulation by generating a pool of PI(3,4,5)P3 that is converted to PI(3,4)P2 and PI(3)P, prior to phosphorylation to PI(3,5)P2 by lipid kinase PIKfyve. Specifically, the authors show, using a combination of silencing and overexpression approaches, that PI3Kalpha-derived PI(3)P is generated by PI phosphatase INPP4B on late endosomes and maintained on these organelles as they mature into lysosomes. A key effector of PI(3,5)P2 in this context is determined to be SNX2, a SNX-BAR protein involved in lysosomal tubulation/fission. Importantly, they show that this pathway is only relevant under nutrient-rich conditions and thus for basal autophagy, whereas nutrient deprivation-induced autophagy relies more on the better understood Vps34 pathway.

This is an interesting manuscript that provides additional evidence for the existence of Vps34-independent sources of PI(3)P and their physiological relevance in cells, following up on a number of studies from the same group. Mechanisms controlling basal autophagy are poorly understood and this study offers a novel perspective, through a careful mechanistic dissection of the phosphoinositide pathway and their associated signaling components. While the study is generally well conducted and interpreted, it does not address in depth the physiological or pathophysiological consequence of reduced basal autophagy, as achieved by manipulation of the INPP4B pathway. Additionally, it is unclear whether findings are limited to MCF-7 cells or whether they have broader implications. This limits the impact of the study and in the absence of the following suggested revisions, it may be more appropriate for specialized cell biology journals.

To further explore the physiological/pathophysiological consequences of reduced basal autophagy, we have generated new data showing that INPP4B/PIKfyve are required for autophagic degradation of protein aggregates in response to proteotoxic stress **(new figures: Fig 7A-H Fig EV5A-D)**, which has physiological relevance for neurological disease. We also provide evidence that INPP4B regulates lysosome homeostasis, basal autophagy and the proteotoxic stress response in additional independent cell lines **(new figures: Fig 4A, 4B, 7A-D, 7G, 7H, Fig EV2C, 2D, Appendix Fig 1D-F, 3C-G)**.

Main points:

1. The authors clearly show an impact of INPP4B knockdown and overexpression on basal autophagy, based on analysis of LC3. Can the authors demonstrate that these changes in basal autophagy achieved by INPP4B modulation under nutrient-rich conditions affect cellular responses in any meaningful way? For instance, are their specific autophagy cargoes that accumulate as a result of these changes and cause cellular dysfunction? Examining p62 levels (as the authors do) is a start, but it seems that a more thorough investigation would be in order here. This could be addressed by looking at pathological proteins/autophagy cargoes under more chronic conditions in intact cells in the presence of nutrients or in vivo using KO mice.

We agree with the reviewer that a more thorough investigation of the autophagy cargoes and cellular responses affected by the INPP4B/PIKfyve-directed autophagy pathway would strengthen the manuscript. Basal autophagy is critically important for a number of pathways including the clearance of misfolded proteins, which are tagged with ubiquitin chains and delivered to autophagosomes where they are degraded by autophagy. Importantly, inhibition of basal autophagy *in vivo* causes accumulation of ubiquitinated protein aggregates, leading to cytotoxicity and neurodegenerative disease (Hara et al. *Nature*. 2006. PMID: 16625204, Komatsu et al. *Nature*. 2006. PMID: 16625205).

We predicted that INPP4B/PIKfyve modulation of basal autophagy may also regulate the autophagic clearance of protein aggregates in response to proteotoxic stress. We therefore assessed ubiquitinated protein aggregate accumulation in cells in response to proteotoxic stress using puromycin treatment, which causes protein misfolding and aggregation, ± the proteasome inhibitor MG132. This is a standard methodology to evaluate effects on the autophagy-dependent removal of protein aggregates (Fan et al. *Autophagy.* 2010. PMID: 20495340, Park et al. *Nat Commun.* 2017. PMID: 28589942). We found that *INPP4B* siRNA or PIKfyve inhibition (YM201636) increased protein aggregate accumulation in puromycin-treated cells **(new Fig 7A-D)**. INPP4B overexpression reduced the accumulation of ubiquiniated protein aggregates in puromycin-treated cells **(new Fig EV5A and B)**. To delineate whether this was due to protein aggregate clearance by the ubiquitin proteasome, cells were also co-treated with the proteasome inhibitor MG132. INPP4B overexpression also reduced protein aggregation under these conditions **(new Fig EV5A and B)**, which was reversed by concomitant YM201636 or bafilomycin A1 treatment **(new Fig 7E and F, Fig EV5C and D)**. Collectively, this data indicates that INPP4B/PIKfyve regulation of lysosome reformation is required for autophagic degradation of protein aggregates under nutrient-rich conditions.

Prolonged proteotoxic stress activates apoptosis and cell death, and defective proteotoxic stress response causes cytotoxicity leading to a range of pathological conditions. We found that *INPP4B* siRNA depletion or PIKfyve inhibition significantly decreased the viability of puromycin-treated cells **(new Fig 7G and H)**.

We do not have access to *Inpp4b* knockout mice, but this new data does address the reviewer's concerns, in line with other recent studies in the autophagy and lysosome biology field (Nthiga et al. *EMBO J.* 2020. PMID: 32525583; Gu et al. *EMBO J.* 2019. PMID: 31625181; Baba et al. *EMBO J.* 2019. PMID: 31368593).

This new data also highlights the pathological significance of INPP4B/PIKfyve-dependent lysosome reformation by demonstrating its requirement for protein quality control and cytoprotection against proteotoxic stress. These findings are important given that mutations in PIKfyve regulatory components FIG4 and VAC14 cause neurological disease, and lysosome involvement is implicated in disease pathogenesis but the mechanisms are unknown (Chow et al. *Nature*. 2007. PMID: 17572665, Zhang et al. *Proc Natl Acad Sci USA*. 2007. PMID: 17956977, Zhang et al. *Brain*. 2008. PMID: 18556664, Zolov et al. *Proc Natl Acad Sci USA*. 2012. PMID: 23047693).

2. Studies are essentially conducted in MCF-7 cells. They would be more impactful if the key findings were confirmed in independent cell lines.

We agree that examining the effects of this pathway in additional independent cell lines would strengthen our manuscript. MCF-7, HeLa and HEK293 are widely used cell lines in the field of lysosome homeostasis. We have generated new data showing that *INPP4B* depletion causes autophagosome accumulation in HeLa and HEK293 cells **(new Appendix Fig 1D-F)**, which phenocopies the effects observed with *INPP4B* depletion in MCF-7 cells. We have also shown that *INPP4B* depletion in HeLa cells reduces the number of lysosomes **(new Fig EV2C and D)** and terminal storage lysosomes **(new Fig 4A and B)**, and that *INPP4B* depletion or PIKfyve inhibition enhances protein aggregate accumulation and cytotoxicity in response to proteotoxic stress **(new Fig 7A-D, G, H)**. Furthermore, we show that *SNX2* 3'-UTR siRNA depletion reduces lysosomes in HeLa cells, which can be rescued by expression of siRNA-resistant GFP-SNX2 **(new Appendix Fig 3C-G)**. This suggests that INPP4B regulation of lysosome formation, basal autophagy and proteotoxic stress response is conserved across multiple independent cell lines

Minor points:

3. Some studies have shown that class II PI3Ks contribute to PI3P levels, including in autophagy settings. This should be mentioned in the introduction.

We have mentioned in the introduction that "*the class II PI3K, PI3KC2α, is also required for shear stress-induced autophagy via PI(3)P generation at primary cilia, whereas PI3KC2β contributes to autophagic degradation during starvation via PI(3,4)P_2 generation on lysosomes*" (line 81).

4. Line 136, bafilomycin is defined as an "autophagosome-lysosome fusion inhibitor" but as the authors know, it is primarily a proton pump inhibitor and this should be mentioned; also it should be referred to as "bafilomycin A1".

We have amended bafilomycin to "*bafilomycin A1*", and describe this as "*the V-ATPase inhibitor bafilomycin A1, which prevents autophagosome-lysosome fusion*" (line 167), in line with the field (Mauvezin et al. *Nat Commun*. 2015. PMID: 25959678).

5. Lane 397, Fig4 is primarily described as a lipid phosphatase rather than a lipid/protein kinase.

We have amended this typographical error to describe Fig4 as a "*lipid/protein phosphatase*" (line 373 and 578), in line with recent studies (Lees et al. *Mol Cell*. 2020. PMID: 33098764).

2nd Revision - Editorial Decision 15th Jul 2022

Dear Christina,

We have now received re-review reports from both referees. As you will see from their comments below, you have addressed their concerns satisfactorily.

There are some remaining editorial points which need to be addressed. Our production checks are currently being conducted, so this list may not be exhaustive; I will update you on these checks next week.
In the meantime, would you please:

- remove the Author Contributions section from the manuscript and use the free text boxes in our online system beneath each contributing author's name to add specific details on the author's contribution. More information on this is available in our guide to authors.

- Rename the Conflict of Interest statement as the "Disclosure Statement and Competing Interests", and

- use an alphabetical reference format, limiting each reference to ten author names before "et al."

Please let me know if you have any further queries.

Best wishes,

William

Dr William Teale, PhD
Editor|EMBO Journal

Referee #1:

The authors have attempted to address all the concerns I had, and I appreciate that some of these were challenging to address. Overall the revised version of the manuscript makes a much more compelling case for the main conclusions drawn, and I am happy to support publication in EMBO Journal.

Referee #2:

The authors have addressed all my questions and concerns.

Dear Prof. Facundo Batista and Dr. William Teale,

We thank you for the opportunity to submit a revised manuscript that addresses the comments raised by the reviewers. We have undertaken the requested experiments and include these new results in the revised manuscript and in the response to the reviewer's comments.

The issues raised by the reviewers mainly relate to $PI(3,5)P_2$ recruitment of SNX2 to lysosomes, the physiological consequences of reduced basal autophagy, and whether the findings are limited to MCF-7 cells.

Specifically, we have performed further experiments as requested by the reviewers demonstrating:

1. Immuno-electron and super resolution microscopy analysis of SNX2 recruitment to lysosomes via $PI(3,5)P_2$ generated by PIKfyve.

2. INPP4B/PIKfyve-dependent lysosome reformation are required for autophagic clearance of protein aggregates and cell survival during proteotoxic stress.

3. INPP4B regulates lysosome formation, basal autophagy and proteotoxic stress response in multiple independent cell lines.

We also provide a point-by-point detailed answer to each reviewers' comments, and zip files containing all raw source data and uncropped blots with the manuscript.

The review process has improved the quality of the study and the manuscript and we thank you and the reviewers for your expert comments. We hope the revised manuscript is now suitable for publication in *The EMBO Journal.*

Yours sincerely,

Christina Mitchell

3rd Revision - Editorial Decision 21st Jul 2022

Dear Christina,

I am pleased to inform you that your manuscript has been accepted for publication in the EMBO Journal.

Congratulations to you and your team on a really nice study!

Please note that it is EMBO Journal policy for the transcript of the editorial process (containing referee reports and your response letter) to be published as an online supplement to each paper. If you do NOT want this, you will need to inform the Editorial Office via email immediately. More information is available here:
https://www.embopress.org/page/journal/14602075/authorguide#transparentprocess

Your manuscript will be processed for publication in the journal by EMBO Press. Manuscripts in the PDF and electronic editions of The EMBO Journal will be copy edited, and you will be provided with page proofs prior to publication. Please note that supplementary information is not included in the proofs.

You will be contacted by Wiley Author Services to complete licensing and payment information. The required 'Page Charges Authorization Form' is available here: https://www.embopress.org/pb-assets/embo-site/tej_apc.pdf - please download and complete the form and return to embopressproduction@wiley.com

Should you be planning a Press Release on your article, please get in contact with embojournal@wiley.com as early as possible, in order to coordinate publication and release dates.

If you have any questions, please do not hesitate to call or email the Editorial Office. Thank you for your contribution to The EMBO Journal.

Yours sincerely,

William

William Teale, PhD
Editor
The EMBO Journal
w.teale@embojournal.org

** Click here to be directed to your login page: https://emboj.msubmit.net

EMBO Press Author Checklist

Corresponding Author Name: Christina Mitchell	
Journal Submitted to: The EMBO Journal	
Manuscript Number: EMBOJ-2021-110398	

USEFUL LINKS FOR COMPLETING THIS FORM
[The EMBO Journal - Author Guidelines]
[EMBO Reports - Author Guidelines]
[Molecular Systems Biology - Author Guidelines]
[EMBO Molecular Medicine - Author Guidelines]

Reporting Checklist for Life Science Articles (updated January 2022)
This checklist is adapted from Materials Design Analysis Reporting (MDAR) Checklist for Authors. MDAR establishes a minimum set of requirements in transparent reporting in the life sciences (see Statement of Task: 10.31222/osf.io/9sm4x). Please follow the journal's guidelines in preparing your manuscript.
Please note that a copy of this checklist will be published alongside your article.

Abridged guidelines for figures
1. Data
The data shown in figures should satisfy the following conditions:
- the data were obtained and processed according to the field's best practice and are presented to reflect the results of the experiments in an accurate and unbiased manner.
- ideally, figure panels should include only measurements that are directly comparable to each other and obtained with the same assay.
- plots include clearly labeled error bars for independent experiments and sample sizes. Unless justified, error bars should not be shown for technical replicates.
- if n<5, the individual data points from each experiment should be plotted. Any statistical test employed should be justified.
- Source Data should be included to report the data underlying figures according to the guidelines set out in the authorship guidelines on Data Presentation.

2. Captions
Each figure caption should contain the following information, for each panel where they are relevant:
- a specification of the experimental system investigated (eg cell line, species name).
- the assay(s) and method(s) used to carry out the reported observations and measurements.
- an explicit mention of the biological and chemical entity(ies) that are being measured.
- an explicit mention of the biological and chemical entity(ies) that are altered/varied/perturbed in a controlled manner.
- the exact sample size (n) for each experimental group/condition, given as a number, not a range;
- a description of the sample collection allowing the reader to understand whether the samples represent technical or biological replicates (including how many animals, litters, cultures, etc.).
- a statement of how many times the experiment shown was independently replicated in the laboratory.
- definitions of statistical methods and measures:
  - common tests, such as t-test (please specify whether paired vs. unpaired), simple χ^2 tests, Wilcoxon and Mann-Whitney tests, can be unambiguously identified by name only, but more complex techniques should be described in the methods section;
  - are tests one-sided or two-sided?
  - are there adjustments for multiple comparisons?
  - exact statistical test results, e.g., P values = x but not P values < x;
  - definition of 'center values' as median or average;
  - definition of error bars as s.d. or s.e.m.

Please complete ALL of the questions below.
Select "Not Applicable" only when the requested information is not relevant for your study.

Materials

Newly Created Materials	Information included in the manuscript?	In which section is the information available? (Reagents and Tools Table, Materials and Methods, Figures, Data Availability Section)
New materials and reagents need to be available; do any restrictions apply?	Yes	Materials and methods

Antibodies	Information included in the manuscript?	In which section is the information available? (Reagents and Tools Table, Materials and Methods, Figures, Data Availability Section)
For **antibodies** provide the following information:<br>- Commercial antibodies: RRID (if possible) or supplier name, catalogue number and or/clone number<br>- Non-commercial: RRID or citation	Yes	Appendix Table S1

DNA and RNA sequences	Information included in the manuscript?	In which section is the information available? (Reagents and Tools Table, Materials and Methods, Figures, Data Availability Section)
**Short novel DNA or RNA including primers, probes**: provide the sequences.	Not Applicable	

Cell materials	Information included in the manuscript?	In which section is the information available? (Reagents and Tools Table, Materials and Methods, Figures, Data Availability Section)
**Cell lines:** Provide species information, strain. Provide accession number in repository **OR** supplier name, catalog number, clone number, and/**OR** RRID.	Yes	Materials and methods
**Primary cultures:** Provide species, strain, sex of origin, genetic modification status.	Not Applicable	
Report if the cell lines were recently **authenticated** (e.g., by STR profiling) and tested for mycoplasma contamination.	Yes	Materials and methods

Experimental animals	Information included in the manuscript?	In which section is the information available? (Reagents and Tools Table, Materials and Methods, Figures, Data Availability Section)
**Laboratory animals or Model organisms:** Provide species, strain, sex, age, genetic modification status. Provide accession number in repository **OR** supplier name, catalog number, clone number, **OR** RRID.	Not Applicable	
**Animal observed in or captured from the field:** Provide species, sex, and age where possible.	Not Applicable	
Please detail **housing and husbandry conditions**.	Not Applicable	

Plants and microbes	Information included in the manuscript?	In which section is the information available? (Reagents and Tools Table, Materials and Methods, Figures, Data Availability Section)
**Plants:** provide species and strain, ecotype and cultivar where relevant, unique accession number if available, and source (including location for collected wild specimens).	Not Applicable	
**Microbes:** provide species and strain, unique accession number if available, and source.	Not Applicable	

Human research participants	Information included in the manuscript?	In which section is the information available? (Reagents and Tools Table, Materials and Methods, Figures, Data Availability Section)
If collected and within the bounds of privacy constraints report on age, sex and gender or ethnicity for all study participants.	Not Applicable	

Core facilities	Information included in the manuscript?	In which section is the information available? (Reagents and Tools Table, Materials and Methods, Figures, Data Availability Section)
If your work benefited from core facilities, was their service mentioned in the acknowledgments section?	Yes	Acknowledgments

Design

Study protocol	Information included in the manuscript?	In which section is the information available? (Reagents and Tools Table, Materials and Methods, Figures, Data Availability Section)
If study protocol has been **pre-registered, provide DOI in the manuscript**. For clinical trials, provide the trial registration number **OR** cite DOI.	Not Applicable	
Report the **clinical trial registration number** (at ClinicalTrials.gov or equivalent), where applicable.	Not Applicable	

Laboratory protocol	Information included in the manuscript?	In which section is the information available? (Reagents and Tools Table, Materials and Methods, Figures, Data Availability Section)
Provide DOI OR other citation details if **external detailed step-by-step protocols** are available.	Yes	Materials and methods

Experimental study design and statistics	Information included in the manuscript?	In which section is the information available? (Reagents and Tools Table, Materials and Methods, Figures, Data Availability Section)
Include a statement about **sample size** estimate even if no statistical methods were used.	Yes	Materials and methods
Were any steps taken to minimize the effects of subjective bias when allocating animals/samples to treatment (e.g. **randomization procedure**)? If yes, have they been described?	Yes	Materials and methods
Include a statement about **blinding** even if no blinding was done.	Yes	Materials and methods
Describe **inclusion/exclusion criteria** if samples or animals were excluded from the analysis. Were the criteria pre-established?<br><br>If sample or data points were omitted from analysis, report if this was due to attrition or intentional exclusion and provide justification.	Not Applicable	
For every figure, are **statistical tests** justified as appropriate? Do the data meet the assumptions of the tests (e.g., normal distribution)? Describe any methods used to assess it. Is there an estimate of variation within each group of data? Is the variance similar between the groups that are being statistically compared?	Yes	Figure legends, Materials and methods

Sample definition and in-laboratory replication	Information included in the manuscript?	In which section is the information available? (Reagents and Tools Table, Materials and Methods, Figures, Data Availability Section)
In the figure legends: state number of times the experiment was **replicated** in laboratory.	Yes	Figure legends
In the figure legends: define whether data describe **technical or biological replicates**.	Yes	Figure legends

Ethics

Ethics	Information included in the manuscript?	In which section is the information available? (Reagents and Tools Table, Materials and Methods, Figures, Data Availability Section)
Studies involving **human participants**: State details of **authority granting ethics approval** (IRB or equivalent committee(s), provide reference number for approval.	Not Applicable	
Studies involving **human participants**: Include a statement confirming that **informed consent** was obtained from all subjects and that the experiments conformed to the principles set out in the WMA Declaration of Helsinki and the Department of Health and Human Services Belmont Report.	Not Applicable	
Studies involving **human participants:** For publication of **patient photos**, include a statement confirming that consent to publish was obtained.	Not Applicable	
Studies involving experimental **animals**: State details of **authority granting ethics approva**l (IRB or equivalent committee(s), provide reference number for approval. Include a statement of compliance with ethical regulations.	Not Applicable	
Studies involving **specimen and field samples:** State if relevant **permits** obtained, provide details of authority approving study; if none were required, explain why.	Not Applicable	

Dual Use Research of Concern (DURC)	Information included in the manuscript?	In which section is the information available? (Reagents and Tools Table, Materials and Methods, Figures, Data Availability Section)
Could your study fall under dual use research restrictions? Please check biosecurity documents and list of **select agents and toxins** (CDC): https://www.selectagents.gov/sat/list.htm	Not Applicable	
If you used a select agent, is the security level of the lab appropriate and reported in the manuscript?	Not Applicable	
If a study is subject to dual use research of concern regulations, is the name of the **authority granting approval and reference number** for the regulatory approval provided in the manuscript?	Not Applicable	

Reporting

The MDAR framework recommends adoption of discipline-specific guidelines, established and endorsed through community initiatives. Journals have their own policy about requiring specific guidelines and recommendations to complement MDAR.

Adherence to community standards	Information included in the manuscript?	In which section is the information available? (Reagents and Tools Table, Materials and Methods, Figures, Data Availability Section)
State if relevant guidelines or checklists (e.g., **ICMJE, MIBBI, ARRIVE, PRISMA**) have been followed or provided.	Not Applicable	
For **tumor marker prognostic studies,** we recommend that you follow the **REMARK** reporting guidelines (see link list at top right). See author guidelines, under 'Reporting Guidelines'. Please confirm you have followed these guidelines.	Not Applicable	
For **phase II and III randomized controlled trials**, please refer to the **CONSORT** flow diagram (see link list at top right) and submit the CONSORT checklist (see link list at top right) with your submission. See author guidelines, under 'Reporting Guidelines'. Please confirm you have submitted this list.	Not Applicable	

Data Availability

Data availability	Information included in the manuscript?	In which section is the information available? (Reagents and Tools Table, Materials and Methods, Figures, Data Availability Section)
Have **primary datasets** been deposited according to the journal's guidelines (see 'Data Deposition' section) and the respective accession numbers provided in the Data Availability Section?	Not Applicable	
Were **human clinical and genomic datasets** deposited in a public access-controlled repository in accordance to ethical obligations to the patients and to the applicable consent agreement?	Not Applicable	
Are **computational models** that are central and integral to a study available without restrictions in a machine-readable form? Were the relevant accession numbers or links provided?	Not Applicable	
If publicly available data were reused, provide the respective **data citations in the reference list**.	Not Applicable	
